# BacPROTACs mediate targeted protein degradation in bacteria

**DOI:** 10.1016/j.cell.2022.05.009

**Published:** 2022-06-23

**Authors:** Francesca E. Morreale, Stefan Kleine, Julia Leodolter, Sabryna Junker, David M. Hoi, Stepan Ovchinnikov, Anastasia Okun, Juliane Kley, Robert Kurzbauer, Lukas Junk, Somraj Guha, David Podlesainski, Uli Kazmaier, Guido Boehmelt, Harald Weinstabl, Klaus Rumpel, Volker M. Schmiedel, Markus Hartl, David Haselbach, Anton Meinhart, Markus Kaiser, Tim Clausen

**Affiliations:** 1 Research Institute of Molecular Pathology, Vienna Biocenter, 1030 Vienna, Austria; 2 University of Duisburg-Essen, Center of Medical Biotechnology, Faculty of Biology, 45141 Essen, Germany; 3 Saarland University, Organic Chemistry I, 66123 Saarbrücken, Germany; 4 Boehringer Ingelheim RCV GmbH & Co KG, 1120 Vienna, Austria; 5 Max Perutz Laboratories, Vienna Biocenter, 1030 Vienna, Austria; 6 Medical University of Vienna, 1030 Vienna, Austria

**Keywords:** targeted protein degradation, AAA proteases, bacterial PROTACs, antimicrobials

## Abstract

Hijacking the cellular protein degradation system offers unique opportunities for drug discovery, as exemplified by proteolysis-targeting chimeras. Despite their great promise for medical chemistry, so far, it has not been possible to reprogram the bacterial degradation machinery to interfere with microbial infections. Here, we develop small-molecule degraders, so-called BacPROTACs, that bind to the substrate receptor of the ClpC:ClpP protease, priming neo-substrates for degradation. In addition to their targeting function, BacPROTACs activate ClpC, transforming the resting unfoldase into its functional state. The induced higher-order oligomer was visualized by cryo-EM analysis, providing a structural snapshot of activated ClpC unfolding a protein substrate. Finally, drug susceptibility and degradation assays performed in mycobacteria demonstrate *in vivo* activity of BacPROTACs, allowing selective targeting of endogenous proteins via fusion to an established degron. In addition to guiding antibiotic discovery, the BacPROTAC technology presents a versatile research tool enabling the inducible degradation of bacterial proteins.

## Highlights

• BacPROTACs reprogram bacterial ClpCP proteases to degrade neo-substrates
• Substrate binding converts latent ClpC into active, higher-order complexes with ClpP
• Incorporation of cyclomarin as head group yields BacPROTACs active in mycobacteria
• BID can eliminate proteins of interest *in vivo*

## Graphical abstract

**Figure:**
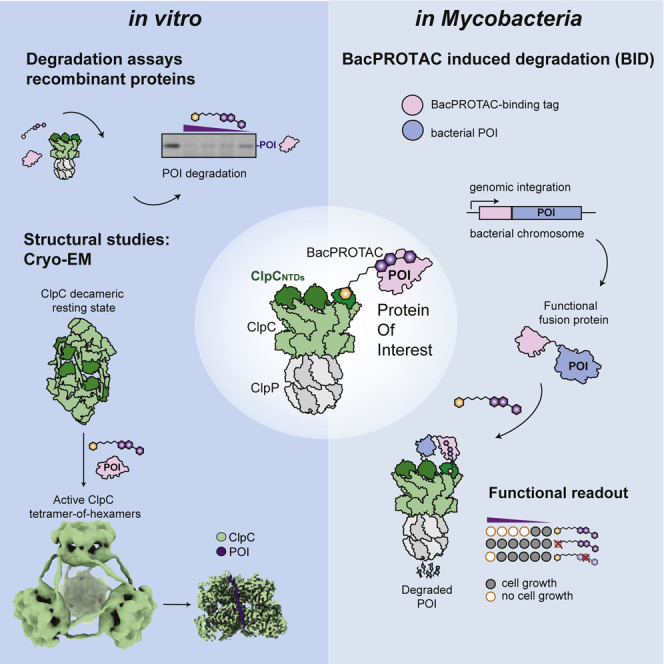

## Teaser

Small-molecule adaptors, BacPROTACs, redirect bacterial ClpCP protease to target neo-substrates in a highly specific manner and expand targeted protein degradation technology to bacteria.

## Introduction

Technological advances in proteomics, chemical biology, and high-throughput screening campaigns have boosted drug discovery across therapeutic areas. Unfortunately, however, these advances have thus far not equally translated into the development of novel antibacterial agents (Lewis, 2020; Payne et al., 2007; Plackett, 2020; Tommasi et al., 2015). This discrepancy is most obvious when considering the small number of antibiotics that have been discovered in the last 50 years (Lewis, 2020). Beyond economic hurdles, the advancement of new antibiotics is challenged by the low permeability of the bacterial envelope, and the limited number of microbial proteins that can be specifically inhibited without off-target effects. The difficulties in finding effective antimicrobials are further compounded by the speed at which pathogens are developing resistance to existing drugs. In light of this unequal arms race, the return of bacterial pandemics is a real threat, and innovative strategies to combat infections are urgently needed (Lewis, 2020).

An emerging concept in drug discovery is the induced elimination of target proteins. Engineered chemicals can now interfere with various degradation pathways, redirecting the lysosomal (Banik et al., 2020), the autophagy (Li et al., 2019; Takahashi et al., 2019), or the ubiquitin-proteasome systems (Sakamoto et al., 2001) to target specified proteins. The most prominent synthetic “degraders” are the proteolysis targeting chimeras (PROTACs): bi-functional small molecules that contain a binding head for an E3 ubiquitin ligase and a chemical moiety to engage a protein of interest (POI) (Sakamoto et al., 2001). By bringing the E3 ligase and POI into proximity, PROTACs promote POI ubiquitination and consequent degradation by the proteasome. Protein degraders have various advantages over classical inhibitors. For example, they exhibit higher efficacy due to their catalytic mode of action. Moreover, they allow targeting of virtually any cellular protein and their modular architecture allows protein ligands to be repurposed to build degraders (Churcher, 2018; Hanzl and Winter, 2020; Schapira et al., 2019). Despite this promise, PROTAC technology is so far restricted to the ubiquitin tagging system of eukaryotes and has yet to be transferred to degradation pathways in bacteria. Fulfilling this last objective would provide an attractive strategy to design modulators of protein function and a platform for antibiotics discovery.

**Figure 1:**
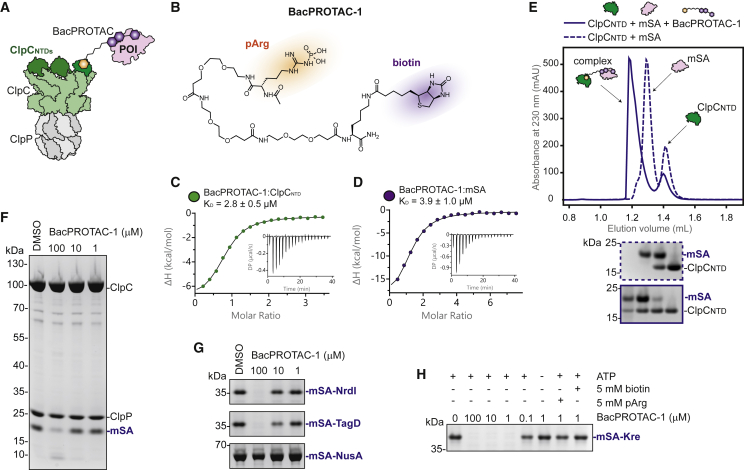
*In vitro* reprogramming of *B. subtilis* ClpCP by BacPROTAC-1 (A) Schematic representation of the BacPROTAC approach, hijacking the ClpCP protease. (B) Chemical structure of BacPROTAC-1, designed to tether the model substrate mSA to the ClpC_NTD_ receptor domain. (C and D) ITC titrations of BacPROTAC-1 against ClpC_NTD_ and mSA. Calculated K_D_s and their standard deviation are indicated (n ≥ 3). (E) SEC analysis of a stoichiometric mSA:ClpC_NTD_ mixture in the presence (solid line) or absence (dashed line) of BacPROTAC-1. An SDS-PAGE analysis (lower) confirms the formation of the indicated ternary complex. (F–H) SDS-PAGE analysis of *in vitro* degradation assays. mSA alone and mSA fusion proteins (mSA-NrdI, mSA-TagD, mSA-NusA, mSA-Kre) are degraded by ClpCP in a BacPROTAC-1-dependent manner (2-h incubation, DMSO used as control). (H) For mSA-Kre, further controls indicate that degradation is ATP dependent and that pArg or biotin block the BacPROTAC-1 induced degradation. See also Figure S1. Uncropped images of SDS-PAGE gels are shown in Data S1.

Although ubiquitin is unique to eukaryotic cells, some bacteria utilize a similar system for targeted protein degradation. Phosphorylated arginine residues (pArg) serve as a degradation signal that is recognized by the ClpC:ClpP (ClpCP) protease, a quasi-proteasomal particle critical for microbial protein quality control, stress tolerance, and pathogenicity (Trentini et al., 2016). ClpCP is present in gram-positive bacteria and in mycobacteria. In the latter, the equivalent ClpC1P1P2 protease is essential for survival *in vitro* and in macrophages (DeJesus et al., 2017; Rengarajan et al., 2005). ClpC carries out its quality control function by acting as an ATP-driven unfoldase that selects certain client proteins and translocates them into the protease compartment formed by ClpP. The ClpC protomer contains an amino-terminal domain (ClpC_NTD_), two AAA (ATPases associated with diverse cellular activities) domains termed D1 and D2, and a coiled coil (M domain) inserted into D1. In the active ClpC hexamer, the D1 and D2 domains form two stacked ATPase rings that power substrate unfolding and translocation. The ClpC_NTD_ on top of the D1 ring controls access to the unfoldase, providing docking sites for adaptor proteins including MecA (Schlothauer et al., 2003; Wang et al., 2011). In addition, the ClpC_NTD_ receptor domain recognizes pArg-tagged substrates that account for ∼30% of the whole ClpP degradome in gram-positive bacteria (Trentini et al., 2016). Compared with the eukaryotic proteasome, which recognizes a complex poly-ubiquitin signal, the degradation signal recognized by ClpCP is much simpler: a plain phosphate group attached to an arginine residue of the client protein (Trentini et al., 2016). Thus, we investigated whether the ClpCP degradation machine can be directly reprogrammed by small molecules. We reasoned that bi-functional adaptors could tether neo-substrates to the ClpC_NTD_ receptor domain, priming them for degradation (Figure 1A). Our results provide a proof of concept that such BacPROTACs can be developed and are active *in vivo*, enabling the inducible, selective, and efficient degradation of target proteins in bacteria.

## Results

### Development of BacPROTACs that reprogram ClpCP

To enable ClpC-mediated protein degradation, we designed BacPROTACs composed of a POI ligand, a chemical linker and a ClpC_NTD_ anchor. The anchor initially consisted of a peptidic pArg derivative mimicking the bacterial degradation tag. In order to test the targeting of a neo-substrate, we used monomeric streptavidin (mSA) as a model protein. We synthesized BacPROTAC-1 (Figure 1B), connecting the pArg moiety to biotin, a high affinity mSA ligand (Lim et al., 2013). The linker attachment points were designed based on high-resolution crystal structures of pArg:ClpC_NTD_ (Trentini et al., 2016) and biotin:mSA (Demonte et al., 2013). Isothermal titration calorimetry (ITC) measurements confirmed that BacPROTAC-1 binds mSA and ClpC_NTD_ with high affinity (K_D_s of 3.9 and 2.8 μM; Figures 1C and 1D), whereas analytical size-exclusion chromatography (SEC) runs revealed the formation of a stable ternary complex (Figure 1E). To analyze whether the induced spatial proximity is sufficient to trigger degradation, we reconstituted the *Bacillus subtilis* ClpCP protease *in vitro* and monitored mSA digestion at different BacPROTAC concentrations. Incubation with 100 μM BacPROTAC-1 led to selective mSA depletion (Figure 1F), indicating that the bacterial ClpCP can be reprogrammed by a pArg-containing chemical adaptor. Notably, however, efficient degradation was only observed at concentrations that were higher than the measured affinities among individual components. This discrepancy likely reflects distinct requirements for substrate recruitment and translocation processes carried out by the ClpC unfoldase.

**Figure S1:**
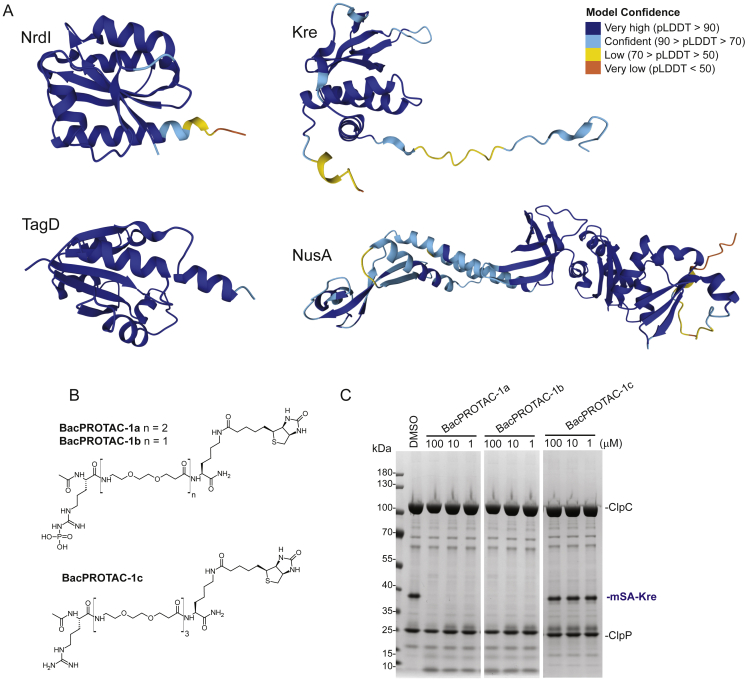
Model substrates and bi-functional compounds used to address BacPROTAC-1-induced activity of *B. subtilis* ClpCP, related to Figure 1 (A) Ribbon cartoon of analysis of *B. subtilis* proteins used in mSA fusion constructs generated by AlphaFold (Jumper et al., 2021; Varadi et al., 2022) and colored by per-residue confidence score (pLDDT). The selected proteins include: NrdI (a flavodoxin-like protein component of ribonucleoside reductases) (Cotruvo and Stubbe, 2010), TagD (glycerol-3-phosphate cytidyltranferase involved in teichoic acid synthesis) (Park et al., 1993), NusA (transcription factor involved in pausing/termination) (Gusarov and Nudler, 2001) and Kre (also known as YkyB, a regulator of the competence transcription factor ComK) (Gamba et al., 2015). PDB structures available for *B. subtilis* NrdI bound to riboflavin monophosphate (PDB: 1RLJ) and *B. subtilis* TagD dimer bound to cytidine-5′-triphosphate (PDB: 1COZ), superposed well with the AlphaFold models. (B) Chemical structure of BacPROTAC-1 analogs with shorter linker lengths (BacPROTAC-1a and -1b), and of the control compound BacPROTAC-1c lacking the phosphate group essential for ClpC interaction. (C) SDS-PAGE analysis of *in vitro* degradation assays. mSA-Kre is degraded by ClpCP in the presence of BacPROTAC-1a and -1b (2-h incubation, DMSO used as control), while the control compound BacPROTAC-1c does not induce degradation.

To follow up on this and analyze the influence of substrate-specific properties on ClpCP activity, we cloned various mSA fusion proteins. We selected four *B. subtilis* targets that have been identified as physiological, pArg-labeled ClpCP substrates (Trentini et al., 2016), with the aim of finding favorable structural features facilitating degradation. Three of these proteins (NrdI, TagD, and NusA) adopt rather compact protein folds, whereas Kre is predicted to possess a 28-amino acid long unstructured C-terminal tail (Figure S1A). Biochemical assays, monitoring the BacPROTAC-1 dependent degradation of the four mSA fusion proteins by ClpCP, revealed pronounced differences (Figures 1G and 1H). mSA-Kre was by far the best substrate, being degraded by 1 μM BacPROTAC-1. These data indicated that, beyond BacPROTAC affinities, structural features of recruited neo-substrates significantly affect degradation efficiency. Consistent with the reported ClpCP preference for proteins having intrinsically disordered termini (Lunge et al., 2020), the efficient degradation of mSA-Kre may relate to its unstructured C-terminal stretch, functioning as initiator site for substrate unfolding and translocation. A similar mechanism has been proposed for the functionally related 26S proteasome, which requires an unstructured region to efficiently degrade ubiquitinated proteins (Bard et al., 2019; Inobe et al., 2011; Prakash et al., 2004). To confirm that degradation is specifically induced by BacPROTAC-1, we hindered substrate recruitment by adding the isolated binding moieties pArg or biotin to the reaction (Figure 1H). In the presence of these compounds, mSA-Kre was not digested by ClpCP, confirming that BacPROTAC-mediated ternary complex formation is essential for degradation. Likewise, a degrader impaired in binding to ClpC (BacPROTAC-1c, containing a non-phosphorylated arginine residue) did not induce substrate degradation (Figures S1B and S1C). Finally, we investigated the impact of linker design on degrading efficiency, a parameter shown to influence the activity of bi-functional PROTACs, defining E3 ligase-substrate interactions (Churcher, 2018). We thus synthesized two additional analogs containing shorter chemical spacers between the pArg and biotin moieties (BacPROTAC-1a and -1b; Figure S1B). However, degradation efficiency was not significantly influenced (Figure S1C), suggesting that linker length is not as relevant for ClpCP to engage with neo-substrates.

Taken together, our results show that pArg-containing BacPROTACs can recruit POIs to the ClpC_NTD_ domain and promote their degradation by the ClpCP protease. In addition to the binding characteristics of the chemical adaptor, intrinsic properties of target proteins seem to play an equally important role in determining degradation efficiency.

### BacPROTAC binding induces ClpC reassembly and activation

**Figure 2:**
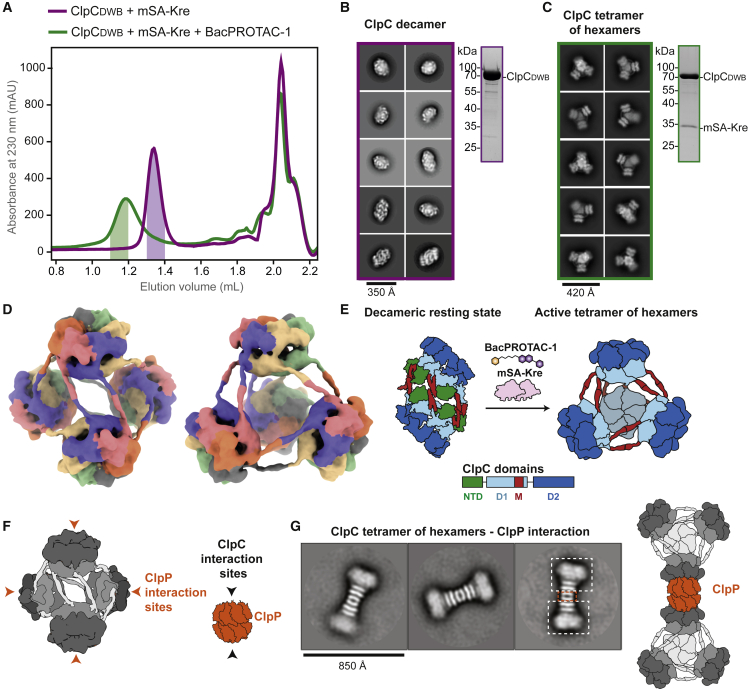
BacPROTAC-1 induces formation of an active ClpC oligomer (A) SEC analysis of a stoichiometric ClpC_DWB_:mSA-Kre mixture in the presence (green) or absence (magenta) of BacPROTAC-1. Fractions used for EM analysis are highlighted. SDS-PAGE gels demonstrate the co-elution of ClpC_DWB_ and mSA-Kre in the presence of BacPROTAC-1. (B) Representative 2D class averages obtained from negative-stained EM images, showing that *B. subtilis* ClpC forms a decameric complex representing the resting state. (C) Representative 2D class averages showing that in the presence of substrate and BacPROTAC-1, ClpC transforms into a 24-mer, composed of four hexamers present in functional form. (D) Refined 3D model (10 Å resolution) of the tetramer of ClpC hexamers, shown in two orientations. Individual ClpC protomers are colored differently. (E) Schematic representation of the BacPROTAC-induced conversion of the inactive ClpC decamer into the active higher-order particle (24-mer), using a domain-based coloring mode as illustrated below. (F) Schematic representation of the tetramer of ClpC hexamers and the double heptameric ClpP ring. Sites of interactions are indicated by arrows. (G) Representative 2D class averages of the ClpC_DWB_:ClpP complex from negative-stained EM images with a schematic model, visualizing the structural organization of the ClpC_24_-ClpP_14_-ClpC_24_ complex. See also Figure S2.

**Figure S2:**
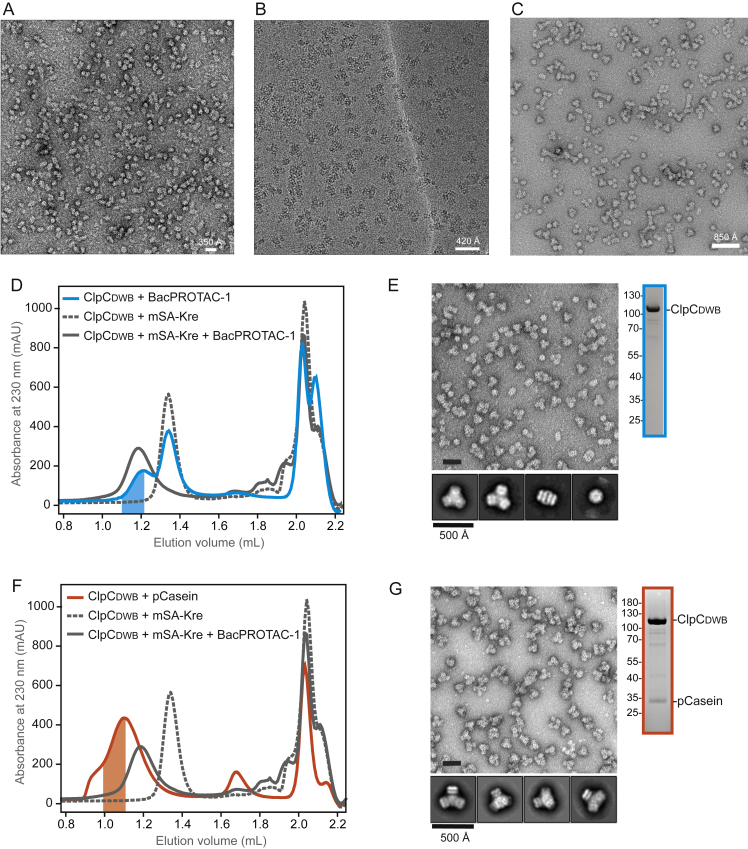
BacPROTAC-1 induces formation of an active ClpC oligomer, related to Figure 2 (A) Negative-stained EM analysis of *B. subtilis* ClpC_DWB_ in the absence of BacPROTAC-1: a representative micrograph from the 1,004 collected (scale bar, 350 Å). (B) Cryo-EM analysis of *B. subtilis* ClpC_DWB_ in the presence of BacPROTAC-1 and mSA-Kre: a representative micrograph from the 4,455 collected (scale bar, 420 Å). (C) Negative-stain EM analysis of *B. subtilis* ClpC_DWB_:ClpP in the presence of BacPROTAC-1: a representative micrograph of the 84 micrographs collected (scale bar, 850 Å). (D) SEC analysis of ClpC_DWB_ in the presence of BacPROTAC-1 (cyan) compared with the SEC analysis reported in Figure 2A of a stoichiometric ClpC_DWB_:mSA-Kre mixture in the presence (gray) or absence (gray, dotted line) of BacPROTAC-1. The fraction used for negative-stained EM analysis is highlighted. A representative micrograph area and SDS-PAGE gel are shown in (E), together with representative 2D class averages. (F) SEC analysis of ClpC_DWB_ in the presence of equimolar amounts of pArg-β-casein (orange) compared with the SEC analysis reported in Figure 2A. The fraction used for negative-stain EM analysis is highlighted. A representative micrograph area and SDS-PAGE gel are shown in (G), together with representative 2D class averages.

Housekeeping proteases and chaperones that target aberrant proteins need to be carefully controlled to prevent concomitant damage to functional proteins in the cell. A study addressing the regulation of the *Staphylococcus aureus* ClpC, a close relative to the *B. subtilis* unfoldase, revealed the existence of a ClpC resting state, a decamer with disrupted AAA rings (Carroni et al., 2017). Interaction with the adaptor protein MecA destabilizes the decamer and promotes assembly of the functional hexamer with an active arrangement of ATPase units (Carroni et al., 2017). In contrast to adaptor-mediated activation, it is unclear how the majority of ClpC substrates labeled with pArg trigger remodeling of the latent ClpC decamer. To elucidate this general activation mechanism—and more specifically, the way in which it is mimicked by a pArg-based degrader—we performed a structural analysis of ClpC in complex with BacPROTAC-1 and the mSA-Kre fusion protein. To stabilize ATP-mediated contacts between ClpC protomers, we used a catalytically inactive mutant (E280A/E618A, referred to as ClpC_DWB_) that binds ATP but does not hydrolyze it. When we added ClpC_DWB_ and mSA-Kre in stoichiometric amounts to a SEC column, ClpC_DWB_ and mSA-Kre eluted separately (Figure 2A). Similar to the *S. aureus* protein, isolated *B. subtilis* ClpC_DWB_ was present in its resting state, the decamer, as visualized by negative-staining EM (Figures 2B and S2A). Incubation with BacPROTAC-1 led to the co-elution of mSA and ClpC_DWB_, pointing to the formation of a stable ternary complex. To our surprise, however, the estimated size of the BacPROTAC-induced complex was far from compatible with the predicted size of substrate-bound ClpC_DWB_ hexamer (Figure 2A). Instead, the resulting complex eluted as a higher-order ClpC oligomer with a molecular mass beyond 2 MDa. EM analysis of the substrate engaged ClpC revealed the formation of a tetramer of ClpC hexamers, arranged in almost perfect tetrahedral symmetry (Figures 2C and S2B). Tetramer formation did not result from residual tetramerization propensity of the mSA-Kre substrate, as partial conversion into this active state was also observed in the presence of BacPROTAC-1 alone (Figures S2D and S2E). A similar quaternary arrangement has been reported for a chimeric ClpC (*M. tuberculosis* ClpC1_NTD_ fused to *S. aureus* D1-D2 AAA core) incubated with the antibiotic cyclomarin A (CymA) (Maurer et al., 2019). However, in that case, 2D class averages of negative-stained EM images could not reveal structural details of the tetrahedral assembly. BacPROTAC-tethered mSA-Kre, which mimics a trapped substrate, yielded seemingly better-defined particles of the activated state. Cryo-EM analysis visualized the overall organization of the higher-order ClpC unfoldase complex at 10 Å resolution (Figure 2D; Table 1). In this state, the D2 rings of the four ClpC hexamers project outward such that they can interact with the ClpP protease. The substrate-bound ClpC_NTD_ domains are located in the center of the particle but are too flexible to be defined by EM density. Most strikingly, the four ClpC hexamers interact with each other via their coiled-coil M domains, establishing a net of helix pairs holding the particle together (Figure 2D). As the M domains are known to stabilize the resting state of ClpC (Carroni et al., 2017), their BacPROTAC-induced reorientation might disassemble the latent form and promote formation of active hexamers stabilized within a supramolecular assembly (Figure 2E). To validate the functional relevance of the remodeled ClpC complex, we analyzed ClpC oligomers in the presence of an arginine-phosphorylated substrate. To this end, we phosphorylated beta-casein by the McsB kinase and incubated the isolated pArg-casein with ClpC. Binding of pArg-casein transformed the ClpC decamer into higher-order complexes, most of which were present as tetrahedral oligomers, visualized by negative-stained EM analysis (Figures S2F and S2G). These data unveil a distinct mechanism of regulating AAA ATPase function. Labeled substrates bind to ClpC, remodeling the resting decamer into an active higher-order complex. This mechanism that directly couples substrate binding with unfoldase activation can be hijacked by BacPROTACs allowing delivery of neo-substrates to ClpC.

**Table 1:** Cryo-EM data collection, refinement, and validation statistics

	ClpC hexamer (EMDB-11707) (PDB: 7ABR)	Tetramer of CpC hexamers (EMDB-11708)
Data collection and processing		
Magnification	75,000	75,000
Voltage (kV)	300	300
Electron exposure (e–/Å^2^)	54	54
Defocus range (μm)	1.3–4.5	1.3–4.5
Pixel size (Å)	1.058	1.058
Symmetry imposed	C1	T
Initial particle images (No.)	1,034,627	1,173,558
Final particle images (No.)	212,314	87,575
Map resolution (Å) FSC threshold	3.7 0.143	10 0.143
Map resolution range (Å)	3.4–5.5	–

Refinement		
Initial model used (PDB Code)	3J3U	–
Model resolution (Å) FSC threshold	3.7 0.5	–
Map sharpening *B* factor (Å^2^)	−210	
Model composition Non-hydrogen atoms Protein residues Ligands	27,209 3445 ADP: 4, ATP: 7	–
*B* factors (Å^2^) Protein Ligand	67.93 53.21	–
R.m.s. deviations Bond lengths (Å) Bond angles (°)	0.011 1.221	–
Validation MolProbity score Clashscore Poor rotamers (%)	2.33 19.31 0.51	–
Ramachandran plot Favored (%) Allowed (%) Disallowed (%)	90.13 9.64 0.24	–
EMRinger score	1.86	–

To assess whether the reassembled ClpC contributes to proteolysis, we analyzed complex formation with the double-ring protease ClpP. Notably, mixing equimolar amounts of ClpP and ClpC in the presence of BacPROTAC led to protein precipitation, presumably reflecting the formation of an extended, mesh-like assembly that results from multivalent interactions between ClpC and ClpP particles (Figure 2F). We therefore used sub-stoichiometric amounts of ClpP to visualize ClpCP complexes. Addition of BacPROTAC-1 induced formation of defined 5-MDa particles that could be resolved by negative-stained EM (Figure S2C). The 2D class averages illustrate the structural organization of a giant ClpC_24_-ClpP_14_-ClpC_24_ complex, in which tetrahedral ClpC oligomers bind to both sides of the ClpP protease (Figure 2G). These structural data also confirm that the BacPROTAC-induced oligomer is an active ClpC state, composed of functional hexamers poised to interact with the ClpP protease.

**Figure 3:**
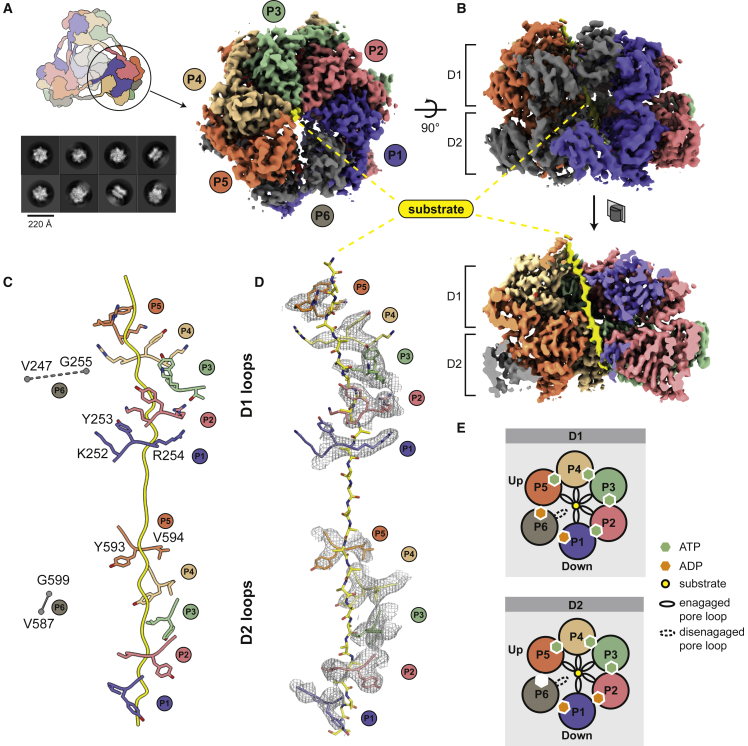
Cryo-EM structure of the activated ClpC hexamer in complex with a BacPROTAC-tethered substrate (A) Representative 2D class averages from cryo-EM images are shown together with the final 3D map at a resolution of 3.7 Å. The density is colored according to subunits that are termed P1–P6. The substrate captured in the central channel is shown in yellow. (B) Side views of the substrate-bound ClpC. The lower one shows the cross section of the hexamer highlighting the substrate threaded through the two D1 and D2 rings of ClpC. The substrate was well defined by cryo-EM density over the entire passage of the central channel (80 Å). (C) Arrangement of primary D1 and D2 pore loops engaging the substrate (peptide backbone shown in yellow). The P6 pore loops, which were not in contact with the substrate, were too flexible to be modeled into the cryo-EM density. Their approximate position is indicated by flanking residues. (D) Cryo-EM density of the tyrosine-bearing pore loops overlaid with the final model. (E) Schematic representation of nucleotide states and substrate engagement of the six ClpC protomers. Nucleotides were assigned based on cryo-EM density and distance matrices in the active site (Figure S3D). See also Figure S3.

**Figure S3:**
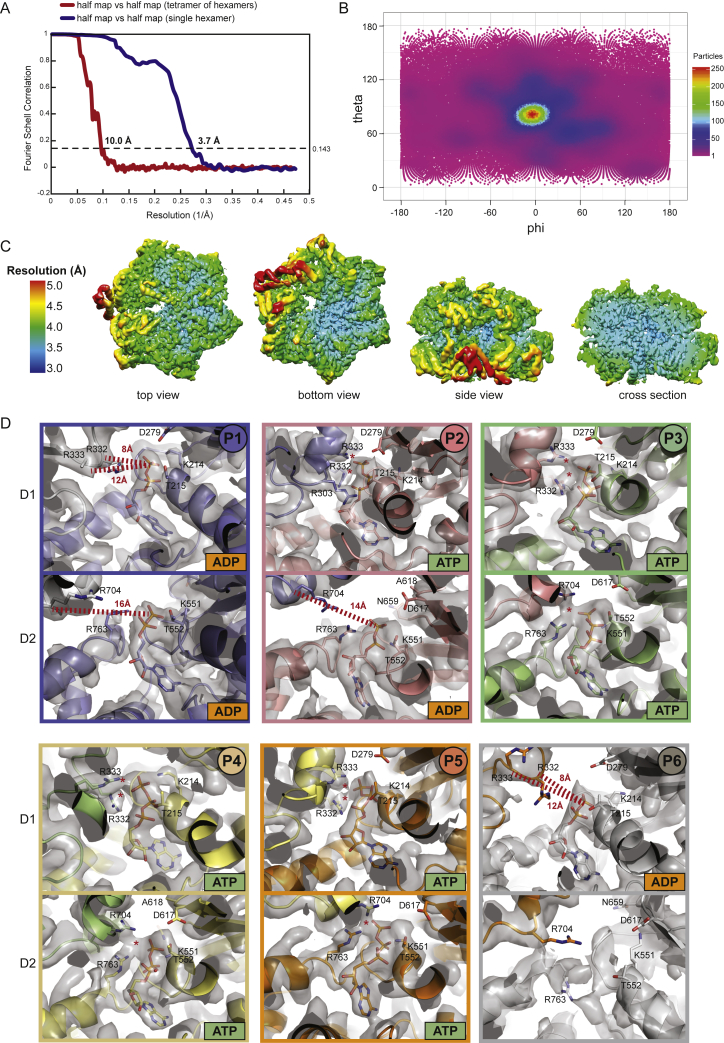
Cryo-EM structure of the activated ClpC hexamer in complex with a BacPROTAC-tethered substrate, related to Figure 3 (A) FSC curves of the final maps obtained by cryo-EM analysis, showing a resolution of 10 Å for the tetramer of hexamers map and 3.7 Å for the single hexamer map. (B) Angular distribution of the particles used to reconstruct the single hexamer map. (C) Local resolution map for the single hexamer in different orientations. (D) Nucleotide-binding sites of the six ClpC_DWB_ protomers. The different panels show the modeled nucleotide in each active site pocket and some of the crucial residues involved in ATP hydrolysis and ATP/ADP interaction. Contacts between ATP γ-phosphate and Arg fingers in D1 (R332-R333) and D2 (R704) are indicated (^∗^) for the ATP-bound sites, while distances between Arg fingers Cα and ADP β-phosphate are shown for ADP-bound sites. The cryo-EM map is represented as gray surface around the modeled protein structure.

To resolve the functional units of the activated unfoldase at higher resolution, we performed a focused cryo-EM analysis of the single hexamers (Figure 3A). The resulting 3D map had an overall resolution of 3.7 Å (Figures S3A–S3C; Table 1) and allowed us to build an atomic model of the substrate-bound ClpC complex (Figure 3; Video S1). The most prominent feature of the cryo-EM reconstruction is a well-defined, 80-Å-long density that penetrates the entire ClpC pore, proceeding from the top of the D1 to the bottom of the D2 ring (Figure 3B). Although it is not possible to discern side chains, the density should represent the protein backbone of the captured mSA-Kre substrate, comprising 26 residues present in an extended conformation. The six ClpC subunits adopt a spiral arrangement, engaging the substrate in a similar manner to that observed in the related double-ring AAA unfoldases ClpA, ClpB, and Hsp104 (Gates et al., 2017; Lopez et al., 2020; Rizo et al., 2019). Five ClpC protomers (P1–P5, P1 as lowermost and P5 uppermost unit in the AAA ring) interact with the substrate through conserved tyrosine-bearing pore loops in the D1 and D2 domains, while the “seam” protomer (P6) is detached from the substrate, transitioning from the bottom to the top position of the spiral (Figures 3C and 3D). Nucleotide states were assigned on the basis of the EM density and the position of the so-called arginine fingers from the neighboring subunit (Figure S3D). Accordingly, the D1 and D2 ATPase rings act in a closely coordinated manner to translocate substrates through the central pore. In both rings, the substrate-engaging P2–P5 protomers are present in an ATP-bound state (except P2-D2), whereas protomers P1 and P6 accommodate ADP or are present in an apo state. These structural data are consistent with the previously suggested “hand-over-hand” translocation mechanism (Puchades et al., 2020), according to which nucleotide exchange is coupled with substrate release and upward movement of the seam protomer P6 to rebind ATP and substrate (Figure 3E). By reorienting in a concerted manner, the ClpC protomers move stepwise from one end of the spiral to the other, dragging the captured substrate down the central channel (Puchades et al., 2020). Cycles of substrate binding, translocation, and release are driven by ATP hydrolysis at the lowermost P1 protomer of the spiral. In conclusion, the cryo-EM structure of the reconstituted ternary complex provides a snapshot of ClpC in the process of unfolding a BacPROTAC-tethered substrate. The structure of the ClpC 24-mer indicates that the pArg mark serves not only as a degradation signal but also mediates higher-order oligomer formation and activation of ClpC. The developed BacPROTAC containing a pArg moiety triggers this remodeling mechanism and thus functions not only as a chemical adaptor but also as an activator of the ClpCP protease.

Video S1. Cryo-EM structure of activated ClpC in complex with substrate, related to Figure 3 The video shows the map of the tetramer of ClpC hexamers (10 Å) and the map obtained refining a single ClpC hexamer (3.7 Å). Both maps are colored by ClpC protomer. Continuous density is observed for a substrate peptide (colored yellow) along the ClpC channel, surrounded by Tyr-containing pore loops, as described in the main text.

### Extending the BacPROTAC approach to mycobacteria

**Figure S4:**
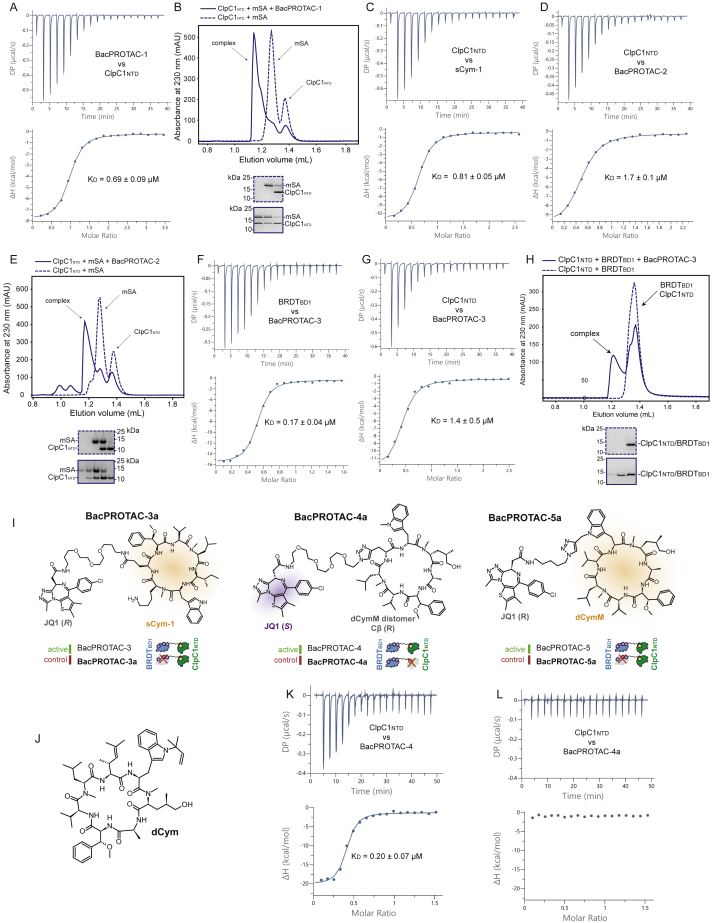
Characterization of compounds and BacPROTACs binding ClpC1_NTD_, related to Figure 4 (A) Representative ITC titration of BacPROTAC-1 (400 μM loaded in the syringe) against ClpC1_NTD_ (22 μM loaded in the cell); reported K_D_ value represents the average ± SD of three independent measurements. (B) SEC analysis of a stoichiometric mSA:ClpC1_NTD_ mixture in the presence (solid line) or absence (dashed line) of BacPROTAC-1. SDS-PAGE analysis (lower) confirms the formation of the indicated ternary complex. (C) Representative ITC titration of ClpC1_NTD_ (406 μM loaded in the syringe) against sCym-1 (30 μM loaded in the cell), reported K_D_ value represents the average ± SD of three independent measurements. (D) Representative ITC titration of ClpC1_NTD_ (356 μM loaded in the syringe) against BacPROTAC-2 (30 μM loaded in the cell), reported K_D_ value represents the average ± SD of two independent measurements. (E) SEC analysis of a stoichiometric mSA:ClpC1_NTD_ mixture in the presence (solid line) or absence (dashed line) of BacPROTAC-2. An SDS-PAGE analysis (lower panel) confirms the formation of the indicated ternary complex. (F) Representative ITC titration of BRDT_BD1_ (124 μM loaded in the syringe) against BacPROTAC-3 (15 μM loaded in the cell); reported K_D_ value represents the average ± SD of three independent measurements. (G) Representative ITC titration of ClpC1_NTD_ (392 μM loaded in the syringe) against BacPROTAC-3 (30 μM loaded in the cell); reported K_D_ value represents the average ± SD of five independent measurements. (H) SEC analysis of a stoichiometric BRDT_BD1_:ClpC1_NTD_ mixture in the presence (solid line) or absence (dashed line) of BacPROTAC-3. The two proteins elute at the same volume also in the absence of BacPROTAC because of their similar size; however, BacPROTAC-3 addition mediates formation of an additional peak compatible with the elution volume expected for the BRDT_BD1_:BacPROTAC-3:ClpC1_NTD_ ternary complex. Coomassie stained SDS-PAGE gel of the collected peak fractions is shown. BRDT_BD1_ and ClpC1_NTD_ have identical electrophoretic mobility and are thus not distinguishable on the Coomassie stained gel. (I) Chemical structure of control compounds BacPROTAC-3a (where replacement of JQ1-(*S*) with JQ1-(*R*) disrupts BRDT_BD1_ binding), BacPROTAC-4a (where dCymM is replaced with the distomer with all the stereocenters inverted, compromising ClpC1 binding), BacPROTAC-5a (where replacement of JQ1-(*S*) with JQ1-(*R*) disrupts BRDT_BD1_ binding). (J) Chemical structure of desoxycyclomarin (dCym) (Weinhäupl et al., 2018) used in competition experiments reported in Figure 4H. (K) Representative ITC titration of ClpC1_NTD_ (200 μM loaded in the syringe) against BacPROTAC-4 (25 μM loaded in the cell); reported K_D_ value represents the average ± SD of three independent measurements. (L) Representative ITC titration of ClpC1_NTD_ (200 μM loaded in the syringe) against BacPROTAC-4a (25 μM loaded in the cell) showing no measurable binding.

**Figure 4:**
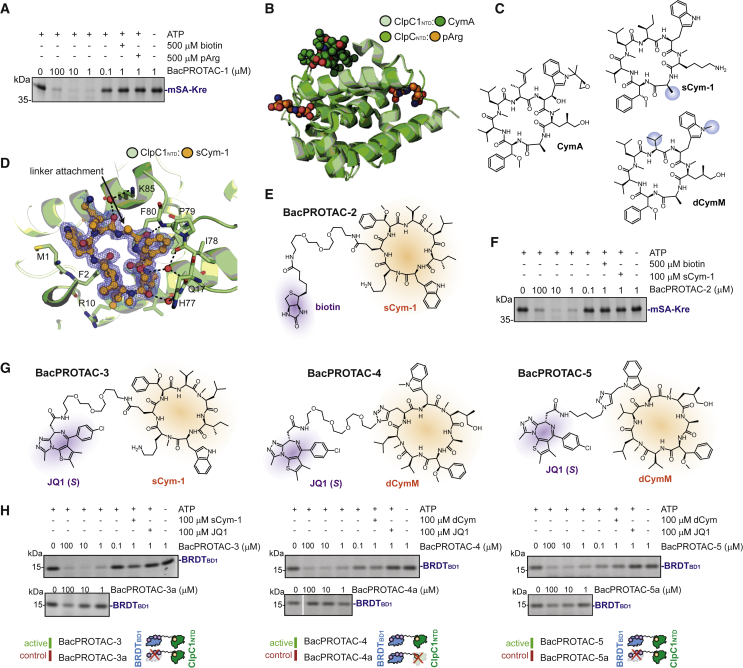
BacPROTACs can reprogram the mycobacterial ClpC1P1P2 (A) SDS-PAGE analysis of mSA-Kre degradation *in vitro*, after 2-h incubation with *M. smegmatis* ClpC1P1P2. The pArg-containing BacPROTAC-1 induces degradation in a concentration-dependent manner and can be outcompeted by separately provided pArg or biotin. (B) Superposition of ClpC_NTD_:pArg (PDB: 5HBN) with ClpC1_NTD_:CymA (PDB: 3WDC) crystal structure highlights the distinct locations of the ligand binding sites. (C) Chemical structure of CymA, sCym-1, and dCymM, chosen as alternative ClpC1-binding headgroups to replace the labile pArg moiety. Linker attachment points for BacPROTAC synthesis are highlighted with blue spheres. (D) sCym-1 co-crystal structure with ClpC1_NTD_ (PDB: 7AA4, this study), overlaid with the F_o_ − F_c_ electron density map of the ligand (calculated at 1.7 Å resolution, contoured at 2σ). The arrow indicates the position for attaching the BacPROTAC linker. The respective alanine side chain does not contribute to the sCym-1:ClpC1_NTD_ interface. (E) Chemical structure of BacPROTAC-2, designed to degrade mSA substrates employing sCym-1 as ClpC1 binding group. (F) *In vitro* degradation of mSA-Kre in the presence of BacPROTAC-2, using the same assay conditions as in (A). (G) Chemical structures of BRDT_BD1_-directed BacPROTACs: BacPROTAC-3 bridging JQ1 and sCym-1, BacPROTAC-4 and BacPROTAC-5 connecting JQ1 to dCymM through different linkers and attachment points. (H) SDS-PAGE analysis of *in vitro* degradation after 2-h incubation of BRDT_BD1_ with *M. smegmatis* ClpC1P1P2. Active BacPROTACs promote degradation of BRDT_BD1_ in a concentration-dependent manner and can be blocked by addition of individual head groups. BacPROTAC-3a and BacPROTAC-5a containing distomeric JQ1 as BRDT_BD1_-binding moieties induce partial degradation only at high concentrations. BacPROTAC-4a, containing a distomeric dCymM binding head does not induce significant BRDT_BD1_ degradation. Chemical structures of BacPROTAC-3a, -4a, -5a and dCym are shown in Figures S4I and S4J. Uncropped images of SDS-PAGE gels are shown in Data S1. See also Figure S4; Table S1.

The above biochemical and structural data revealed that pArg-based BacPROTACs can reprogram the ClpCP system of *B. subtilis*. To examine the therapeutic potential of the developed degrader, we aimed to transfer our approach to mycobacteria, which are among the most widely spread and dangerous human pathogens (WHO, 2021). Although a pArg-dependent degradation pathway has not been identified in mycobacteria, the substrate receptor of the ClpC1P1P2 protease has a bona fide and functional pArg receptor site, with all residues involved in phospho-guanidinium binding being fully conserved in its ClpC1_NTD_ domain (Weinhäupl et al., 2018). To test if pArg-based BacPROTACs can reprogram the mycobacterial degradation machinery, we analyzed the *Mycobacterium smegmatis* ClpC1 system. ITC and SEC experiments revealed that BacPROTAC-1 binds to ClpC1_NTD_ with high affinity (K_D_ = 0.69 μM) and promotes ternary complex formation with mSA and ClpC1_NTD_ (Figures S4A and S4B). Using the reconstituted ClpC1P1P2 protease, we observed that BacPROTAC-1 induces the degradation of a mSA substrate in a highly selective and efficient manner (Figure 4A). These data demonstrate that pArg-containing degraders can reprogram the ClpC1P1P2 protease of mycobacteria.

A major limitation in advancing pArg-based PROTACs is their poor pharmacokinetic profile and the chemical instability of the phospho-guanidinium group (Schmidt et al., 2014). To overcome these limitations, we looked for chemical entities that could replace pArg. Of note, the mycobacterial ClpC1 is targeted by a range of cyclic peptides that deregulate the ClpC1P1P2 complex (Lee and Suh, 2016). These antibiotics bind to the ClpC1_NTD_ substrate receptor domain and thus have a pArg-like targeting function. The best characterized ClpC1_NTD_-directed antibiotic is CymA (Vasudevan et al., 2013), which binds to a hydrophobic pocket located in a remote position relative to the pArg-binding sites (Figures 4B and 4C). The CymA binding pocket is highly conserved in ClpC1 unfoldases, but is absent in ClpC proteins from gram-positive bacteria, enabling selective targeting of the mycobacterial protease. Moreover, CymA can pass the cell envelope of mycobacteria (Schmitt et al., 2011) and thus represents a building block with important properties to explore the activity of small-molecule degraders in bacterial cells.

To prepare CymA-based degraders, we developed a solid-phase synthesis approach providing the 7-residue cyclic peptide in large quantities. To facilitate the *de novo* synthesis of the natural compound (Barbie and Kazmaier, 2016), we replaced certain non-proteinogenic amino acids with chemically simpler analogs. Guided by structural data (Vasudevan et al., 2013), we prepared a series of CymA-like cyclic peptides and identified sCym-1 as a high affinity ClpC1_NTD_ ligand (K_D_ = 0.81 μM; Figures 4C and S4C). A co-crystal structure obtained at 1.7 Å resolution (Figure 4D; Table S1) confirmed that the sCym-1 mimetic adopts the same binding mode as the native CymA antibiotic. Moreover, the crystal structure revealed a possible linker attachment site to synthesize BacPROTAC-2, in which sCym-1 is linked to biotin (Figure 4E). When tested in our biochemical assay, the sCym-1-based degrader stimulated removal of the mSA model substrate to similar extents as its pArg counterpart (Figures 4F, S4D, and S4E), indicating that derivatives of the CymA antibiotic can be repurposed as BacPROTAC components. Importantly, these data also show that various ClpC1_NTD_ binders can be exploited to develop chemical adaptors for targeted protein degradation. To further demonstrate that the system is generalizable, and since cellular biotin would compete for binding to mSA and impede BacPROTAC activity (Figure 4F), we looked for an alternative POI and identified the bromodomain-1 (BD1) of BRDT as attractive model substrate. BRDT_BD1_ (residues 21–137) encodes a soluble protein that binds with high affinity to BET bromodomain inhibitors (Matzuk et al., 2012). One of its small-molecule ligands, JQ1, has been widely used in various PROTACs (Winter et al., 2015; Zengerle et al., 2015), thus facilitating the rational design of bacterial degraders. Moreover, BRDT is a human protein with no bacterial homologues, allowing selective *in vivo* degradation without interfering with endogenous pathways. To target BRDT_BD1_, we synthesized BacPROTAC-3 linking sCym-1 and JQ1 (Figure 4G). The degrader was able to recruit BRDT_BD1_ (Figures S4F–S4H) and induce its degradation by ClpC1P1P2 in a highly specific manner (Figure 4H). Additionally, we synthesized BacPROTAC-3a incorporating the JQ1(*R*) enantiomer (Figure S4I) that displays a 60-fold lower affinity toward BRDT_BD1_ than the eutomer JQ1(*S*) (Filippakopoulos et al., 2010). Application of BacPROTAC-3a in biochemical assays showed strongly compromised degradation efficiency, consistent with its reduced binding affinity (Figure 4H).

Having shown that sCym-1 can be successfully incorporated into distinct bi-functional degraders, we extended the approach to a slightly modified derivative of the natural cyclomarin, dCymM (Figure 4C). Due to the technically demanding synthesis of dCymM (Kiefer et al., 2019), we had to use different attachment points than in sCym-1 (Figure 4C). We chose the N^1^-side chain on tryptophan and the adjacent valine unit of the cyclic dCymM peptide, as these building blocks are the last two added in the chemical synthesis, limiting adaptions of the previously established synthetic route. Moreover, it is known that modifications of these residues do not reduce the antibiotic properties of cyclomarin derivatives (Kiefer et al., 2019), suggesting that these side chains are not involved in binding to ClpC1 and thus represent suitable exit vectors. We further hypothesized that the replacement of the non-polar substituents with a rather lipophilic triazole-heterocycle should not significantly impair binding affinity to ClpC1. After attaching linker and JQ1 binding head, we obtained BacPROTAC-4 and BacPROTAC-5, respectively (Figure 4G). The dCymM containing degrader bound ClpC1_NTD_ with high affinity (K_D_ = 0.2 μM; Figure S4K) and induced BRDT_BD1_ degradation at lower concentrations than its sCym-1 analog (Figure 4H). Finally, we synthesized respective control compounds containing inactive enantiomers of the two headgroups (Figure S4I). Consistent with previous results with BacPROTAC-3a, BacPROTAC-5a with the distomeric JQ1(*R*) group induced partial degradation only at high concentrations, while BacPROTAC-4a, containing a distomeric dCymM binding head, did no longer bind to ClpC1_NTD_ (Figure S4L) and did not induce significant BRDT_BD1_ degradation (Figure 4H).

Together these data indicate that both sCym-1 and dCymM moieties can be incorporated into the chemical adaptor, tolerating distinct linker attachment points to yield potent and selective degraders. In conclusion, our data demonstrate that BacPROTACs represent a versatile molecular tool that can be applied to various protein substrates and allow integration of diverse head groups and chemical linkers.

### BacPROTACs induce protein degradation in mycobacteria

**Figure 5:**
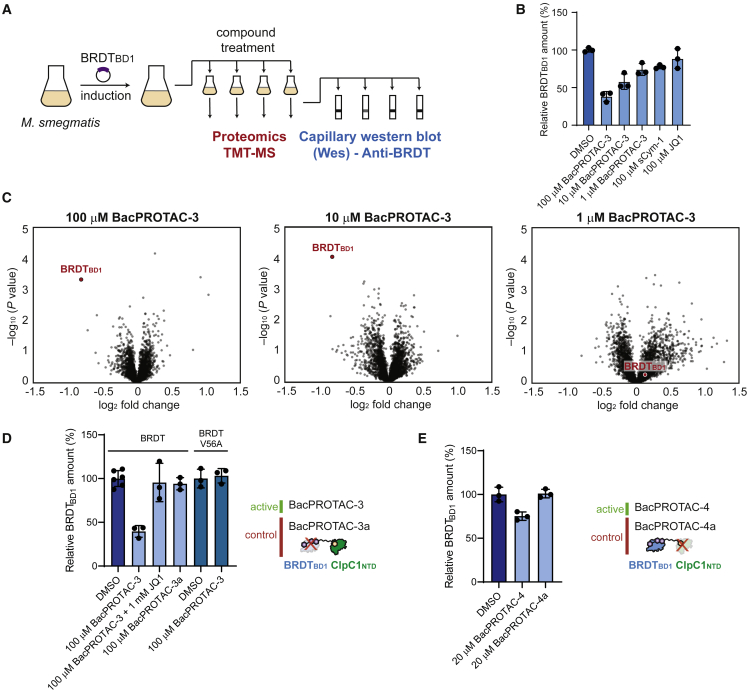
BacPROTACs mediate selective POI degradation in mycobacteria (A) Schematic outline of BRDT_BD1_ degradation analysis in mycobacteria. (B) Capillary western blot (Wes) analysis of BacPROTAC-3-mediated effects on BRDT_BD1_ after 30 min of incubation. BRDT_BD1_ levels relative to DMSO control treatment (dark blue) are plotted as mean ± SD of experimental triplicates. BacPROTAC-3 induces BRDT_BD1_ degradation in a concentration-dependent manner, while sCym-1 or JQ1 treatments did not significantly alter protein levels. (C) TMT-MS proteomics analysis of the BacPROTAC-3 effect after 2 h of incubation. The volcano plots show the fold-change (log_2_) in protein abundance in comparison with DMSO treatment, plotted against p value (−log_10_) (two-tailed Student’s t test; triplicate analysis). The BRDT_BD1_ protein is highlighted in red. (D) Wes analysis of BRDT_BD1_ levels after 2 h of incubation with the indicated compounds. DMSO was used as control. (E) Wes analysis of BRDT_BD1_ levels after 2 h of incubation with BacPROTAC-4 and BacPROTAC-4a, with the latter compound carrying a binding-deficient dCymM distomer. All Wes data are represented as mean ± SD. See also Figure S5; Table S4; Data S1.

After validating the activity of the Cym-based BacPROTACs *in vitro*, we investigated whether they were able to reprogram ClpC1P1P2 in a cellular environment. For this purpose, we used *M. smegmatis* cells stably expressing BRDT_BD1_ to perform proteomics and degradation experiments (Figure 5A). We treated the culture with BacPROTAC-3 or alternatively the individual building blocks sCym-1 and JQ1. After 30-min incubation, we quantified BRDT_BD1_ levels using capillary western blots. We found that BacPROTAC-3 induced BRDT_BD1_ degradation in a concentration-dependent manner, while sCym-1 or JQ1 treatments did not significantly alter BRDT_BD1_ levels (Figure 5B).

Given the reported deregulation of ClpC1P1P2 by CymA (Maurer et al., 2019), we next assessed whether BacPROTAC-3 led to selective elimination of BRDT_BD1_ or had a global impact on the mycobacterial proteome. To address this point, we performed a tandem mass tag mass spectrometric (TMT-MS) analysis of *M. smegmatis* lysates. Isobaric labeling allowed the detection and quantification of 2,912 proteins. Among these, only BRDT_BD1_ was significantly (p < 0.001) depleted upon BacPROTAC-3 treatment (Figure 5C). Thus, the quantitative TMT-MS analysis provides compelling evidence that BacPROTAC-3 is capable of inducing degradation of the BRDT_BD1_ substrate in a highly specific manner.

**Figure S5:**
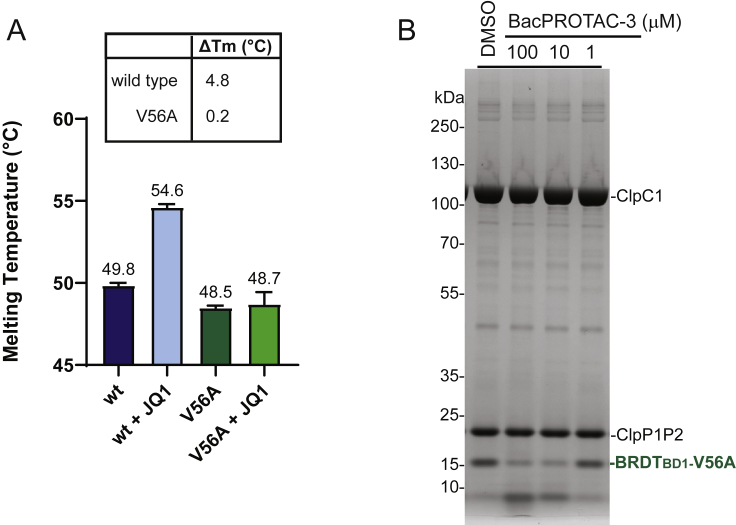
Characterization of a BRDT_BD1_ mutant with compromised JQ1 binding, related to Figure 5 (A) Characterization of BRDT_BD1-V56A_ by DSF. Melting temperatures (T_m_, °C) derived from triplicate measurements are plotted as mean ± SD. ΔT_m_ (°C) was calculated as T_m_(BRDT_BD1_ + JQ1) − T_m_(BRDT_BD1_). The engineered mutant shows a similar T_m_ as the wild-type protein but is not significantly stabilized by JQ1. (B) SDS-PAGE analysis of *in vitro* degradation assay. Incubation of BRDT_BD1-V56A_ with ClpC1P1P2 and different concentrations of BacPROTAC-3 (2-h incubation, DMSO used as control) shows compromised degradation efficiency compared with wild-type BRDT_BD1_ (Figure 4H).

To show that BRDT_BD1_ degradation results from direct engagement via BacPROTAC-3, we tested the inhibitory effect of JQ1 in mycobacteria. Upon co-incubation with JQ1, the induced BRDT_BD1_ degradation was strongly inhibited (Figure 5D), likely because the applied headgroup competes with the BacPROTAC for POI binding thus preventing its recruitment by the Clp protease. Furthermore, when treating the bacterial cultures with BacPROTAC-3a, containing the JQ1(*R*) distomer, BRDT_BD1_ levels remained unchanged (Figure 5D). To further confirm these data, we designed a BRDT_BD1-V56A_ mutant that was deficient in binding JQ1 but otherwise exhibited wild-type properties (Figure S5A). The introduced point mutation strongly reduced BacPROTAC-mediated degradation by the reconstituted Clp protease (Figure S5B). Likewise, we could not observe a reduction in BRDT_BD1-V56A_ levels at the highest tested BacPROTAC-3 concentration *in vivo*, reflecting the impaired targeting of the mutant protein (Figure 5D). Accordingly, degradation by ClpC1P1P2 requires the direct engagement of BRDT_BD1_ through the JQ1 moiety of BacPROTACs. To confirm that the Cym head specifically targets ClpC1 inside mycobacteria, we tested BacPROTAC-4a containing the dCymM distomer. In contrast to the parent degrader, treatment of bacteria with BacPROTAC-4a did not lower BRDT_BD1_ levels in the cell (Figure 5E). Taken together, these *in vivo* data demonstrate that Cym-based BacPROTACs can reprogram ClpC1P1P2 to induce POI degradation in mycobacteria. Furthermore, our data show that despite its chemical modification, the cyclomarin scaffold maintains its ability to pass the mycobacterial cell envelope, providing an attractive tool for BacPROTAC development.

### Induced degradation of target proteins in mycobacteria

**Figure 6:**
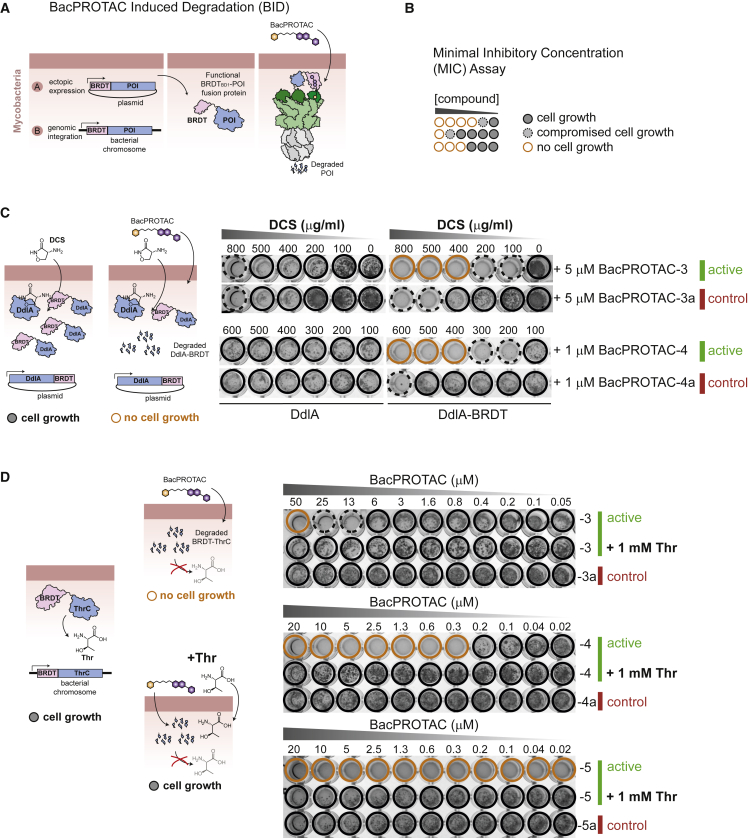
BacPROTAC-induced degradation (BID) approach in mycobacteria (A) Schematic representation of the BID approach. Proteins of interest are fused to BRDT_BD1_ and either introduced (A) on a plasmid or (B) on the chromosomal locus, leading to expression of POI-BRDT_BD1_ fusion proteins in mycobacteria. Selective POI-BRDT_BD1_ degradation can be triggered by addition of a BRDT_BD1_ targeting BacPROTAC. (B) Schematic of the applied MIC assay, introducing color code to illustrate sensitivity of *M. smegmatis* cells to indicated compounds (normal growth, black circle; compromised growth, black dotted circle; no growth, orange circle). (C) Representative MIC assay showing reduced sensitivity of *M. smegmatis* to D-cycloserine (DCS) upon expression of DdlA or DdlA-BRDT_BD1_. Addition of active BacPROTAC-3 and -4 restored DCS sensitivity, whereas BacPROTAC-3a and BacPROTAC-4a, containing either the binding-deficient JQ1(*R*) or dCymM distomer, could not evoke this phenotype. Expression of wild-type DdlA was used as control for potential side effects of BacPROTAC treatment; however, cell growth was not affected at the applied conditions. (D) MIC assay showing BacPROTAC-dependent L-threonine auxotrophy of the *thrC::BRDT-thrC M. smegmatis* strain, carrying a chromosomally encoded BRDT_BD1_-ThrC fusion. Only in the presence of active BacPROTAC, an auxotrophic phenotype is observed that is fully rescued by the addition of 1 mM L-threonine (Thr). Included controls with BacPROTAC-3a and 5a (JQ1(*R*) distomer) and BacPROTAC-4a (dCymM distomer) exclude that the phenotype is due to pleotropic effects of one of the two head groups. See also Figure S6. Uncropped images of MIC plates are shown in Data S1.

As BacPROTACs can induce the *in vivo* degradation of BRDT_BD1_ in a highly specific manner, we explored whether we could repurpose the JQ1/BRDT_BD1_ degron for conditional protein depletion in mycobacteria. BacPROTAC-induced protein degradation, to which we refer as BacPROTAC-induced degradation (BID) (Figure 6A), would represent a bacterial equivalent to eukaryotic systems of inducible protein degradation.

**Figure S6:**
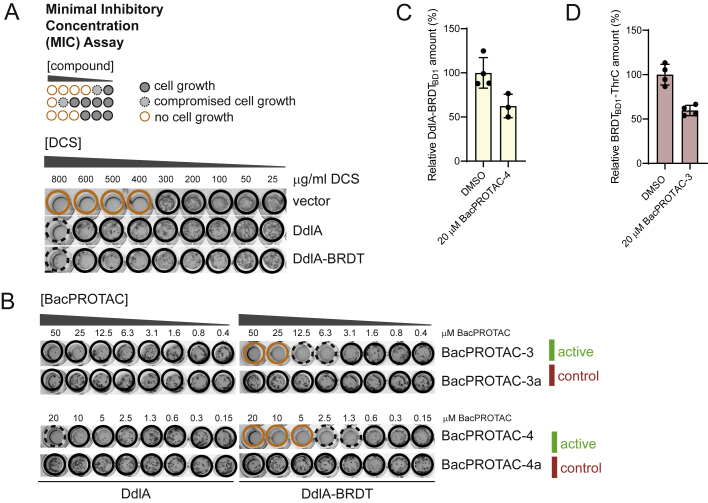
BacPROTAC-induced degradation (BID) approach in mycobacteria, related to Figure 6 (A and B) Minimal inhibitory concentration (MIC) assays. Individual wells are color coded to highlight where cells show full growth (black circle), compromised growth (black dotted circle), and no growth (orange circle). (A) Overexpression of DdlA and DdlA-BRDT desensitizes *M. smegmatis* against DCS. Vector control cells carry the empty pMyC vector. (B) MIC assay showing BacPROTAC-dependent inhibition of cell growth of *M. smegmatis* expressing DdlA-BRDT (BacPROTAC-3 and -4). However, growth was not impaired when DdlA alone was expressed. The included controls with inactive JQ1 and dCymM head groups (BacPROTAC-3a and 4a) highlight the specificity of the BacPROTAC-induced degradation (BID). (C and D) Capillary western blot (Wes) analysis of BacPROTAC-mediated effects on DdlA-BRDT_BD1_ (C) and BRDT_BD1_-ThrC (D) in the cell after 24 h of incubation, showing significant degradation of the targeted fusion proteins compared with DMSO treatments. BRDT_BD1_ levels are shown relative to DMSO treatment are plotted as mean ± SD. Uncropped images of MIC plates are shown in Data S1.

First, we tested the degradation of an ectopically expressed protein that is associated with a specific biological phenotype. As model protein, we selected D-alanylalanine synthase (DdlA), an essential component of the peptidoglycan synthesis pathway, which is also the major target of the broad-spectrum antibiotic D-cycloserine (DCS) (Feng and Barletta, 2003). Moreover, it is known that overexpression of DdlA desensitizes *M. smegmatis* against DCS (Feng and Barletta, 2003). Similar to the wild-type protein, ectopic expression of the DdlA-BRDT_BD1_ fusion protein rendered *M. smegmatis* less susceptible to DCS, as reflected by the increased minimal inhibitory concentration (MIC) of DCS to inhibit growth (Figure S6A). These data indicate that the expressed DdlA-BRDT_BD1_ is fully functional *in vivo*. Importantly, addition of active BacPROTACs fully restored DCS sensitivity, whereas control compounds with distomeric JQ1 or cyclomarin head groups did not show this effect (Figures 6B and 6C). Consistent with the induced drug sensitivity, quantification of cellular DdlA-BRDT_BD1_ illustrated the decreased protein levels upon BacPROTAC treatment (Figure S6C). Together, the *in vivo* data demonstrate that the synthetic degraders induce removal of DdlA-BRDT_BD1_, making cells more susceptible against DCS. Notably, DCS sensitivity was decreased even further than in cells expressing a vector control (Figures 6C and S6A) pointing to an additive activity of BacPROTAC and DCS. Since DdlA exists as dimeric protein (Bruning et al., 2011), the DdlA-BRDT_BD1_ fusion protein might associate with endogenous DdlA, co-targeting it to the ClpC1P1P2 protease. Consistent with this model, we found that cells treated with BacPROTAC were compromised in growth only when DdlA-BRDT_BD1_—but not DdlA alone—was ectopically expressed (Figure S6B). The observed *in vivo* effects thus demonstrate the potential of the BID technology in exploring drug targets and antimicrobial combination therapies.

To illustrate the versatility of the JQ1/BRDT_BD1_ degron as research tool to eliminate a cellular protein, we aimed at evoking an auxotrophic phenotype in mycobacteria. As model substrate, we used the threonine synthetase ThrC, an essential enzyme catalyzing the last step in threonine biosynthesis (Covarrubias et al., 2008). After showing efficient degradation of ectopically expressed BRDT_BD1_-ThrC in *M. smegmatis* by BacPROTAC-3 (Figure S6D), we integrated the BRDT_BD1_ coding region in front of the start codon of the *thrC* gene (*M. smegmatis thrC::BRDT*_*BD1*_*-thrC*), allowing expression of the fusion protein under transcriptional control of the chromosomal locus. Expression of BRDT_BD1_-ThrC did not interfere with cell growth, as the mutant strain showed similar growth characteristics as the wild-type strain. Strikingly, addition of active BacPROTACs induced a strong auxotrophic phenotype, which could be rescued by addition of L-threonine (Figure 6D). In contrast, BacPROTACs containing inactive head groups did not inhibit cell growth. Remarkably, the functional BacPROTAC-4 and 5 exhibited ∼15-fold different efficiencies in inducing auxotrophy, even though they carry the same head groups. As both compounds stimulate degradation *in vitro* to similar extents (Figure 4H), the observed *in vivo* difference seems to be related to distinct cell uptake properties caused by different linkers and linker attachment points on dCymM (Figures 4C and 4G). Moreover, it should be noted that BacPROTAC-3a and -5a combining the JQ1(*R*) distomer and the active sCym-1 and dCymM head groups did not induce any growth defect (Figures 6C and 6D). These data indicate that binding of the cylomarin headgroup to ClpC1 does not cause pleiotropic effects under the applied conditions. As for DdlA-BRDT, the selective removal of the ThrC fusion protein further highlights the potential of the BacPROTAC technology as research tool, allowing induced degradation of target proteins from various cellular pathways.

## Discussion

In the present work, we developed small-molecule adaptors, BacPROTACs, that expand the targeted protein degradation technology to bacteria. The bi-functional adaptors redirect the ClpCP protease, the functional equivalent of the eukaryotic proteasome, to target neo-substrates in a highly specific manner. As seen for pArg and cyclomarin head groups, various molecules that bind to the substrate receptor of the ClpCP protease can be incorporated into a functional degrader. Using cell permeable BacPROTACs, we furthermore demonstrate that recruitment of model proteins to ClpCP leads to selective protein degradation in bacterial cells. A critical next step will be the targeting and induced elimination of an essential bacterial protein. As a first step in this direction, we show that targeted degradation of mycobacterial proteins fused to an enabled degron elicits bacteriotoxicity. Given the advantages of bi-functional degraders, such as their modular design and possibility to repurpose diverse protein ligands, BacPROTACs could open new horizons to develop antimicrobials with high selectivity and species specificity. Noteworthy, the developed approach is broadly applicable to bacterial proteins, since host-pathogen selectivity is achieved by reprogramming the ClpCP protease absent in mammalian cells. Considering the similar structural organization of bacterial ClpCP, ClpAP, and ClpXP proteases, which all employ the N-terminal domains of their ATPase units as substrate receptor, we propose that the BacPROTAC approach can be even expanded to related Clp proteolytic complexes and eventually used as universal method to reprogram bacterial proteases.

The possibility of eliminating a POI from the cell has broad applications for basic research as well. The function of proteins in prokaryotic and eukaryotic cells is often studied by perturbation at the level of transcription or mRNA stability. In contrast to eukaryotes, the knockdown of prokaryotic proteins is often complicated by polycistronic gene arrangements, where interference with transcription of one gene often affects all downstream located open reading frames. Specific genetic manipulations can therefore be tedious and difficult, especially when dealing with essential proteins. Special strategies that allow conditional, reversible, and temporal control of protein levels are required. Conditional interference with gene transcription by CRISPRi techniques has been established as a method of choice in prokaryotes (Mougiakos et al., 2018). However, this approach also has limitations, related to the co-repression of genes in polycistronic operons and leading to higher sensitivity to toxic compounds as well as to longer generation times (Rock et al., 2017). Chemical biology approaches that leverage the potency of small-molecule degraders could help to overcome these limitations. Prominent examples in eukaryotes are the auxin-induced degradation (Nishimura et al., 2009), the FKBP12-degron based dTAG system (Nabet et al., 2018) as well as the selective removal of HaloTag fusion proteins (Neklesa et al., 2011). By combining chemical biology with biochemistry and microbiology, we established an analogous prokaryotic system that enables selective degradation of endogenous proteins by the ClpC1P1P2 protease. This so-called BID system allows to carry out conditional knockdown experiments in mycobacteria. The system is based on fusing a target protein to the BRDT_BD1_ degron, which is a small bromodomain of 16 kDa that is stable in mycobacteria. BacPROTAC treatment leads to the selective degradation of the POI-BRDT_BD1_ fusion protein. As we have shown for the model proteins DdlA and ThrC, the BID method allows to characterize the function of essential bacterial proteins and explore their potential as antimicrobial targets. The method is also flexible in its application. It proved to be effective *in vivo* at different cytosolic POI concentrations, depending on whether expression of fusion proteins was governed from an inducible or endogenous promoter. Moreover, the BRDT_BD1_ degron was successfully added on both termini of the POI. Potential challenges such as impaired complex formation after degron fusion can be addressed by appropriate tag placement and linker design. However, it should be noted that not all fusion proteins yield efficient ClpCP substrates, as structural features of introduced POIs may affect degradability. It will thus be important to further test what properties make a favorable BacPROTAC substrate and eventually a promising antibiotic target. Importantly, our *in vivo* data show that the conditional proteolytic depletion of essential proteins can perturb cell growth up to the level of exhibiting antimicrobial activity. Using induced bacterial lethality as readout, systematic BID screens can be carried out to identify essential proteins in mycobacteria, such as ThrC and DdlA, that are amenable to the BacPROTAC technology and represent attractive targets for developing antibacterial compounds.

### Limitations of the study

A limitation of the study lies in the apparent *in vivo* degradation efficiency that is less pronounced compared with our biochemical data. This discrepancy is likely due to the incomplete cell permeability of BacPROTACs, or adsorption by the mycobacterial extracellular matrix, lowering their effective intracellular concentration. Of note, the mycobacterial cell wall is densely decorated with lipids, yielding a waxy protection shield, not present in eukaryotic cells, that diminishes access of many chemicals. Though measuring intracellular concentrations of tested compounds could clarify this point, standard drug uptake assays are especially ambiguous for mycobacteria. Furthermore, *in vivo* degradation needs to be monitored at high cellular density if compound amount is limited. As bacterial cultures enter stationary phase at such condition, proteolytic activities are expected to be reduced. In contrast, the phenotypic readout of BacPROTAC activity in plate assays revealed them to be highly potent, and the *in vivo* efficacy is comparable to *in vitro* degradation assays.

Another note of caution relates to the cyclomarin group incorporated into BacPROTACs. By deregulating the ClpC1P1P2 protease, Cym-based degraders may interfere with cellular levels of unrelated proteins. Such indirect effects require controls when interpreting BID data, for example, by using BacPROTACs with binding-deficient head groups. In contrast, mycobacterial toxicity could enhance the antimicrobial potential of BacPROTACs. Compounds that target and reprogram ClpC1 would have a dual effect, disturbing the mycobacterial proteome and, in addition, targeting an essential POI for degradation. However, *in vivo* degradation is so far limited to the BRDT_BD1_/JQ1 degron and respective fusion proteins. In order to take full advantage of small-molecule degraders as antibiotics, it will be now crucial to prove that endogenous bacterial proteins can be degraded in the same fashion.

## STAR★Methods

### Key resources table

**Table:** 

REAGENT or RESOURCE	SOURCE	IDENTIFIER
**Antibodies**		

Anti-human BRDT	BioVision	Cat#6643
Anti *E. coli* RpoB [8RB13]	BioLegend	Cat#663905

**Bacterial and virus strains**		

*M. smegmatis* mc^2^-155	ATCC	Cat#700084
*M. smegmatis thrC::BRDT*_*BD1*_*-thrC*	This paper	N/A

**Chemicals, peptides, and recombinant proteins**		

*B. subtilis* ClpC with his-tag	(Trentini et al., 2016)	N/A
*B. subtilis* ClpC_DWB_ (E280A/E618A) with his-tag	(Trentini et al., 2016)	N/A
*B. subtilis* ClpC_NTD_ (1–148) with his-tag	(Trentini et al., 2016)	N/A
*B. subtilis* ClpP with his-tag	(Trentini et al., 2016)	N/A
*M. smegmatis* ClpC1	This paper	N/A
*M. smegmatis* ClpC1 NTD (1–148)	This paper	N/A
*M. smegmatis* ClpP1 with his-tag	This paper	N/A
*M. smegmatis* ClpP2 with his-tag	This paper	N/A
Human BRDT_BD1_ with his-tag	(Filippakopoulos et al., 2012)	N/A
Human BRDT_BD1-V56A_ with his-tag	This paper	N/A
DdlA	This paper	N/A
DdlA-BRDT_BD1_	This paper	N/A
BRDT_BD1_-ThrC	This paper	N/A
mSA-NrdI	This paper	N/A
mSA-TagD	This paper	N/A
mSA-NusA	This paper	N/A
mSA-Kre	This paper	N/A
pArg-β-casein	(Trentini et al., 2016)	N/A
pyruvate kinase	Sigma-Aldrich	Cat#9136-5KU
sCym-1	This paper	N/A
dCymM	(Kiefer et al., 2019)	N/A
BacPROTAC-1	This paper	N/A
BacPROTAC-1a	This paper	N/A
BacPROTAC-1b	This paper	N/A
BacPROTAC-1c	This paper	N/A
BacPROTAC-2	This paper	N/A
BacPROTAC-3	This paper	N/A
BacPROTAC-3a	This paper	N/A
BacPROTAC-4	This paper	N/A
BacPROTAC-4a	This paper	N/A
BacPROTAC-5	This paper	N/A
BacPROTAC-5a	This paper	N/A
phosphoenolpyruvatePhosphoenolpyruvate	Sigma-Aldrich	Cat#860077-1G
Trifluoroacetic Acid	Fisher Scientific	Cat# 11378277
14-Azido-3,6,9,12-tetraoxatetradecan-1-amine	Abcr	Cat# AB525702
HATU	Carbolution Chemicals	Cat# CC01011
N,N-diisopropylethylamine	Sigma-Aldrich	Cat# D125806
Copper(II) Sulfate	Fisher Scientific	Cat# 10246780
Sodium Ascorbate	Fisher Scientific	Cat# 1433358
5-azidopentan-1-amine	Enamine	Cat# EN300-218785
D-Cycloserine	Carl Roth	CN37.1
JQ1	Sigma-Aldrich	SML1524-5MG
7H9	Sigma-Aldrich	M0178-500G
7H10	Sigma-Aldrich	M0303-500G
Tween 80	Sigma-Aldrich	P1754-500ML

**Critical Commercial Assays**		

12–230 kDa Wes Separation Module	ProteinSimple	Cat#SM-W004
Anti-Rabbit Detection Module	ProteinSimple	Cat#DM-001
Anti-Mouse Detection Module	ProteinSimple	Cat#DM-002

**Deposited Data**		

ClpC_NTD_:sCym-1 Crystal Structure	This paper and Protein Data Bank	7AA4
ClpC Hexamer Cryo-EM Model	This paper and Protein Data Bank	7ABR
Cryo-EM Map of ClpC Hexamer	This paper and EM DataResource	EMD-11707
Cryo-EM Map of ClpC Tetramer-of-Hexamers	This paper and EM DataResource	EMD-11708
Cryo-EM Raw Micrographs	This paper and Electron Microscopy Public Image Archive	EMPIAR-847
MS-Proteomics Data	This paper and PRoteomics IDentification Database	PRIDE PXD021505

**Oligonucleotides**		

For Primer Sequences See Table S3	This paper	N/A

**Recombinant DNA**		

pETM14 Plasmid	Genewiz	https://www.genewiz.com/
pNIC28-Bsa4 plasmid	(Savitsky et al., 2010)	addgene Cat#26103
pMyC plasmid	(Beckham et al., 2020)	addgene Cat#42192
p2NIL plasmid	(Parish and Stoker, 2000)	addgene Cat#20188
pGOAL-19 plasmid	(Parish and Stoker, 2000)	addgene Cat#20190

**Software and algorithms**		

MicroCal PEAQ-ITC Analysis Software	Malvern	N/A
AlphaFold	(Jumper et al., 2021), (Varadi et al., 2022)	N/A
DSF data analysis	(Niesen et al., 2007)	ftp://ftp.sgc.ox.ac.uk/pub/biophysics
cryoSPARC v2	(Punjani et al., 2017)	https://cryosparc.com/
MotionCor2 1.0.5	(Zheng et al., 2017)	https://emcore.ucsf.edu/ucsf-software
Gctf 1.06	(Zhang, 2016)	N/A
crYOLO v1.3.5	(Wagner et al., 2019)	http://sphire.mpg.de
RELION 3.0	(Zivanov et al., 2018)	N/A
Coot	(Emsley et al., 2010)	https://www2.mrc-lmb.cam.ac.uk/personal/pemsley/coot/
Phenix	(Afonine et al., 2018), (Liebschner et al., 2019)	https://phenix-online.org/
MolProbity	(Williams et al., 2018)	http://molprobity.biochem.duke.edu/
EMRinger	(Barad et al., 2015)	N/A
UCSF Chimera	(Goddard et al., 2007)	https://www.cgl.ucsf.edu/chimera/
UCSF Chimera-X	(Goddard et al., 2018)	https://www.cgl.ucsf.edu/chimerax/
PyMol	(Schrodinger, 2015)	https://pymol.org/2/
XDS-package	(Kabsch, 2010)	https://xds.mr.mpg.de/
Compass for SW software	ProteinScience	https://www.proteinsimple.com/
Proteome Discoverer 2.3	Thermo Fisher Scientific	N/A
MS Amanda	(Dorfer et al., 2014)	https://ms.imp.ac.at/
Percolator	(Kall et al., 2007)	N/A
IMP-Hyperplex	N/A	https://ms.imp.ac.at/
RStudio	RStudio	https://www.rstudio.com/
Prism	GraphPad	https://www.graphpad.com/scientific-software/prism/

**Other**		

Cu/Pd Hexagonal 400 mesh grids	Agar Scientific	Cat#AGG2440PD
R2/2 Cu 200 mesh grids	Jena Bioscience	Cat#X-103-Cu200
Nunc™ MicroWell™ 96-Well, Nunclon Delta-Treated, Flat-Bottom Microplate	Fisher Scientific	Cat#167008
U-shaped 96-well glass-coated microplates	Fisher Scientific	Cat#60180-P300
flat 96-well plates Thermo Nunc Microwell	Sigma-Aldrich	Cat#P8366-50EA
U-shaped 96-well glass-coated microplates	Fisher Scientific	Cat#60180-P306
Sera-Mag SpeedBeads, variant 1	cytiva	Cat#45152105050250
Sera-Mag SpeedBeads, variant 2	cytiva	Cat#65152105050250
TMTpro™ 16plex	Thermo Fisher Scientific	Cat#A44520
PepSwift Monolithic RSLC column	Thermo Fisher Scientific	Cat#164542
XBridge Peptide BEH C18 Column	Waters	Cat#186003613
Acclaim PepMap C-18 precolumn	Thermo Fisher Scientific	Cat#160454
Acclaim PepMap C-18 column	Thermo Fisher Scientific	Cat#164942
Eclipse XDB-C18 (5 μm)	Agilent	Cat# 990967-902
*Büchi Flashpure Select C18* cartridges	Büchi	Cat# 140000121
Onyx monolithic C18 column	Phenomenex	Cat# CH0-7644
Luna 3 μm C18(2) column	Phenomenex	Cat# 00B-4251-E0

### Resource availability

#### Lead contact

Further information and requests for resources and reagents should be directed to and will be fulfilled by the lead contact, Tim Clausen (tim.clausen@imp.ac.at).

#### Materials availability

All unique materials and reagents generated in this study are available from the Lead contact with a completed material transfer agreement.

There are restrictions to the availability of the generated BacPROTAC probes and simplified cyclomarin analogues generated in this study due to a limited stock. Reasonable aliquots of the compounds are available until stocks run out from the Lead Contact with a completed Material Transfer Agreement.

### Experimental model and subject details

#### Cultivation of *M. smegmatis*

*M. smegmatis* mc^2^-155 (ATCC 7000084) was directly purchased from ATCC and freshly inoculated from glycerol stocks. *M. smegmatis thrC::BRDT*_*BD1*_*-thrC* was obtained as a single cross-over from *M. smegmatis* mc^2^-155 (ATCC 7000084) as described below and apart from the site of integration can be considered isogenic to the parental strain.

Liquid cultures of *M. smegmatis* were grown in 7H9 medium (Sigma) supplemented with 0.2% (v/v) glycerol and 0.025% (v/v) Tween80 (Sigma). *M. smegmatis* transformed with the respective pMyC-POI vectors were additionally supplemented with 50 μg/ml Hygromycin. All liquid cultures were grown under constant agitation (200 rpm) at 37°C. If applicable, ectopic expression was induced with 0.1% (w/v) acetamide. Cells were harvested by centrifugation (3,000 x g, 5 min, 25 °C) and processed according to the respective experiment. Solid cultivation (agar-based assays as well as plating steps) was performed with 7H10 agar supplemented with 0.5% (v/v) glycerol. MIC assay plates for the POI DdlA were supplemented with 0.1% (w/v) acetamide and 50 or 100 μg/ml Hygromycin. Cells were plated out of the exponential growth phase and all plates were incubated for 2.5-5 days at 37 °C.

### Method details

#### DNA constructs expression in *E. coli*

Cloning of constructs for *Escherichia coli* overexpression of full-length *B. subtilis* ClpC, ClpC_DWB_ (E280A/E618A), ClpC_NTD_ (1–148), ClpP, and BRDT_BD1_ was previously described (Filippakopoulos et al., 2012; Trentini et al., 2016). BRDT_BD1-V56A_ was obtained by site-directed mutagenesis of BRDT_BD1._ In case of expression constructs of *B. subtilis* ClpC, ClpC_DWB_, ClpC_NTD_ and ClpP, all variants were fusion constructs containing a C-terminal hexahistidine tag, except that for BRDT_BD1_ which contained a N-terminal hexahistidine tag followed by a TEV cleavage site.

Synthetic DNA of *M. smegmatis clpP1*, *clpP2* and *clpC1* genes (MSMEG_4673, MSMEG_4672, MSMEG_6091) was ordered from GeneArt (Thermo Fisher) and cloned into a pET21a vector DNA. ClpP1 and ClpP2 expression constructs encode a C-terminal tetrahistidine tag fusion, while that of ClpC1 expresses wild type protein. The coding region of *M. smegmatis clpC1* NTD (1–148) was cloned into pET21a and encodes a C-terminal hexahistidine tag fusion.

DNA for mSA-fusion constructs of three *B. subtilis* proteins (mSA-NrdI, mSA-TagD, mSA-NusA) cloned into a pETM14 vector was purchased from Genewiz. Expression constructs of mSA and mSA-Kre were cloned into a pNIC28-Bsa4 vector. These constructs encode a N-terminal hexahistidine tag followed by a TEV cleavage site fusion and a glycine-serine linker introduced between mSA and the bacterial protein. The mSA-Kre construct used for cryo-EM analysis was cloned into pET21a vector DNA and encodes a C-terminal hexahistidine tag fusion. All ORFs were verified for their correct sequence by DNA sequencing. Table S2 reports the translated amino acid sequences of all mSA-fusion proteins.

#### Protein expression and purification

Plasmid DNA was transformed into *E. coli* BL21 (DE3) or Rosetta cells (for *M. smegmatis* ClpC1, ClpP1 and ClpP2 proteins) and grown in LB broth supplemented with the respective antibiotic at 37 °C. Protein expression was induced by adding 0.1-0.5 mM isopropyl-1-thio-β-D-galactopyranoside (IPTG) at an OD_600_ of 0.8. Cells were cultured overnight at 18-20 °C, harvested by centrifugation and either lysed by sonication in a buffer containing 500 mM NaCl, 50 mM Tris pH 7.5, 10 mM imidazole, and 0.25 mM tris(carboxyethyl)phosphine (TCEP), or flash frozen and stored at -80 °C until purification.

Cell debris was removed by centrifugation. His-tagged proteins were purified from the cleared supernatants using Ni-NTA Agarose beads in batch. After several washing steps, bound protein was eluted using 50 mM Tris pH 7.5, 100 mM NaCl, 300 mM imidazole, 0.25 mM TCEP. Eluted protein was loaded onto a size exclusion chromatography column (Superdex 75 16/60 or Superdex 200 16/60 (GE Healthcare) depending on protein size) equilibrated in 50 mM Tris pH 7.5, 100 mM NaCl. For ClpC and ClpC_DWB_ purifications, the salt concentration of the size exclusion buffer was increased to 300 mM NaCl. Purified fractions were pooled and concentrated before flash freezing and stored at −80 °C.

Cell pellets for ClpP1 and ClpP2 purification were resuspended in 50 mM HEPES-NaOH pH 7.8, 300 mM NaCl, 30 mM imidazole and lysed by sonication. Cleared lysates were purified by Ni-NTA affinity chromatography (elution buffer: 50 mM HEPES-NaOH, pH 7.8, 300 mM NaCl, 250 mM imidazole) and subsequently by SEC using a Superose 6 16/60 column (GE Healthcare) equilibrated with 50 mM HEPES-NaOH pH 7.2, 150 mM KCl. 10% (v/v) glycerol was added to the elution fractions before flash freezing and storage at -80 °C. Processing of the full-length ClpP1 and ClpP2 to the mature ClpP1P2 complex was performed similar as previously described by Leodolter et al. (Leodolter et al., 2015).

Cell pellets for ClpC1 purification were resuspended in 50 mM Tris pH 7.5, 75 mM KCl, 2 mM EDTA, 10% (v/v) glycerol and lysed by sonication. After clarification of the lysate, ClpC1 was precipitated using 40% (w/v) ammonium sulphate. The pellet was resuspended in lysis buffer, loaded on a HiLoad 26/10 Q Sepharose High Performance column (GE Healthcare) equilibrated with lysis buffer and eluted in a gradient to 1 M KCl. ClpC1-containing fractions were pooled and precipitated again using 40% (w/v) ammonium sulphate. The pellet was resuspended in 50 mM HEPES-NaOH pH 7.2, 150 mM KCl, 10% (v/v) glycerol and loaded on a HiLoad 16/10 Superdex 200 prep grade SEC column (GE Healthcare) equilibrated in the same buffer. ClpC1 containing fractions were pooled and stored at -80 °C. Protein purity was monitored by Coomassie stained SDS-PAGE and correct molecular mass of purified proteins was verified by mass spectrometry.

#### Isothermal Titration Calorimetry (ITC)

ITC experiments were performed using a MicroCal PEAQ-ITC instrument (Malvern) at 25 °C in a buffer containing 50 mM Tris pH 7.5, 100 mM NaCl. Each titration consisted of 19 injections with intervals of 120 s (the first injection of 0.4 μL was followed by 18 injections of 2 μL) at constant stirring at 750 rpm. DMSO concentration was matched between cell and syringe to be 2% (v/v). Data were fitted using a single binding site model with a fitted offset subtraction using the MicroCal PEAQ-ITC Analysis Software. Each titration was repeated at least twice.

Titrations involving BacPROTAC-1 were performed with the ligand loaded into the syringe and the protein into the cell. 400 μM BacPROTAC-1 was titrated against 20 μM ClpC_NTD_ or mSA. A control titration of ligand into buffer was performed in order to determine the heat of dilution.

ITC experiments with BacPROTAC-4 and BacPROTAC-4a were performed in a buffer containing 50 mM Tris pH 7.5, 300 mM NaCl, 0.5 mM TCEP. Each titration consisted of 19 injections with intervals of 150 s (the first injection of 0.4 μL was followed by 18 injections of 2 μL) at constant stirring at 750 rpm. DMSO concentration was matched between cell and syringe to be 4% (v/v).

Titrations involving sCym-1, BacPROTAC-2, BacPROTAC-3, BacPROTAC-4 and BacPROTAC-4a were performed with the ligand loaded into the cell and protein into the syringe to account for the low solubility of the compounds in aqueous solutions.

#### Differential Scanning Fluorimetry (DSF)

DSF experiments were carried out using a CFX96 real-time PCR detection system (Bio-Rad). 5 μM BRDT_BD1_ or BRDT_BD1-V56A_ were mixed with either DMSO or 25 μM JQ1 in a buffer containing 50 mM Tris pH 7.5, 150 mM NaCl with a final DMSO concentration of 2% (v/v) and 2.5X of Sypro Orange. The samples were heated from 25 °C to 95 °C with increments of 1 °C/minute, and fluorescence was measured at each step. Data analysis was performed as previously described (Niesen et al., 2007).

#### Analytical size exclusion chromatography (SEC)

For the analytical runs involving ClpC/C1_NTD_ and mSA or BRDT_BD1_, the proteins were premixed at equimolar concentrations (25 μM) and BacPROTAC-1, BacPROTAC-2, BacPROTAC-3 (25 μM) dissolved in 100% (v/v) DMSO giving rise to a final concentration of 0.25% (v/v). For control experiments, only DMSO was added to that concentration. Samples were loaded into a 30 μL loop and applied to a Superdex 75 3.2/300 increase column (GE healthcare) equilibrated in 50 mM Tris pH 7.5, 100 mM NaCl. Runs were performed at room temperature at a flow rate of 0.06 mL/min. Each run was performed in triplicates. 100 μL fractions were collected and analyzed by SDS-PAGE and Coomassie staining.

For electron microscopy analysis, *B. subtilis* ClpC_DWB_ (25 μM monomer) and the substrate mSA-Kre (25 μM) were premixed in a buffer containing 50 mM Tris pH 7.5, 50 mM KCl, 5 mM MgCl_2_, 5 mM ATP and 1.25% (v/v) DMSO or 156 μM BacPROTAC-1 (thereby adding 1.25% (v/v) DMSO as vehicle). A sample containing *B. subtilis* ClpC_DWB_ (25 μM monomer) and 156 μM BacPROTAC-1 (1.25% (v/v) DMSO as vehicle) without mSA-Kre substrate was also prepared. For co-elution experiments with the arginine-phosphorylated substrate, pArg-β-casein was produced as previously described (Trentini et al., 2016). *B. subtilis* ClpC_DWB_ (25 μM monomer) and the substrate pArg-β-casein (25 μM) were premixed in a buffer containing 50 mM Tris pH 7.5, 50 mM KCl, 5 mM MgCl_2_, 2.5 mM ATP.

For each sample prepared, 30 μL were loaded on a Superose 6 3.2/300 increase column (GE Healthcare) equilibrated in 50 mM Tris pH 7.5, 50 mM KCl, 5 mM MgCl_2_, and 2.5 mM ATP. The experiment was performed at room temperature at a flow rate of 0.06 mL/min. 100 μL fractions were collected and used to prepare grids for EM analysis.

#### *In vitro* degradation assays

*In vitro* degradation assays containing 0.5 μM *B. subtilis* ClpC (hexameric), 0.5 μM *B. subtilis* ClpP (heptameric), 2 μM substrate protein, 15 mM phosphoenolpyruvate (PEP), 10 U/mL pyruvate kinase (Sigma Aldrich) were performed in a buffer containing 50 mM Tris pH 7.5 (at 37 °C), 50 mM KCl, 20 mM MgCl_2_, 10% (v/v) glycerol. Compounds were dissolved in 100% (v/v) DMSO and further diluted giving rise to a final concentration of 1% (v/v) of DMSO in the assay. In control experiments DMSO was added to that concentration. Reactions were started by addition of 5 mM ATP and quenched by adding SDS sample buffer after two hours incubation at 37 °C. The samples were analyzed by SDS-PAGE and Coomassie staining. Degradation assays using 0.5 μM *M. smegmatis* ClpC1 (hexameric), 0.25 μM *M. smegmatis* mature ClpP1P2 (both heptameric), 2 μM substrate protein, 15 mM PEP, 10 U/mL pyruvate kinase (Sigma Aldrich) were carried out in 50 mM HEPES-NaOH pH 7.2, 100 mM KCl, 10 mM MgCl_2_, 10% (v/v) glycerol using the same procedure as described above. All experiments were performed in triplicates which gave similar results.

#### Negative staining EM sample preparation, data collection and processing

In the absence of BacPROTAC-1, ClpC_DWB_ and the substrate mSA-Kre eluted separately in analytical SEC. The fraction containing ClpC_DWB_ was applied onto glow-discharged carbon-coated Cu/Pd Hexagonal 400 mesh grids (Agar Scientific) subsequently stained with a solution of 2% (w/v) uranyl acetate. The grids were screened and then imaged using a FEI Tecnai G2 20 microscope equipped with a 4k Eagle camera (FEI) using a pixel size of 1.8 Å/px. 1,004 micrographs were collected (a representative micrograph is shown in Figure S2A) and 856 particles were manually picked and 2D classified, generating templates for subsequent automatic particle picking on the entire set of micrographs. 369,415 picked particles were extracted using box size of 300 px and subjected to 2D classification. The resulting highest populated 2D class averages are shown in Figure 2B.

In the presence of BacPROTAC-1 alone (without mSA-Kre), ClpC_DWB_ partially shifts to its activated state, eluting as a mixture of active and inactive oligomeric states. The formation of the tetramer of hexamers was confirmed by negative staining EM. The fraction highlighted in Figure S2D was applied onto glow-discharged carbon-coated Cu/Pd Hexagonal 400 mesh grids (Agar Scientific) subsequently stained with a solution of 2% (w/v) uranyl acetate. The grids were imaged using a FEI Morgagni microscope equipped with a Morada camera (Olympus-SIS) using a pixel size of 4.7 Å/px. 2 micrographs were collected (a representative area is shown in Figure S2E) and 1441 particles were manually picked, extracted using box size of 120 px and 2D classified; the resulting highest populated 2D class averages are shown in Figure S2E.

The fraction highlighted in Figure S2D (containing the tetramer of ClpC hexamers) was used to reconstitute the ClpCP complex by addition of 1.5 μM of purified ClpP (relative to monomer). The sample was applied onto glow-discharged carbon-coated Cu/Pd Hexagonal 400 mesh grids (Agar Scientific) and stained with a solution of 2% (w/v) uranyl acetate. The grids were screened and then imaged using a FEI Tecnai G2 20 microscope equipped with a 4k Eagle camera (FEI) using a pixel size of 1.8 Å/px. 84 micrographs were collected (a representative micrograph is shown in Figure S2C) and 1358 particles were manually picked, extracted using box size of 500 px and 2D classified. The resulting highest populated 2D class averages are shown in Figure 2G.

In the presence of equimolar amounts (relative to ClpC monomer) of arginine-phosphorylated β-casein, ClpC_DWB_ fully shifts to its activated state. The formation of the tetramer of hexamers was confirmed by negative staining EM. The fraction highlighted in Figure S2F was applied onto glow-discharged carbon-coated Cu/Pd Hexagonal 400 mesh grids (Agar Scientific) subsequently stained with a solution of 2% (w/v) uranyl acetate. The grids were imaged using a FEI Morgagni microscope equipped with a Morada camera (Olympus-SIS) using a pixel size of 4.7 Å/px. 4 micrographs were collected (a representative area is shown in Figure S2G) and 2877 particles were manually picked, extracted using box size of 120 px and 2D classified; the resulting highest populated 2D class averages are shown in Figure S2G.

#### Cryo-EM sample preparation and data collection

The ClpC_DWB_:BacPROTAC-1:mSA-Kre complex was isolated using analytical SEC. Cryo-EM grids were prepared using glow discharged R2/2 Cu 200 mesh grids (Quantifoil) pre-floated onto custom-made 2.9 nm continuous carbon film. 4 μL sample were applied onto a grid held in the sample grid chamber of a Leica EM GP instrument (Leica Microsystem Inc.) cooled at 4 °C, with a relative humidity of 80-90%. Grids were blotted 2 s with a Whatman type 1 filter paper using the blotting sensor and flash frozen in liquid ethane. The quality of the grids was screened using a Thermo Scientific Glacios Cryo Transmission Electron Microscope equipped with a Falcon3 Direct Electron Detector. For the best grid, a dataset of 4,455 micrographs was collected (a representative micrograph is shown in Figure S2B) on a Titan Krios equipped with a Falcon 3EC detector with a nominal magnification of 75,000 resulting in pixel size of 1.058 Å/px. A total dose of 54 e^-^/Å^2^ was fractionated into 40 frames. 452 poor quality images (heavy contamination, poor ice quality or bad information transfer) were discarded after visual inspection, leaving 4,003 images for further analysis.

#### Cryo-EM analysis of the ternary complex between ClpC, BacPROTAC-1 and mSA: the tetramer of hexamers

Multi-frame micrographs were analyzed in cryoSPARC v2 (Punjani et al., 2017). The full micrographs were motion corrected and their contrast transfer function (CTF) parameters were estimated. 482 particles were manually picked and 2D classified, generating a template for subsequent automatic particle picking on the entire set of micrographs. 1,173,558 picked particles were extracted using box size of 500 px and subjected to two rounds of 2D classification, selecting only the classes containing the full tetramer of hexamers for further analysis. 87,575 “clean” particles were used for *ab initio* model generation and subsequent homogeneous refinement applying T symmetry. The approximate resolution of the obtained map was 10 Å as judged by Gold Standard Fourier shell correlation (FSC) (Figure S3A).

#### Cryo-EM analysis of the ternary complex between ClpC, BacPROTAC-1 and mSA: the hexameric building block

For the analysis of the single hexamers, the same multi-frame micrographs were motion corrected and dose-weighted using MotionCor2 1.0.5 (Zheng et al., 2017). Aligned micrographs were used for CTF estimation in Gctf 1.06 (Zhang, 2016). A subset of 10 micrographs was used for manual particle picking in crYOLO v1.3.5 (Wagner et al., 2019), the picking centers were the single hexamers rather than the tetramer of hexamers. Manually picked particles were used to train a model for automatic particle picking on the full micrographs set (160 px box size). A total of 1,034,627 auto-picked particles were imported in RELION 3.0 (Zivanov et al., 2018). Particles were extracted with 2 × 2 binning (box size 300 px, rescaled to 150 px) and subjected to two rounds of 2D classification. The particles belonging to the best 2D classes (Figure 3A) were re-extracted un-binned (box size 300 px) and 3D classified using an initial 3D model generated with cryoSPARC v2 (Punjani et al., 2017). After 3D classification, 212,314 particles were selected for 3D refinement. The resolution was estimated with Gold Standard FSC, and B-factor sharpening was applied, both using the RELION postprocessing. The final map used for model building had an overall resolution of 3.7 Å. Local resolution was estimated in RELION and is shown in Figure S3C.

#### Model building

An initial model was built in Coot (Emsley et al., 2010) by rigid body fitting of secondary structure elements of one of the previously reported ClpC structures into the EM-map (PDB: 3J3U) (Liu et al., 2013). This model, however, required partial rebuilding and several steps of manual real-space refinement in Coot. Substrate-bound ClpB cryo-EM structures (Deville et al., 2019) were used as reference, as mSA-Kre binding caused substantial conformational rearrangements in the unfoldase hexamer when compared to the apo form of ClpC reported previously (Liu et al., 2013; Wang et al., 2011). The substrate polypeptide chain was modelled as a poly-alanine. Phenix (Afonine et al., 2018) was used for further real-space refinement. The quality of the model and model to map fitting were assessed using Phenix (Afonine et al., 2018), MolProbity (Williams et al., 2018) and EMRinger (Barad et al., 2015). Structural illustrations were prepared using either UCSF Chimera (Goddard et al., 2007), UCSF ChimeraX (Goddard et al., 2018), or Pymol (Schrödinger, 2015).

#### ClpC1_NTD_:sCym-1 co-crystallization and structure determination

ClpC1_NTD_ in a buffer containing 10 mM Tris-Cl pH 7.5, 100 mM NaCl, 1 mM sCym-1, and 1% (v/v) DMSO was crystallized in a hanging drop, vapor diffusion setup at 15 mg/mL concentration using a reservoir solution containing 100 mM MES/imidazole pH 6.5, 6% (w/v) PEG 20K, 12% (v/v) PEG MME 550, 120 mM 1,6-hexanediol, 120 mM 1-butanol, 120 mM (*RS*)-1,2-propanediol, 120 mM 2-propanol and 120 mM 1,4-butanediol. Crystals were grown at room temperature for one week, subsequently harvested and flash-cooled in liquid nitrogen. Diffraction data were collected at an in-house X-ray generator and processed, scaled using the XDS (Kabsch, 2010) package to a resolution of 1.7 Å. Initial phases were obtained by Molecular Replacement using PHASER and the structure of ClpC1_NTD_ (PDB: 3WDB) (Vasudevan et al., 2013) as starting model. The model was improved in iterative cycles of manual building using Coot (Emsley and Cowtan, 2004) and refinement with Phenix (Liebschner et al., 2019) omitting 5% of randomly selected reflections for calculation of R_free_. Model quality was monitored using MolProbity (Williams et al., 2018) and the final model exhibited good stereochemistry with 97.5% of residues in favored regions of the Ramachandran plot and without any outliers (Table S1). Structural illustrations were made using Pymol (Schrödinger, 2015).

#### Genetic manipulations in *M. smegmatis*

Plasmid DNA for BRDT_BD1_ expression in *M. smegmatis* mc^2^-155 containing the coding region of BRDT_BD1_ cloned into pMyC vector DNA (pMyC BRDT_BD1_) was ordered from Genescript. pMyC was a gift from Annabel Parret & Matthias Wilmanns (Addgene plasmid # 42192). Site directed mutagenesis was performed to obtain an expression plasmid pMyC-BRDT_BD1-V56A_. Gene blocks for the codon optimized coding region of the DdlA-BRDT_BD1_-fusion protein were ordered from Integrated DNA technologies and cloned into the pMyC vector DNA via Gibson assembly. DdlA alone was cloned from DdlA-BRDT_BD1_ by excising BRDT resulting in a pMyC vector carrying DdlA alone. For expression of pMyC-BRDT-ThrC, the coding region of ThrC was amplified from genomic DNA, isolated from an exponential growing culture of ATCC 7000084 as described in (Belisle et al., 2009), and fused with pMyC-BRDT via Gibson Assembly. *M. smegmatis thrC::BRDT*_*BD1*_*-thrC* was obtained as a single cross-over from the parental strain *M. smegmatis* mc^2^-155 (ATCC 7000084) by homologous recombination as previously described (Kendall and Frita, 2009). Briefly, a construct containing the 5’′ homology region of *thrC* including its start codon as well as an additional serine codon, the BRDT_BD1_ plus linker coding region, and the 3′ homology region comprising the entire *thrC* coding region and a 420 bp fragment from the downstream gene was integrated into the p2NIL (a gift from Tanya Parish; addgene plasmid # 20188) via Gibson assembly. DNA was PCR amplified from genomic or pMyC(BRDT_BD1_) DNA, respectively using the primers listed in Table S3. After insertion of the appropriate selection cassettes excised from pGOAL-19 (Parish and Stoker, 2000) (a gift from Tanya Parish; addgene plasmid # 20190) in the modified p2NIL, vector DNA was transformed and integrated into *M. smegmatis* mc^2^ 155. The original protocol from Kendall *et al.* (Kendall and Frita, 2009) was optimized by increasing antibiotic strength and recovery after each selection step. Clones which have recombined via the 5′ homology regions in a single cross-over were identified by PCR using the primers listed in Table S3. Cloning and vector DNA amplification was performed in *E. coli* XL10Gold following standard procedures. Transformation of expression vectors into *M. smegmatis* was performed according to (Goude and Parish, 2008). Correctness of all plasmids was verified by DNA sequencing prior to transformation into *M. smegmatis*.

#### Cultivation and Susceptibility Tests Using *M. smegmatis*

*M. smegmatis* was grown in 7H9 medium supplemented with the required antibiotics and grown under constant agitation at 37 °C. For *in vivo* degradation assays, protein expression was induced upon addition of 0.1% (w/v) acetamide when culturing *M. smegmatis* transformed with the respective pMyC-POI vectors. Cells were harvested by centrifugation and resuspended in small amounts of prewarmed 7H9. Cultivation time after expression, induction as well as concentration factors after cell harvest required individual optimization for each POI to obtain cellular protein levels suitable to be monitored within the dynamic range of detection of the western capillary electrophoresis experiment.

Phenotype monitoring of BID for BRDT_BD1_-degron fusion constructs with DdlA or ThrC was performed by determination of the Minimum inhibitory concentration (MIC) of individual compounds using an agar-based assay similar as described previously (Blanco-Ruano et al., 2015; Sirgel et al., 2009). Briefly, DMSO dilution series of individual compounds were made in standard 96-well PCR plates. 1.5 μL aliquots of compound dilutions were transferred into final flat 96-well plates (Thermo Nunc Microwell). 150 μL of molten 7H10 agar at 45-50 °C was added and cooled to ambient temperature. Cells cultured in liquid medium were diluted in prewarmed medium to a final concentration of 1 x 10^5^ cfu/mL and 5 μL were spotted into each well and incubated at 37 °C for 2.5-5 days before visual inspection. Initially, wild type strains were used to determine potential toxic effects of BacPROTACs or the individual head groups.

BID of DdlA_BRDT_BD1_ was studied in a classical checkerboard assay adapted from (Feng and Barletta, 2003). Sensitivity to increasing concentrations of D-cycloserine (DCS) was determined in the background of DdlA_BRDT_BD1_, DdlA expression or an empty vector control. This was performed in presence of either functional BacPROTAC or control variants at sub-MIC concentration. To ensure sufficient expression of the individual proteins, liquid cultures were induced 90 minutes before spotting and the agar was supplemented with 0.1% (w/v) acetamide and 100 μg/mL hygromycin.

When monitoring the L-threonine auxotrophy after BID, *M. smegmatis thrC::BRDT*_*BD1*_*-thrC* was grown to exponential growth phase in liquid culture, diluted and spotted on MIC plates with decreasing BacPROTAC concentration. In control experiments, auxotrophy was cured by addition of 1 mM L-threonine and potential reversion to the wild-type strain was excluded by control PCR as described above.

#### Wes analysis of BID *in vivo*

Aliquots of a dense cell suspension cultured as described above were mixed with various concentrations of the different BacPROTAC variants or their individual head groups in 96-well glass-coated microplates (Thermo Scientific). All dilutions were performed to yield a final concentration of 1% (v/v) DMSO in the medium. Aliquots of 250 μL of each cell suspension were withdrawn at the indicated timepoints, cells were harvested by centrifugation and resuspended in 100 μL of a buffer containing 50 mM HEPES-NaOH pH 7.2, 150 mM KCl, 10% (v/v) glycerol. Cells were lysed for 10 minutes using the Bioruptor (Diagenode, 10 cycles, 30 seconds on - 30 seconds off) and the cell debris was cleared by centrifugation.

Cytosolic amounts of BRDT_BD1_ and RpoB in the lysates were analysed using Wes (ProteinSimple). Bacterial lysates were diluted 2-fold and analyzed using the Protein Simple 12-230 kDa Wes Separation Module following the manufacturer’s instructions. Anti-BRDT (Bio-Vision, rabbit) and anti-RpoB (BioLegend, mouse) antibodies were combined in a single primary antibody mixture for simultaneous BRDT_BD1_ and RpoB detection. The primary antibody mixture contained anti-BRDT diluted 1:250 and anti-RpoB diluted 1:25,000 or 1:10,000, respectively. BRDT_BD1_ and RpoB were detected using the Anti-Mouse (Protein Simple) and Anti-Rabbit (ProteinSimple) detection modules for Wes following the manufacturer’s instruction. Anti-Mouse antibodies were combined 1:10 or 1:30, respectively, and anti-Rabbit was used undiluted and the obtained secondary antibody mixtures were used for detection. Results were analyzed using the Compass for SW software (ProteinSimple). Compass displays the chemiluminescent signal detected along the length of the capillaries as electropherograms, where the intensity of the chemiluminescent signal is plotted against the apparent molecular weight (Exemplary traces Data S1). A statistical comparison is given in Data S1 based on the integrated peak areas summarized in Table S4). Detected peaks were quantified and include a peak at the expected BRDT_BD1_ molecular weight (BRDT_BD1_ MW = 16.6 kDa) and an additional peak at the expected RpoB molecular weight (MW = 128.5 kDa). Peak areas were normalized to DMSO-treated cells and plotted as mean ± SD.

#### Sample preparation for quantitative mass spectrometry analysis

For quantitative MS analysis, *M. smegmatis* cells expressing BRDT_BD1_ were treated for two hours with 1% (v/v) DMSO, 100 μM, 10 μM, or 1 μM BacPROTAC-3, following the procedures described above. Cleared lysates were processed according to the single-pot SP3 protocol (Hughes et al., 2019) for low input proteomics sample preparation. Each lysate of 100 μl was reduced with 10 mM dithiothreitol (DTT, Sigma Aldrich) for 45 minutes at 37 °C and subsequently alkylated with 20 mM iodoacetamide (IAA, Sigma Aldrich) at room temperature for 1 hour. In parallel, a 1:1 mixture of 50 mg/mL Sera-Mag SpeedBeads, variant 1 (GE Healthcare) and 50 mg/mL Sera-Mag SpeedBeads, variant 2 (GE Healthcare), exhibiting different surface hydrophilicity, was prepared in water. To each lysate, 15 μL of the prepared SP3 bead stock was added and binding was induced by the addition of 100 μL ethanol. To ensure proper binding, samples were incubated on a shaker for 5 minutes at 24 °C and 1000 rpm. After protein binding, beads were washed three times with 200 μL rinsing solution (80% (v/v) ethanol in water) while being kept on a magnetic rack. Protein elution from the beads was enforced by addition of 100 mM triethylammonium bicarbonate (pH = 8.5, Sigma Aldrich). To disaggregate the beads, the tubes were shortly sonicated in a water bath. For protein digestion 1:25 wt/wt ratio of trypsin to protein was added and the samples were incubated overnight at 37 °C in a thermo-shaker at 1,000 rpm.

Peptides were labelled for quantification in a multiplexed setup with TMT isobaric mass tags (TMTpro™ 16plex). Sample amount and quality was determined by HPLC-UV using a Dionex UltiMate 3000 HPLC RSLC nanosystem with a PepSwift Monolithic RSLC column (0.2 x 5 mm, Thermo Fisher Scientific) at 60 °C. Peptides were separated using a 20 minutes 2-90% elution gradient of buffer A (80% (v/v) ACN, 0.1% (v/v) TFA in aqueous solution). For the labelling procedure, one TMTpro set was split into three aliquots to label three replicates. 14 channels (TMTpro 126-133N) were distributed over two timepoints for each of the seven treatments. The two remaining channels (133C and 134N) were used as reference channels with pools of all samples. Each sample was tagged with an excess of the respective TMT labelling reagent (1:20, peptide:TMT label) and incubated at room temperature for one hour. The reaction was quenched by addition of 5 μL 5% (v/v) hydroxylamine (Sigma Aldrich), followed by a 15 min incubation step. For each replicate, all 16 channels were pooled, and the volume was reduced to 100 μL in a vacuum concentrator. Removal of excess TMT labelling reagent was achieved by running the samples through tips filled with silica gel equilibrated in water.

#### High pH chromatography and LC-MS/MS analysis

Peptides were separated using a 40 min 2-50% gradient of buffer A in a high pH chromatography (TEA, pH = 8.5) setup using a Dionex UltiMate 3000 HPLC RSLC nanosystem with a XBridge Peptide BEH C18 Column (1 x 150 mm, 130 Å, 3.5 μm, Waters). 40 fractions were collected and pooled by combining every 11^th^ fraction to generate a final number of 10 fractions for each replicate. The volume of each sample was adjusted to 100 μL and the sample amount was estimated by running monolithic control runs.

LC-MS/MS analysis was performed on a Dionex UltiMate 3000 HPLC RSLC nanosystem using an Acclaim PepMap C-18 precolumn (0.3 x 5 mm, Thermo Fisher Scientific) and an Acclaim PepMap C-18 column (50 cm x 75 μm, Thermo Fisher Scientific) coupled to a Q Exactive HF-X hybrid quadrupole-Orbitrap mass spectrometer (Thermo Fisher Scientific). Peptides were separated using a 120 min linear gradient of 2-35% buffer A at a flowrate of 230 nL/min. MS1 spectra were generated in a 380-1,650 *m/z* mass range at a 120,000 orbitrap resolution, AGC target of 3e6, with a maximum injection time of 50 ms. The top 10 precursors were selected for MS2 analysis using a 0.7 *m/z* quadrupole precursor isolation window, allowing charge states 2-4 and a dynamic precursor exclusion of 30 s. The Orbitrap was operated at 45,000 resolution with an AGC of 1e5 and a NCE of 35 at a maximum injection time of 250 ms.

#### MS data analysis

MS raw data were analyzed using Proteome Discoverer 2.3 (PD 2.3.0.523, Thermo) and the search was performed using the search engine MS Amanda (Dorfer et al., 2014) against a database of the *M. smegmatis* 2019 Uniprot Reference Proteome with contaminants and the BRDT_BD1_ protein added. The database search allowed tryptic peptides with two missed cleavages at a precursor mass tolerance of 5 ppm and 0.02 Da MS2 tolerance. Static alkylation of cysteine and variable oxidation of methionine and TMTpro adducts on lysine and peptide N-termini were considered. Peptides were scored and filtered using Percolator (Käll et al., 2007) to obtain peptides at a 1% false discovery rate. Reporter ions were quantified using the IMP Hyperplex (https://ms.imp.ac.at/?goto=pd-nodes) at a reporter mass tolerance of 10 ppm with a MS2 precursor threshold of 10. The search was performed for each replicate separately over the ten fractions.

Statistical analysis and data normalization were performed in R. The samples were first normalized for different sample loading by their total sum within each replicate set and then the three TMT replicates were normalized using the Internal Reference Scaling (IRS) method (Plubell et al., 2017). Median alignment was done afterwards by TMM normalization. For each protein, the fold change of TMT-intensities and the corresponding p value (two-tailed Student’s T-test,) were calculated. Permutation-based FDR calculation was used to assess the q-values. The mass spectrometry proteomics data have been deposited to the ProteomeXchange Consortium via the PRIDE (Perez-Riverol et al., 2019) partner repository with the dataset identifier PXD021505.

#### Chemical Synthesis of BacPROTACs

Individual synthetic procedures, NMR spectra as well as the HPLC traces are provided in Methods S1.

### Quantification and statistical analysis

Reported ITC K_D_ values represent the average ± SD of the indicated number of independent measurements.

All Wes data are represented as mean ± SD of the indicated replicate experiments. Vulcano plots for TMT-MS proteomics analysis show the fold-change (log_2_) in protein abundance in comparison to DMSO treatment, plotted against *P* value (–log_10_) (two-tailed Student’s T-test; triplicate analysis).

Melting temperatures (T_m_ - °C) derived from triplicate DSF measurements are plotted as mean ± SD.

## Data Availability

Coordinates of the ClpC_NTD_:sCym-1 crystal structure have been deposited at the Protein Data Bank (PDBe) under accession code 7AA4. Cryo-EM maps and atomic coordinates have been deposited in the EMDB and PDB with accession codes EMD-11708 for the ClpC tetramer-of-hexamers; EMD-11707 and PDB 7ABR for the single ClpC hexamer composing the assembly. The raw micrographs were submitted to the EMPIAR database (deposition ID: 847). The mass spectrometry proteomics data have been deposited to the ProteomeXchange Consortium via the PRIDE partner repository with the dataset identifier PXD021505. The paper does not report original code. Any additional information required to reanalyze the data reported in this paper is available from the lead contact upon request.
