# Supplementary material for: BacPROTACs mediate targeted protein degradation in bacteria
Source: Cell. 2022 Jun 23;185(13):2338–2353.e18. doi: 10.1016/j.cell.2022.05.009 (PMC9240326; doi:10.1016/j.cell.2022.05.009)
Supplement: Document S1. Tables S1–S4, Data S1, and Methods S1 [file mmc1.pdf]

Cell, Volume *185*

Supplemental information

BacPROTACs mediate targeted

protein degradation in bacteria

Francesca E. Morreale, Stefan Kleine, Julia Leodolter, Sabryna Junker, David M. Hoi, Stepan Ovchinnikov, Anastasia Okun, Juliane Kley, Robert Kurzbauer, Lukas Junk, Somraj Guha, David Podlesainski, Uli Kazmaier, Guido Boehmelt, Harald Weinstabl, Klaus Rumpel, Volker M. Schmiedel, Markus Hartl, David Haselbach, Anton Meinhart, Markus Kaiser, and Tim Clausen

SUPPLEMENTAL INFORMATION

BacPROTACs mediate targeted protein degradation in bacteria

Francesca Ester Morreale[1], Stefan Kleine[2], Julia Leodolter[1], Sabryna Junker[1], David M. Hoi[1], Stepan Ovchinnikov[1], Anastasia Okun[1], Juliane Kley[1], Robert Kurzbauer[1], Lukas Junk[3,4], Somraj Guha[3], David Podlesainski[2], Uli Kazmaier[3,4], Guido Boehmelt[5], Harald Weinstabl[5], Klaus Rumpel[5], Volker M. Schmiedel[5], Markus Hartl[6], David Haselbach[1], Anton Meinhart[1], Markus Kaiser[2*], Tim Clausen[1,7,8*]

Table S1. Crystallographic analysis of ClpC1$_{NTD}$:sCym-1 complex. Data collection and refinement statistics, Related to Figure 4D

**PDB ID**	**7AA4**
Space group	*P*1
Cell dimensions	
a, *b*, *c* (Å)	31.35, 33.68, 35.81
α, *β*, *γ* (°)	86.178, 94.216, 103.176
Resolution (Å)[a,b]	25 – 1.68 (1.72 – 1.68)
$R_{meas(I)}$	0.062 (0.112)
I/σ (I)	22.6 (12.5)
$CC_{1/2}$	0.998 (0.992)
Completeness (%)	93.8 (86.5)
Redundancy	5.8 (5.4)
Resolution (Å)	25 – 1.68
No. reflections	15,165
R_{work} / R_{free}	17.0 / 20.3
No. atoms	
protein	1254
ligand	66
water	192
B factors	
protein	11.9
ligand	13.5
water	23.0
R.m.s. deviations	
Bond lengths (Å)	0.006
Bond angles (°)	0.789

[a]Values in parentheses are for highest-resolution shell.

[b]Due to experimental constraints the resolution needed to be truncated to this resolution.

Table S2. Amino acid sequences of fusion proteins, Related to STAR Methods

**Construct name**	**Amino acid sequence**
mSA	MHHHHHHSSGVDLGTENLYFQSSQDLASAEAGITGTWYNQSGSTFTVTAGAD<br>GNLTGQYENRAQGTGCQNSPYTLTGRYNGTKLEWRVEWNNSTENCHSRTEW<br>RGQYQGGAEARINTQWNLTYEGGSGPATEQGQDTFTKVKPSAASGSGSGSGS<br>GS
mSA-Kre	MHHHHHHSSGVDLGTENLYFQSSQDLASAEAGITGTWYNQSGSTFTVTAGAD<br>GNLTGQYENRAQGTGCQNSPYTLTGRYNGTKLEWRVEWNNSTENCHSRTEW<br>RGQYQGGAEARINTQWNLTYEGGSGPATEQGQDTFTKVKPSAASGSGSGSGS<br>MDDHAYTKDLQPTVENLSKAVYTVNRHAKTAPNPKYLYLLKKRALQKLVKEGKG<br>KKIGLHFSKNPRFSQQQSDVLISIGDYYFHMPPTKEDFEHLPHLGTLNQSYRNP<br>KAQMSLTKAKHLLQEYVGMKEKPLVPNRQQPAYHKPVFKKLGESYF
mSA-Kre<br>(Cryo-EM structure determination)	MSQDLASAEAGITGTWYNQSGSTFTVTAGADGNLTGQYENRAQGTGCQNSPY<br>TLTGRYNGTKLEWRVEWNNSTENCHSRTEWRGQYQGGAEARINTQWNLTYE<br>GGSGPATEQGQDTFTKVKPSAASGSGSGSGSGSGSGSGSGSDDHAYTKDLQ<br>PTVENLSKAVYTVNRHAKTAPNPKYLYLLKKRALQKLVKEGKGKKIGLHFSKNP<br>RFSQQQSDVLISIGDYYFHMPPTKEDFEHLPHLGTLNQSYRNPKAQMSLTKAKH<br>LLQEYVGMKEKPLVPNRQQPAYHKPVFK KLGESYFHHHHHH
mSA-NrdI	MHHHHHHSSGVDLGTENLYFQSSQDLASAEAGITGTWYNQSGSTFTVTAGAD<br>GNLTGQYENRAQGTGCQNSPYTLTGRYNGTKLEWRVEWNNSTENCHSRTEW<br>RGQYQGGAEARINTQWNLTYEGGSGPATEQGQDTFTKVKPSAASGSGSGSGS<br>GSVVQIIFDSKTGNVQRFVNKTGFQQIRKVDEMDHVDTPFVLVTYTTNFGQVPA<br>STQSFLEKYAHLLLGVAASGNKVWGDNFAKSADTISRQYQVPILHKFELSGTSK<br>DVELFTQEVERVVTKSSAKMDPVK
mSA-TagD	MHHHHHHSSGVDLGTENLYFQSSQDLASAEAGITGTWYNQSGSTFTVTAGAD<br>GNLTGQYENRAQGTGCQNSPYTLTGRYNGTKLEWRVEWNNSTENCHSRTEW<br>RGQYQGGAEARINTQWNLTYEGGSGPATEQGQDTFTKVKPSAASGSGSGSGS<br>GSMKKVITYGTFDLLHWGHIKLLERAKQLGDYLVVAISTDEFNLQKQKKAYHSYE<br>HRKLILETIRYVDEVIPEKNWEQKKQDIIDHNIDVFVMGDDWEGKFDFLKDQCEV<br>VYLPRTEGISTTKIKEEIAGL
mSA-NusA	MHHHHHHSSGVDLGTENLYFQSSQDLASAEAGITGTWYNQSGSTFTVTAGAD<br>GNLTGQYENRAQGTGCQNSPYTLTGRYNGTKLEWRVEWNNSTENCHSRTEW<br>RGQYQGGAEARINTQWNLTYEGGSGPATEQGQDTFTKVKPSAASGSGSGSGS<br>GSMSSELLDALTILEKEKGISKEIIIEAIEAALISAYKRNFNQAQNVRVDLNRETGSI<br>RVFARKDVVDEVYDQRLEISIEEAQGIHPEYMVGDVVEIEVTPKDFGRIAAQTAK<br>QVVTQRVREAERGVIYSEFIDREEDIMTGIVQRLDNKFIYVSLGKIEALLPVNEQM

	PNESYKPHDRIKVYITKVEKTTKGPQIYVSRTHPGLLKRLFEIEVPEIYDGTVELKS VAREAGDRSKISVRTDDPDVDPVGSCVGPKGQRVQAIVNELKGEKIDIVNWSSD PVEFVANALSPSKVLDVIVNEEEKATTVIVPDYQLSLAIGKRGQNARLAAKLTGW KIDIKSETDARELGIYPRELEEDDEPLFTEPETAESDE
$BRDT_{BD1}$	MHHHHHHSSGVDLGTENLYFQSMNTKKNGRLTNQLQYLQKVVLKDLWKHSFS WPFQRPVDAVKLQLPDYYTIIKNPMDLNTIKKRLENKYYAKASECIEDFNTMFSN CYLYNKPGDDIVLMAQALEKLFMQKLSQMPQEE
$BRDT_{BD1\text{-}V56A}$	MHHHHHHSSGVDLGTENLYFQSMNTKKNGRLTNQLQYLQKVVLKDLWKHSFS WPFQRPADAVKLQLPDYYTIIKNPMDLNTIKKRLENKYYAKASECIEDFNTMFSN CYLYNKPGDDIVLMAQALEKLFMQKLSQMPQEE
DdlA-$BRDT_{BD1}$	MTAPNHPPGRTRVAVVYGGRSSEHAISCVSAGSILRNLDPERFEVVAIGITPDGS WVLTDGRPETLAITDGKLPAVTEASGTELALPAAPNRSGQLLALGNGPGEILAAV DVVFPVLHGPYGEDGTIQGLLELAGVPYVGSGVLASAAGMDKEYTKKLLAAEGL PIGDQVVLRPGVETLDLEQRERLGLPVFVKPARGGSSIGVSRVTAWDELPAAVA LARRHDPKVIVEAAVIGRELECGVLEFPDGRLEASTVGEIRVAGVRGREDGFYD FATKYLEDAAELDVPAKVDDDVADEIRQLAVRAFTAIGCQGLARVDFFLTDDGP VINEINTMPGFTTISMYPRMWAAGGIDYPTLLAAMVDTAIARGTGLRTDSGSGS GSGSGSMHHHHHHSSGVDLGTENLYFQSMNTKKNGRLTNQLQYLQKVVLKDL WKHSFSWPFQRPVDAVKLQLPDYYTIIKNPMDLNTIKKRLENKYYAKASECIEDF NTMFSNCYLYNKPGDDIVLMAQALEKLFMQKLSQMPQEE
DdlA	MAMTAPNHPPGRTRVAVVYGGRSSEHAISCVSAGSILRNLDPERFEVVAIGITP DGSWVLTDGRPETLAITDGKLPAVTEASGTELALPAAPNRSGQLLALGNGPGEI LAAVDVVFPVLHGPYGEDGTIQGLLELAGVPYVGSGVLASAAGMDKEYTKKLLA AEGLPIGDQVVLRPGVETLDLEQRERLGLPVFVKPARGGSSIGVSRVTAWDELP AAVALARRHDPKVIVEAAVIGRELECGVLEFPDGRLEASTVGEIRVAGVRGRED GFYDFATKYLEDAAELDVPAKVDDDVADEIRQLAVRAFTAIGCQGLARVDFFLTD DGPVINEINTMPGFTTISMYPRMWAAGGIDYPTLLAAMVDTAIARGTGLR
$BRDT_{BD1}$-ThrC	MHHHHHHSSGVDLGTENLYFQSMNTKKNGRLTNQLQYLQKVVLKDLWKHSFS WPFQRPVDAVKLQLPDYYTIIKNPMDLNTIKKRLENKYYAKASECIEDFNTMFSN CYLYNKPGDDIVLMAQALEKLFMQKLSQMPQEEGSGSGSGSGSMSAAKAAVH QPWPGLIEAYRDRLPIGDDWTTVTLLEGGTPLIHAKRISELTGCTVHLKVEGLNP TGSFKDRGMTVAVTESLARGQQAVLCASTGNTSASAAAYAARAGITCAVLIPQG KIAMGKLAQAVMHGAKIIQVDGNFDDCLELARKLTADFPTIALVNSVNPYRIEGQ KTAAFEIVDALGTAPDVHALPVGNAGNITAYWKGYSEYHRDGVSDRLPRMLGT QAAGAAPLVTGAPVKDPETIATAIRIGSPASWNSAVEAQQQSDGRFLAATDEEIL AAYHLVARTEGVFVEPASAASIAGLLKSVEDGWVKRGSTVVCTVTGNGLKDPD TALKGMPQVTPVPVDPSAVVAELGLS

Table S3. Primer sequences, Related to STAR Methods

pMyC-BRDT-ThrC

Primer description	sequence
upstream region of *thrC* forward	CCGACGACATCGCGCCCGGCGACCTG
downstream region of *thrC* reverse	Gacagctggatcagccgtgcgtcgtc
thrC insert PCR forward	GGCTCCGGATCTGGTAGCGGTTCGGGCTCCATGAGTGCAGCAAAGGCTGCGGTGCAC
thrC insert PCR reverse	TTACTAGCTCAGACCCAGCTCGGCGAC
pMyC-BRDT vector PCR forward	AAGCTTATCGATGTCGACGTAGTTAAC
pMyC-BRDT vector PCR reverse	CGCTACCAGATCCGGAGCCCTCTTCCTGCGGCATC

Genomic insertion of BRDT-ThrC

Primer description	sequence
thrC upstream homologous region forward	cactatagaatacataGGATCCccgacgacatcgcgc
thrC upstream homologous region reverse	GTGGTGGTGGTGGTGactCATgagttcgttccttccagtc
ser+*brdt* forward	GgaaggaacgaactcATGagtCACCACCACCACCACC
thrC reverse	gaatgatccccgCTAGCTCAGACCCAGCTCG
thrC downstream homologous region forward	GGTCTGAGCTAGcggggatcattcggtgac
thrC downstream homologous region reverse	gataaactaccgcattaAAGCTTgacagctggatcagcc
p2NIL vector PCR forward	cgatgtcgtcggGGATCCtatgtattctatagtgtcacc
p2NIL vector PCR reverse	gatccagctgtcAAGCTTtaatgcggtagtttatcacagttaaattgc
Control PCR to identify 5’ recombination events - forward	gagggcatcaccacggtc
Control PCR to identify 5’ recombination events - reverse	CTTGAGATGCACTGTGCAGCC

Table S4. Wes integrated peak areas, Related to Figure 5

Table S4 lists the integrated peak areas of the Wes chemiluminescence signal of $BRDT_{BD1}$ and RpoB antibody detection (BRDT_raw, RpoB_raw) for the respective figure panels. Data of individual experiments are labeled alphabetically. A280 of bacterial lysates was used for concentration adjustment. See also Data S1.

Figure	Experiment	target	compound	BRDT_raw	RpoB_raw	A280
5D	A	BRDT	DMSO	521036.7	1720850.6	4.7
5D	A	BRDT	DMSO	561462.4	1846481	4
5D	A	BRDT	DMSO	502044	1842896.9	3.9
5D	A	BRDT	100 µM BacPROTAC-3	227202.1	1829080.7	4.1
5D	A	BRDT	100 µM BacPROTAC-3	218871	1761728	4
5D	A	BRDT	100 µM BacPROTAC-3	149352.2	1754197.3	3.7
5D	A	BRDT	100 µM BacPROTAC-3/1mM JQ1	697210	1716366.3	4.6
5D	A	BRDT	100 µM BacPROTAC-3/1mM JQ1	458561.9	1738489.4	4.7
5D	A	BRDT	100 µM BacPROTAC-3/1mM JQ1	511596.5	1781983.1	4.5
5D	B	BRDT	DMSO	702354.1	643560.6	5.3
5D	B	BRDT	DMSO	671671.6	645217.6	4.6
5D	B	BRDT	DMSO	620156.5	640112.6	5
5D	B	BRDT	100 µM BacPROTAC-3a	691738.2	673925.6	5.5
5D	B	BRDT	100 µM BacPROTAC-3a	702593.3	660169	6
5D	B	BRDT	100 µM BacPROTAC-3a	747222.6	702407.7	5.5
5D	C	BRDT_V56A	DMSO	1809482.7	1823068.7	4.3
5D	C	BRDT_V56A	DMSO	1400629.6	1825679.7	4.1
5D	C	BRDT_V56A	DMSO	1512563.1	1829303	4.1
5D	C	BRDT_V56A	100 µM BacPROTAC-3	1414819.2	1723883.5	4
5D	C	BRDT_V56A	100 µM BacPROTAC-3	1766954.7	1726168.9	4.3
5D	C	BRDT_V56A	100 µM BacPROTAC-3	1892485.3	1766036.8	4.7
5E	A	BRDT	DMSO	702354.1	643560.6	5.3
5E	A	BRDT	DMSO	671671.6	645217.6	4.6
5E	A	BRDT	DMSO	620156.5	640112.6	5
5E	A	BRDT	20 µM BacPROTAC-4	521254.2	707664.9	5.2

5E	A	BRDT	20 µM BacPROTAC-4	522457.6	591618.3	5.5
5E	A	BRDT	20 µM BacPROTAC-4	581572.7	825022.5	5.4
5E	A	BRDT	20 µM BacPROTAC-4a	710488.3	758713.6	5.3
5E	A	BRDT	20 µM BacPROTAC-4a	799836	780639.2	5.6
5E	A	BRDT	20 µM BacPROTAC-4a	753450.4	1056154.9	5.8
S6C	A	DdlA-BRDT	DMSO	5511567	1084568.5	2.2
S6C	A	DdlA-BRDT	DMSO	3978750	819249.7	2
S6C	A	DdlA-BRDT	DMSO	3901848.4	806509.4	2.2
S6C	A	DdlA-BRDT	DMSO	3703446.8	915067.7	2.1
S6C	A	DdlA-BRDT	20 µM BacPROTAC-4	3841400.6	1073553.3	2.5
S6C	A	DdlA-BRDT	20 µM BacPROTAC-4	2512184	677925.4	2.5
S6C	A	DdlA-BRDT	20 µM BacPROTAC-4	2567377.9	698711.3	2.1
S6D	A	BRDT-ThrC	DMSO	1844634.9	1242189.2	3.3
S6D	A	BRDT-ThrC	DMSO	1702284.9	1274101.1	3.6
S6D	A	BRDT-ThrC	20 µM BacPROTAC-3	1344523.5	1259378.1	3.4
S6D	A	BRDT-ThrC	20 µM BacPROTAC-3	1250201.7	1119118.9	2.9
S6D	B	BRDT-ThrC	DMSO	894002.5	645488	2.5
S6D	B	BRDT-ThrC	DMSO	865069.4	627068.8	1.8
S6D	B	BRDT-ThrC	20 µM BacPROTAC-3	504392.7	530727.1	1.8
S6D	B	BRDT-ThrC	20 µM BacPROTAC-3	611170.1	564502.5	2.4

Data S1. Original SDS PAGE gels, MIC plates and Wes data, Related to Figures 1,4,5,6 and STAR Methods

(A) Uncropped Coomassie stained SDS-PAGE gels and **(B)** uncropped MIC plates. The respective figures are indicated. **(C)** Representative Wes experiment measuring $BRDT_{BD1}$ and RpoB levels in *M. smegmatis* cells upon BacPROTAC treatment shown in **Figure 5B**. Exemplary electropherograms showing intensity of the chemiluminescent signal plotted against the apparent molecular weight detected using anti-BRDT and anti-RpoB antibodies. Applied chemical agents are indicated. (**D**) Bar chart showing quantification of detected RpoB peaks (loading control) from three independent experiments normalized to DMSO treatment (dark grey bar) and plotted as mean ± standard deviation. Quantification of the $BRDT_{BD1}$ peak is shown in **Figure 5B**. **(E)** Significance of *in vivo* degradation experiments monitored by Wes. Experimental variation of the normalized RpoB levels (green bar) serving as internal loading control in each experiment compared to the variation of normalized $BRDT_{BD1}$ levels (blue bar) showing that constant sample amounts were loaded in each experiment. Results from **Figures 5B, 5D**, **5E**, **S6C**, **S6D** are shown as gray bars. Experimental raw data is listed in **Table S4**.

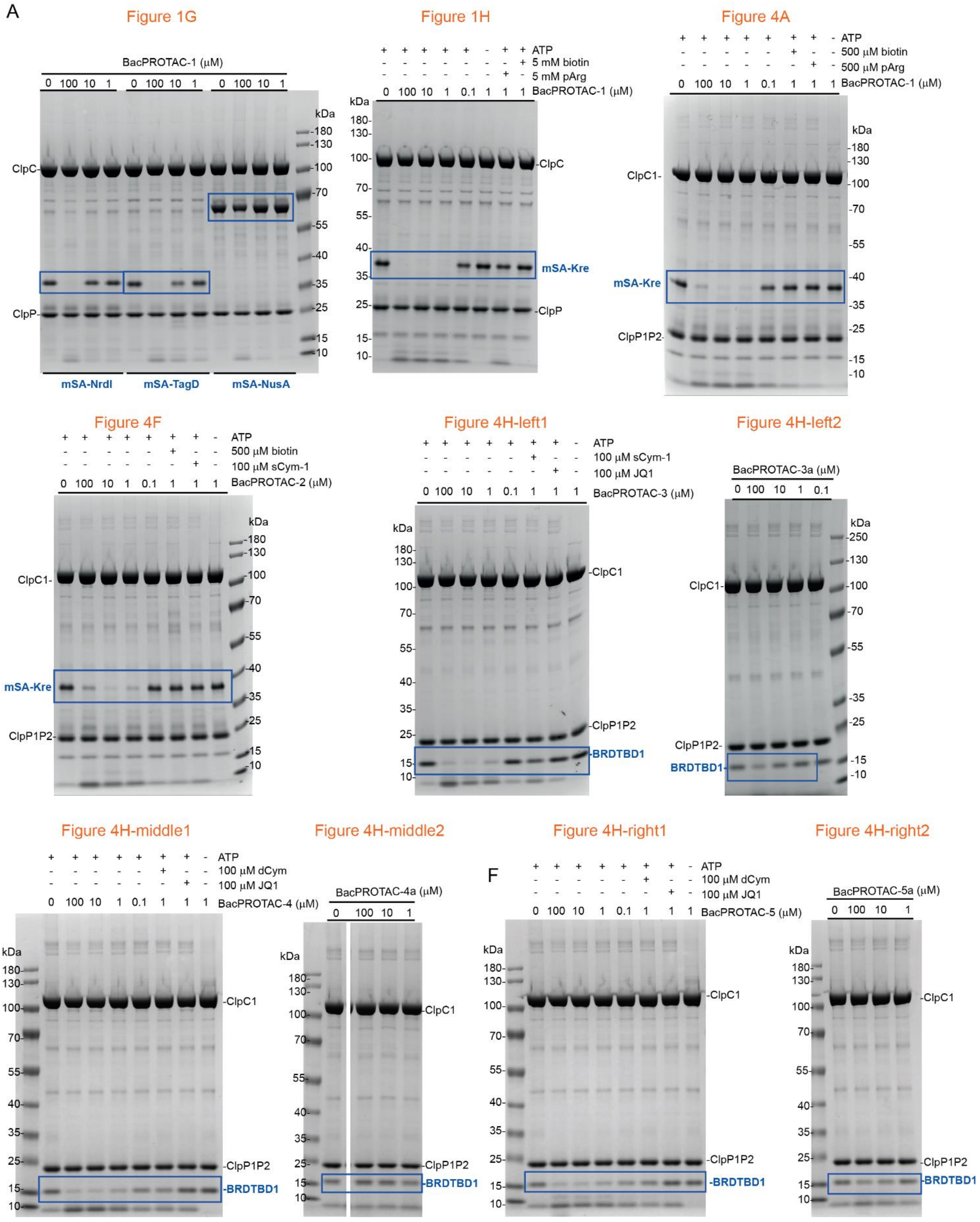
A
Figure 1G
BacPROTAC-1 (μM)
0 100 10 1 0 100 10 1 0 100 10 1
kDa
ClpC
ClpP
mSA-NrdI
mSA-TagD
mSA-NusA
Figure 1H
ATP
5 mM biotin
5 mM pArg
0 100 10 1 0.1 1 1 1 BacPROTAC-1 (μM)
ClpC
mSA-Kre
ClpP
Figure 4A
ATP
500 μM biotin
500 μM pArg
0 100 10 1 0.1 1 1 1 BacPROTAC-1 (μM)
ClpC1
mSA-Kre
ClpP1P2
Figure 4F
ATP
500 μM biotin
100 μM sCym-1
0 100 10 1 0.1 1 1 1 BacPROTAC-2 (μM)
ClpC1
mSA-Kre
ClpP1P2
Figure 4H-left1
ATP
100 μM sCym-1
100 μM JQ1
0 100 10 1 0.1 1 1 1 BacPROTAC-3 (μM)
ClpC1
ClpP1P2
BRDTBD1
Figure 4H-left2
BacPROTAC-3a (μM)
0 100 10 1 0.1
ClpC1
ClpP1P2
BRDTBD1
Figure 4H-middle1
ATP
100 μM dCym
100 μM JQ1
0 100 10 1 0.1 1 1 1 BacPROTAC-4 (μM)
ClpC1
ClpP1P2
BRDTBD1
Figure 4H-middle2
BacPROTAC-4a (μM)
0 100 10 1
ClpC1
ClpP1P2
BRDTBD1
Figure 4H-right1
F
ATP
100 μM dCym
100 μM JQ1
0 100 10 1 0.1 1 1 1 BacPROTAC-5 (μM)
ClpC1
ClpP1P2
BRDTBD1
Figure 4H-right2
BacPROTAC-5a (μM)
0 100 10 1
ClpC1
ClpP1P2
BRDTBD1

B

Figure S6A

Figure 6C

Figure 6C

Figure S6B

Figure 6D

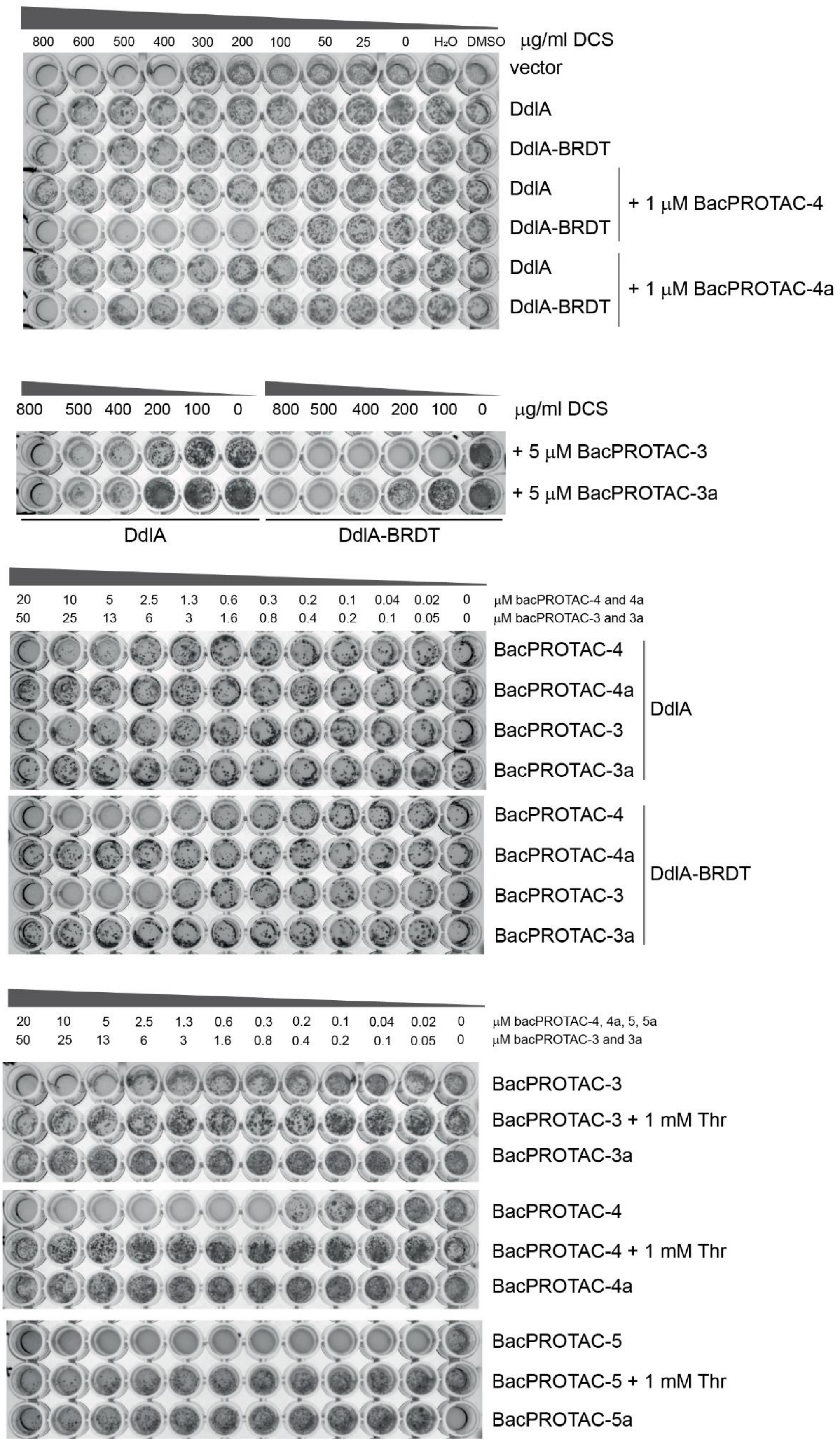

C

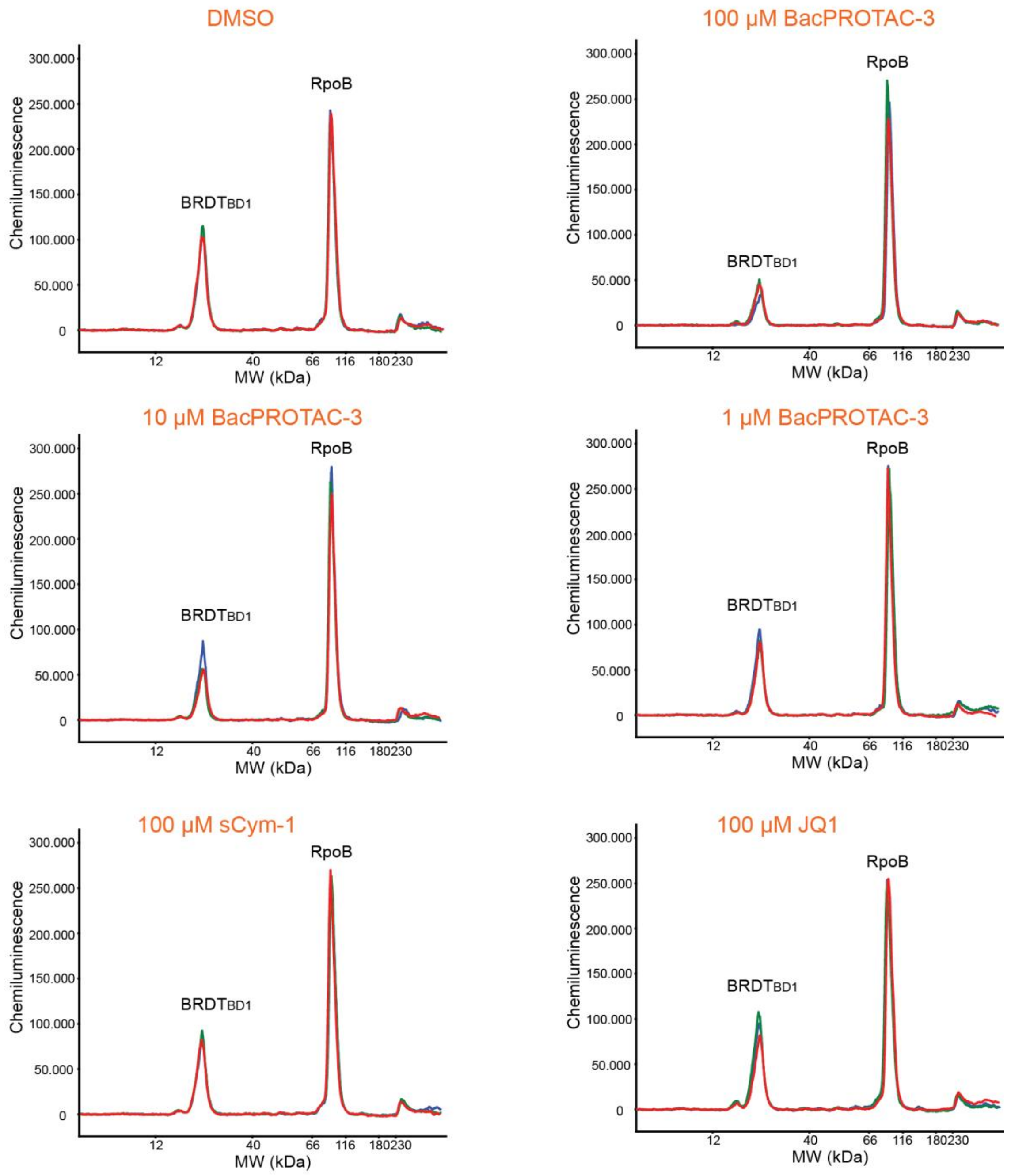
DMSO
100 µM BacPROTAC-3
10 µM BacPROTAC-3
1 µM BacPROTAC-3
100 µM sCym-1
100 µM JQ1
RpoB
BRDTBD1
Chemiluminescence
MW (kDa)
300.000
250.000
200.000
150.000
100.000
50.000
0
12
40
66
116
180
230

D

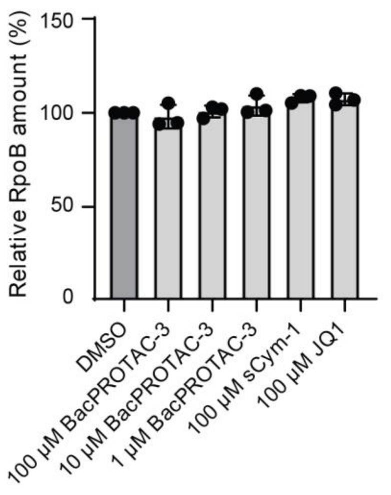
Relative RpoB amount (%)
150
100
50
0
DMSO
100 µM BacPROTAC-3
10 µM BacPROTAC-3
1 µM BacPROTAC-3
100 µM sCym-1
100 µM JQ1

E

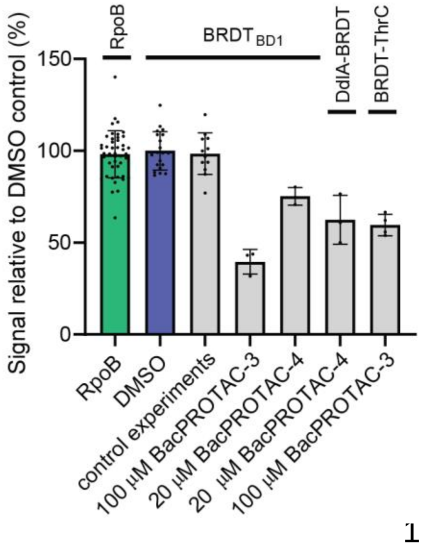
Signal relative to DMSO control (%)
RpoB
BRDTBD1
DdlA-BRDT
BRDT-ThrC
150
100
50
0
RpoB
DMSO
control experiments
100 µM BacPROTAC-3
20 µM BacPROTAC-4
20 µM BacPROTAC-4
100 µM BacPROTAC-3

Methods S1. Chemical synthesis of BacPROTACs, Related to STAR Methods

Chemical synthesis

Reagents and solvents. Reagents were purchased from Acros, Fluka, Merck, Novabiochem, Riedel de Haen, Labseeker, Iris or Sigma-Aldrich and were used without further purification. Anhydrous solvents were purchased in the highest available quality from the same suppliers.

Thin layer chromatography (TLC). TLC was performed on Merck aluminum pre-coated silica gel plates (20 × 20 cm, $60F_{254}$). Spots were detected using UV irradiation at 254 nm or by soaking the TLC plates with a developing solution (1.5 g $KMnO_4$, 10 g K_2CO_3 and 1.25 mL of 10% aq. NaOH in 200 mL water). Subsequently, the TLC plates were heated to visualize the respective spots. Eluents and R_f values are given in the particular experimental description.

Reversed-phase liquid chromatography electrospray ionization mass spectrometry (LC-MS). LC-MS analyses were performed on a LC-MS system from Thermo Scientific with an Eclipse XDB-C18 (5 µm) column from Agilent (peak detection at 210 nm) and a Thermo Scientific LCQ FleetTM ESI-Spectrometer. The following gradient program was applied: 0-1 min: 90 % water, 10 % acetonitrile + 0.1 % formic acid, 1-10 min: linear increase to 100 % acetonitrile + 0.1 % formic acid, 10-15 min: 100 % acetonitrile + 0.1% formic acid, flow rate 1 mL min^{-1}.

HPLC purifications. For HPLC purifications, a Prominence UFLC system from Shimadzu was used at a wavelength of 210 nm for peak detection. A reversed-phase column Luna 5 µm C18(2), 100 x 21.20 mm from Phenomenex was used at a flow rate 20 mL min^{-1}. As eluents, water with 0.1 % TFA and acetonitrile with 0.1 % TFA were used. The following gradient programs were applied: Purification of sCym-1. 0-5 min 30 % acetonitrile, 70 % water and + 0.1 % TFA, 5 – 25 min linear increase to 60 % acetonitrile, 40 % water and 0.1 % TFA. Purification of Tce-protected BacPROTAC-1. 0-5 min 30 % acetonitrile, 70 % water and + 0.1 % TFA, 5 – 25 min linear increase to 55 % acetonitrile, 45 % water and 0.1 % TFA. Purification of BacPROTAC-1. 0-5 min 2 % acetonitrile, 98 % water and + 0.1 % ammonia (30 % solution in water), 5 – 25 min linear increase to 18 % acetonitrile, 82 % water + 0.1 % ammonia (30 % solution in water). Purification of BacPROTAC-2. 0-5 min 30 % acetonitrile, 70 % water and + 0.1 % TFA, 5 – 25 min linear increase to 60 % acetonitrile, 40 % water and 0.1 % TFA.

Purification of BacPROTAC-3. 0-5 min 30 % acetonitrile, 70 % water and + 0.1 % TFA, 5 – 25 min linear increase to 60 % acetonitrile, 40 % water and 0.1 % TFA.

Nuclear magnetic resonance (NMR). NMR spectra were recorded on a Bruker Avance II 400 system (400 MHz). ^{1}H NMR spectra are reported in the following manner: chemical shifts (δ) in ppm calculated with reference to the residual signals of undeuterated solvent, multiplicity (s, singlet; d, doublet; t, triplet; dd, doublet of doublet; dt, doublet of triplet; m, multiplet; b, broad signal), coupling constants (J) in Hertz (Hz), and number of protons (H).

Synthesis of BacPROTAC-1

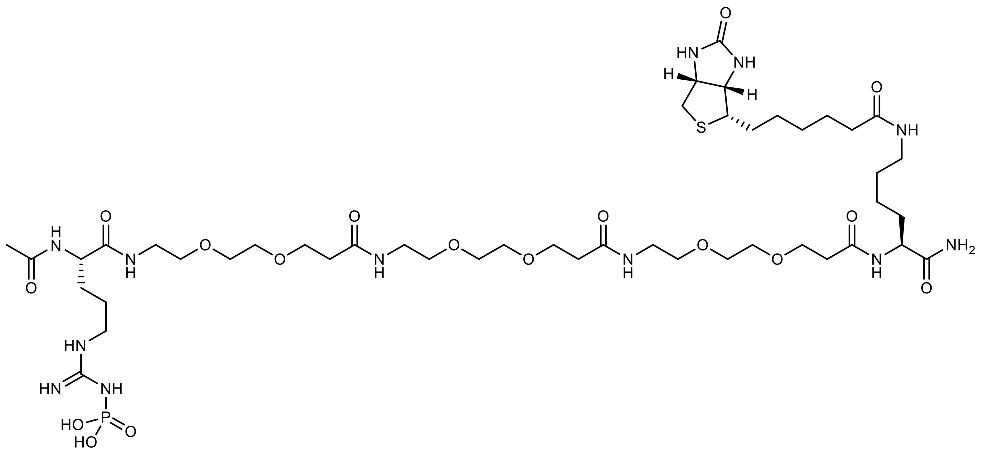

BacPROTAC-1 was synthesized *via* a solid phase peptide synthesis approach, following essentially synthesis protocols from reference (Hofmann et al., 2011):

1) Loading of Rink amide resin

Commercially available Fmoc-protected Rink amide resin (150 mg, 0.8 mmol g^{-1} initial loading) was suspended in a solution of DMF:piperidine (4:1, 10 mL) and shaken for 40 min at room temperature. The solution was removed and the remained resin was washed 2× with DMF, 2× with DCM and 2× with NMP. Fmoc-L-Lys(Biotin)-OH (286 mg, 0.48 mmol), HOBt monohydrate (74 mg, 0.48 mmol), HBTU (182 mg, 0.48 mmol) and DIPEA (123 µL, 0.72 mmol) were dissolved in NMP (5 mL). This solution was added to the resin and the resulting suspension was shaken for 4 h at room temperature. After removal of the solution, the remained resin was washed 2× with DMF, 2× with DCM and 2× with DMF. For capping, acetic anhydride (30 µL, 0.32 mmol) and DIPEA (200 µL) were dissolved in NMP (5 mL). This solution was added to the resin and the resulting suspension was shaken for 30 min at room temperature.

After removal of the solution, the remained resin was washed 2× with DMF, 2× with DCM and again 2× with DMF.

2) Solid phase amino acid assembly

The peptide was built up by application of generic solid phase peptide synthesis procedures:

Fmoc deprotection: A solution of DMF:piperidine (3:2, 5 mL) was added to the resin. The resulting suspension was shaken for 20 minutes at room temperature. The solution was removed and the cleavage step was repeated once more. The remained resin was washed 2× with DMF, 2× with DCM and 2× with DMF.

Coupling of Fmoc-protected amino acids: The corresponding Fmoc-protected amino acid (3 eq., these were either Fmoc-AEEP-OH or Fmoc-L-Arg(PO(OTce)$_2$-OH), HOBt monohydrate (3 eq.), HBTU (3 eq.) and DIPEA (3 eq.) were dissolved in DMF (5 mL). This solution was added to the resin and the resulting suspension was shaken for 2 hours at room temperature. The solution was removed and the remained resin was washed 2× with DMF, 2× with DCM and 2× with DMF.

3) N-terminal acylation

Acetic anhydride (30 µL) and DIPEA (100 µL) were dissolved in NMP (5 mL). This solution was then added to the resin and the resulting suspension was shaken for 1 h at room temperature. The solution was removed and the remained resin was washed 2× with DMF, 2× with DCM and 2× DMF.

4) Acidic cleavage and purification of the Tce-protected peptide

A solution of TFA:DCM (3:1, 5 mL) was added to the resin and the resulting suspension was shaken for 1 h at room temperature. The resin was filtered off and ice-cold diethyl ether (35 mL) was added to the filtrate and stored over night at -20 °C. The resulting suspension was centrifuged and the filtrate was decanted off. The residue was taken up in water:acetonitrile (4:1,15 mL) and purified by RP-HPLC with 0.1% TFA in water and 0.1% TFA in acetonitrile as the eluent system. Pooling and lyophilization of product containing fractions resulted in 121 mg (0.087 mmol, 73% yield based on initial resin loading) of the desired product as a white powder.

LC-MS (ESI): t_R = 5.93 min, *m/z* 1386.4 calcd. for $C_{49}H_{85}Cl_6N_{12}O_{17}PS$, found: 1389.6 $[M+H]^+$.

5) Hydrogenation and purification

The Tce-protected peptide (66 mg, 0.0475 mmol) was dissolved in a mixture of 100 mM NH_4HCO_3 buffer (pH 9, 10 mL) and EtOH (5 mL). Argon was bubbled through the solution for 15 min, followed by an addition of the Pd/C catalyst (25 mg). Next, hydrogen gas was bubbled through the resulting suspension for 4 h at room temperature. The catalyst was filtered off and the remaining filtrate was removed under reduced pressure. The residue was taken up in water:acetonitrile (19:1,10 mL) and purified by RP-HPLC using 0.1% ammonia in water and 0.1% ammonia in acetonitrile as the eluent system. Pooling and lyophilization of product containing fractions resulted in 16 mg (0.0142 mmol, 30 %) of the desired BacPROTAC-1 as a white powder and ammonium salt.

LC-MS (ESI): t_R = 4.01 min, *m/z* 1126.5 calcd. for $C_{45}H_{83}N_{12}O_{17}PS$, found: 1127.4 $[M+H]^+$.

HRMS (ESI): *m/z* 1127.5530 calcd. for $C_{45}H_{84}N_{12}O_{17}PS^+$, found: 1127.5490 $[M+H]^+$.

^1H NMR (400 MHz, D_2O): δ = 6.02-5.97 (m, 1H), 5.82-5.78 (m, 1H), 5.70-5.64 (m, 2H), 5.19 (t, J = 5.6 Hz, 6H), 5.06 (s, 12H), 5.03-4.97 (m, 6H), 4.81 (t, J = 5.1 Hz, 6H), 4.65-4.56 (m, 4H), 4.40 (dd, J = 5.0/13.0 Hz, 1H), 4.16 (t, J = 12.4 Hz, 1H), 3.99 (t, J = 5.9 Hz, 2H), 3.94 (t, J = 6.0 Hz, 4H), 3.65 (t, J = 7.1 Hz, 2H), 3.44 (s, 3H), 3.29-3.20 (m, 2H), 3.18-2.90 (m, 10H), 2.87-2.74 (m, 4H), 2.51 (d, J = 6.6 Hz, 1H).

Synthesis of BacPROTAC-1a

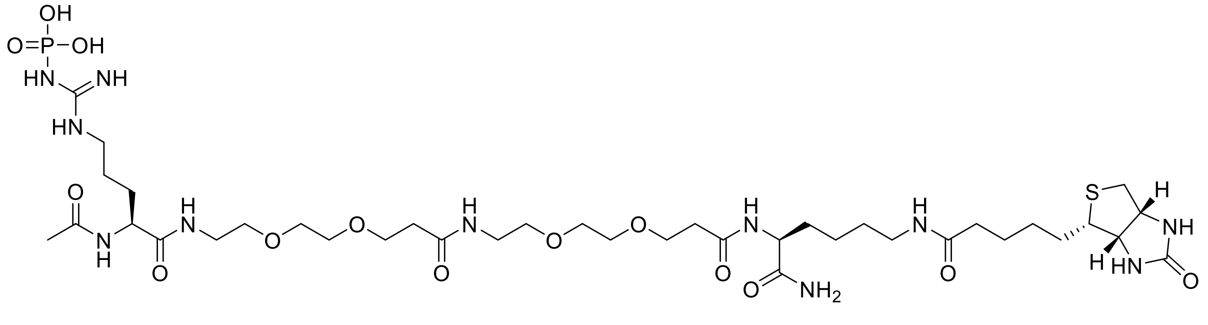

BacPROTAC-1a was synthesized analogously to the previously described solid phase peptide synthesis approach for BacPROTAC-1.

55 mg (0.046 mmol, 56% yield based on initial resin loading) of the protected peptide were obtained as a white powder.

LC-MS (ESI): t_R 6.01 min *m/z* 1227.3 calcd. for $C_{42}H_{72}Cl_6N_{10}O_{14}PS$, found: 1230.3 $[M+H]^+$

The hydrogenation and final purification by HPLC led to 9 mg (0.009 mmol, 20 %) of the desired product BacPROTAC-1b as a white powder.

LC-MS (ESI): t_R = 3.92 min, *m/z* 967.5 calcd. for $C_{38}H_{70}N_{11}O_{14}PS$, found: 968.3 $[M+H]^+$.

HRMS (ESI): *m/z* 968.4634 calcd. for $C_{38}H_{71}N_{11}O_{14}PS^+$, found: 968.4628 $[M+H]^+$.

^1H NMR (400 MHz, D_2O): δ = 4.34-4.29 (m, 2H), 4.15 (t, *J* = 6.3 Hz, 4H), 3.68 (q, *J* = 5.4 Hz, 5H), 3.56 (s, 9H), 3.52-3.47 (m, 5H), 3.32-3.27 (m, 4H), 3.24-3.19 (m, 2H), 3.16-3.05 (m, 5H), 2.91 (s, 1H), 2.89-2.84 (m, 1H), 2.76 (d, *J* = 13.1 Hz, 1H), 2.61 (s, 1H), 2.50 (t, *J* = 5.7 Hz, 2H), 2.44 (t, *J* = 6.1 Hz, 2H), 2.16 (q, *J* = 8.0 Hz, 2H), 1.94 (s, 3H), 1.79-1.69 (m, 3H), 1.67-1.51 (m, 7H), 1.48-1.39 (m, 4H), 1.34-1.26 (m, 3H).

Synthesis of BacPROTAC-1b

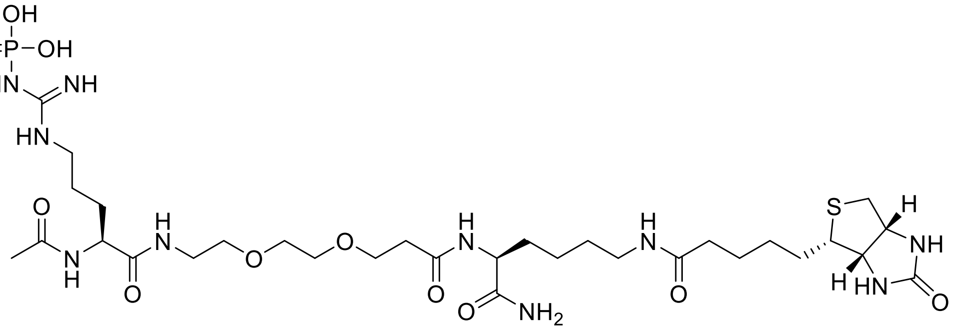

BacPROTAC-1b was synthesized analogously to the previously described solid phase peptide synthesis approach for BacPROTAC-1.

45 mg (0.042 mmol, 53% yield based on initial resin loading) of the protected peptide were obtained as a white powder.

LC-MS (ESI): t_R 6.12 min *m/z* 808.4 calcd. for $C_{35}H_{59}Cl_6N_{10}O_{11}PS$, found: 1071.2 $[M+H]^+$.

The hydrogenation and final purification by HPLC led to 12 mg (0.015 mmol, 36 %) of the desired product BacPROTAC-1b as a white powder.

LC-MS (ESI): t_R = 3.83 min, *m/z* 808.4 calcd. for $C_{31}H_{57}N_{10}O_{11}PS$, found: 809.3 $[M+H]^+$.

HRMS (ESI): *m/z* 809.3739 calcd. for $C_{31}H_{58}N_{10}O_{11}PS^{+}$, found: 809.3730 $[M+H]^{+}$.

^{1}H NMR (400 MHz, D_2O): δ = 4.52-4.48 (m, 4H), 4.33-4.29 (m, 2H), 4.18-4.13 (m, 3H), 3.69 (t, *J* = 5.8 Hz, 3H), 3.55 (s, 4H), 3.49 (t, *J* = 5.2 Hz, 2H), 3.29 (t, *J* = 5.3 Hz, 2H), 3.24 (s, 2H), 3.21 (s, 5H), 3.14-3.06 (m, 4H), 2.89 (dd, *J* = 4.7/12.9 Hz, 1H), 2.68 (d, *J* = 13.2 Hz, 1H), 2.49 (t, *J* = 5.5 Hz, 2H), 2.17-2.11 (m, 2H), 1.93 (s, 3H), 1.78-1.69 (m, 2H), 1.67-1.51 (m, 7H), 1.47-1.39 (m, 3H), 1.35-1.25 (m, 4H).

Synthesis of BacPROTAC-1c

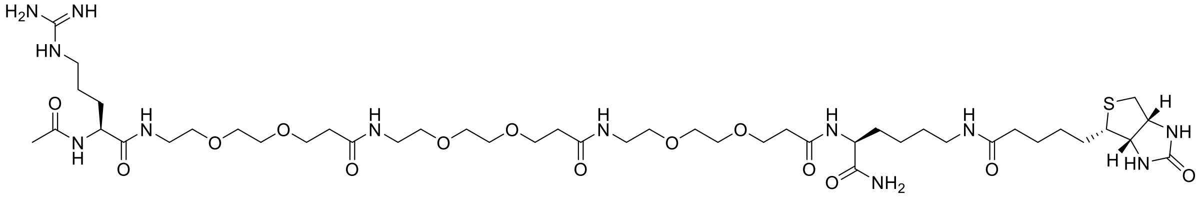

BacPROTAC-1c was synthesized *via* a solid phase peptide synthesis approach.

1) Loading of Rink amide resin

Commercially available Fmoc-protected Rink amide resin (50 mg, 0.8 mmol g^{-1} initial loading) was suspended in a solution of DMF:piperidine (4:1, 10 mL) and shaken for 40 min at room temperature. The solution was removed and the remained resin was washed 2× with DMF, 2× with DCM and 2× with NMP. Fmoc-L-Lys(Biotin)-OH (48 mg, 0.08 mmol) and DIPEA (28 µL, 0.16 mmol) were dissolved in NMP (5 mL). This solution was added to the resin and the resulting suspension was shaken for 16 h at room temperature. After removal of the solution, the remained resin was washed 2× with DMF, 2× with DCM and 2× with DMF. For capping, acetic anhydride (15 µL, 0.16 mmol) and DIPEA (100 µL) were dissolved in NMP (5 mL). This solution was added to the resin and the resulting suspension was shaken for 30 min at room temperature. After removal of the solution, the remained resin was washed 2× with DMF, 2× with DCM and again 2× with DMF.

2) Solid phase amino acid assembly

The peptide was built up by application of generic solid phase peptide synthesis procedures:

Fmoc deprotection: A solution of DMF:piperidine (3:2, 5 mL) was added to the resin. The resulting suspension was shaken for 20 minutes at room temperature. The solution was removed and the cleavage step was repeated once more. The remained resin was washed 2× with DMF, 2× with DCM and 2× with DMF.

Coupling of Fmoc-protected amino acids: The corresponding Fmoc-protected amino acid (3 eq., these were either Fmoc-AEEP-OH or Fmoc-L-Arg(Pbf)-OH), HOBt monohydrate (3 eq.), HBTU (3 eq.) and DIPEA (3 eq.) were dissolved in DMF (5 mL). This solution was added to the resin and the resulting suspension was shaken for 2 hours at room temperature. The solution was removed and the remained resin was washed 2× with DMF, 2× with DCM and 2× with DMF.

3) N-terminal acylation

Acetic anhydride (15 µL) and DIPEA (100 µL) were dissolved in NMP (5 mL). This solution was then added to the resin and the resulting suspension was shaken for 1 h at room temperature. The solution was removed and the remained resin was washed 2× with DMF, 2× with DCM and 2× DMF.

4) Acidic cleavage and purification of the crude peptide

A solution of TFA:DCM (3:1, 5 mL) was added to the resin and the resulting suspension was shaken for 1 h at room temperature. The resin was filtered off and ice-cold diethyl ether (35 mL) was added to the filtrate and stored over night at -20 °C. The resulting suspension was centrifuged and the filtrate was decanted off. The residue was taken up in water:acetonitrile (4:1,5 mL) and purified by RP-HPLC with 0.1% TFA in water and 0.1% TFA in acetonitrile as the eluent system. Pooling and lyophilization of product containing fractions resulted in 10 mg (0.009 mmol, 24% yield based on initial resin loading) of the desired product as a white powder.

LC-MS (ESI): t_R = 4.53 min, *m/z* 1046.6 calcd. for $C_{45}H_{82}N_{12}O_{14}S$, found: 1047.5 $[M+H]^+$.

^1H NMR (400 MHz, MeOD): δ = 4.51 (q, *J* = 4.2 Hz, 1H), 4.38 (t, *J* = 6.7 Hz, 1H), 4.35-4.30 (m, 2H), 3.81-3.73 (m, 6H), 3.66-3.60 (m, 12H), 3.56 (t, *J* = 5.4 Hz, 6H), 3.42-3.36 (m, 6H), 3.26-3.17 (m, 5H), 2.95 (dd, *J* = 4.9/12.8 Hz, 1H), 2.72 (d, *J* = 12.9 Hz, 1H), 2.60-2.52 (m, 2H), 2.49 (t, *J* = 6.2 Hz, 4H), 2.22 (t, *J* = 7.4 Hz, 2H), 2.02 (s, 3H), 1.91-1.81 (m, 2H), 1.78-1.59 (m, 8H), 1.57-1.50 (m, 2H), 1.49-1.40 (m, 4H).

Synthesis of BacPROTAC-2

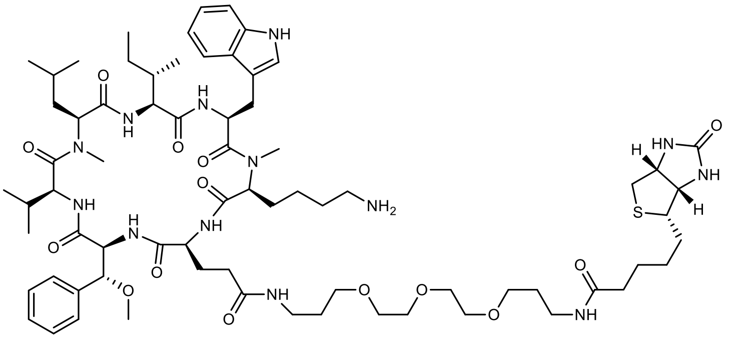

BacPROTAC-2 was synthesized *via* a solid phase peptide synthesis approach. Prior to SPPS, two non-commercial amino acid building blocks had to be synthesized.

* *Chemical synthesis of Fmoc-L-Phe(3R-MeO)-OH*

The synthesis of Fmoc-L-Phe(*3R*-MeO)-OH was achieved in several steps:

1) Chemical synthesis of Phth-L-Phe(3*R*-OH)-OH

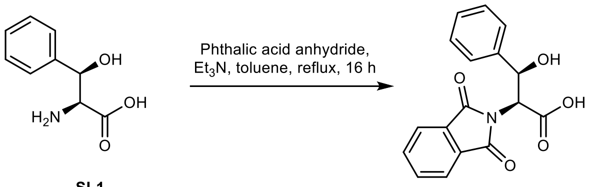

SI-1

SI-2

L-*threo*-Phenylserine (**SI-1**, 1 g, 5.52 mmol) was suspended in toluene (10 mL). Et_3N (76 µL, 0.55 mmol) and phthalic acid anhydride (815 mg, 5.52 mmol) were added and resulting suspension was refluxed for 16 hours. The solvent was evaporated under reduced pressure and the residue was purified by silica gel chromatography using DCM/MeOH/formic acid (9:1:0.01) as the eluent system. Product containing fractions were pooled and evaporated to dryness, yielding 1.6 g (5.14 mmol, 93 %) of the desired product **SI-2**.

TLC (DCM/MeOH/formic acid = 9:1:0.01): R_f = 0.4.

LC-MS (ESI): t_R = 7.57 min, *m/z* 311.1 calcd. for $C_{17}H_{13}NO_5$, found: 311.8 $[M+H]^+$.

^{1}H NMR (400 MHz, DMSO-d6): δ = 7.97-7.85 (m, 4H), 7.44 (d, *J* = 7.4 Hz, 2H), 7.34 (t, *J* = 7.3 Hz, 2H), 7.26 (t, *J* = 7.2 Hz, 1H), 5.32 (d, *J* = 8.9 Hz, 1H), 5.00 (d, *J* = 9.0 Hz, 1H).

2) Chemical synthesis of Phth-L-Phe(3*R*-MeO)-OMe

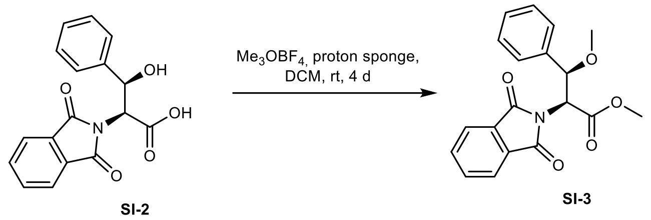

SI-2 (1.6 g, 5.14 mmol) was dissolved in dry DCM (50 mL) under an argon atmosphere. Proton sponge (11 g, 51.4 mmol) and Me_3OBF_4 (7.6 g, 51.4 mmol) were added and the resulting solution was stirred for 4 days at room temperature. The solvent was evaporated under reduced pressure to dryness and the residue was purified with silica gel column chromatography using 50-100% EtOAc in cyclohexane as the eluent system. Product containing fractions were pooled and evaporated to dryness, yielding 1.02 g (3.01 mmol, 59%) of the desired product **SI-3**.

TLC (EtOAc): R_f = 0.8.

LC-MS (ESI): t_R = 9.94 min, *m/z* 339.1 calcd. for $C_{19}H_{17}NO_5$, found: 339.7 $[M+H]^+$.

^{1}*H NMR* (400 MHz, DMSO-d6): δ = 7.98-7.89 (m, 4H), 7.44-7.31 (m, 5H), 5.11 (d, *J* = 8.7 Hz, 1H), 5.01 (d, *J* = 8.8 Hz, 1H), 3.53 (s, 3H), 2.98 (s, 3H).

3) Chemical synthesis of Fmoc-L-Phe(3*R*-MeO)-OMe

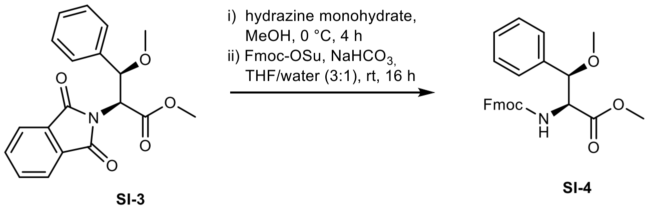

SI-3 (1.02 g, 3.01 mmol) was dissolved in MeOH (10 mL) and the resulting solution was cooled down to 0 °C. Hydrazine monohydrate (435 µL, 13.5 mmol) was added and the resulting mixture was stirred for 4 hours at 0 °C. The solvent was evaporated under reduced pressure, yielding an oily residue. The residue was re-dissolved in a mixture of THF/water (3:1, 10 mL). Fmoc-OSu (2.53 g, 7.5 mmol) and in a minimal amount water pre-dissolved $NaHCO_3$ (1.26 g, 15 mmol) were added and the resulting mixture was stirred for 16 hours at room temperature. After removal of the solvent in vacuum, the residue was purified by silica gel column chromatography using 10-16% EtOAc in cyclohexane as the eluent system. Product containing fractions were pooled

and evaporated to dryness, thereby yielding 456 mg (1.06 mmol, 35%) of the desired product **SI-4**.

TLC (cyclohexane/EtOAc = 3:1): R_f = 0.4.

LC-MS (ESI): t_R = 11.20 min, *m/z* 431.2 calcd. for $C_{26}H_{25}NO_5$, found: 431.7 $[M+H]^+$.

^1H NMR (400 MHz, DMSO-d6): δ = 7.89 (d, *J* = 7.1 Hz, 2H), 7.71 (q, *J* = 7.8 Hz, 2H), 7.45-7.27 (m, 10H), 4.72 (d, *J* = 4.8 Hz, 1H), 4.35 (q, *J* = 4.6 Hz, 1H), 4.13 (s, 3H), 3.57 (s, 3H), 3.17 (s, 3H).

4) Chemical synthesis of Fmoc-L-Phe(3*R*-MeO)-OH

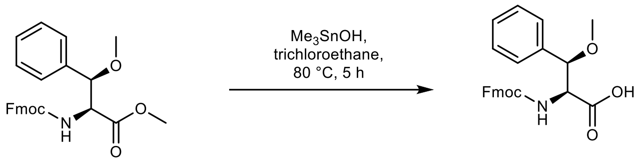

SI-4 SI-5

To prevent concomitant cleavage of the Fmoc protecting group during ester hydrolysis, we used the Nicolaou protocol for methyl ester deprotection (Nicolaou et al., 2005). To this end, **SI-4** (456 mg, 1.06 mmol) and Me_3SnOH (955 mg, 5.28 mmol) were dissolved in trichloroethane (10 mL). The resulting mixture was heated up for 5 h to 80 °C. The reaction was quenched by addition of aq. 5% $KHSO_4$. The organic phase was separated, dried over $MgSO_4$ and filtrated. The solvent was removed under reduced pressure to dryness and the residue was subjected to silica gel column chromatography, using 16-50% EtOAc in cyclohexane (acidified with 0.1% formic acid) as the eluent system. Product containing fractions were pooled and evaporated to dryness, yielding 263 mg (0.63 mmol, 60%) of the desired product **SI-5**.

TLC (EtOAc:cyclohexane:formic acid = 1:1:0.01): R_f = 0.4.

LC-MS (ESI): t_R = 10.12 min, *m/z* 417.1 calcd. for $C_{25}H_{23}NO_5$, found: 417.8 $[M+H]^+$.

^1H NMR (400 MHz, DMSO-d6): δ = 7.87 (d, *J* = 7.3, 2H), 7.76-7.66 (m, 3H), 7.49-7.20 (m, 9H), 4.78 (d, *J* = 3.7 Hz, 1H), 4.30 (q, *J* = 4.1 Hz, 1H), 4.14-4.05 (m, 3H), 3.18 (s, 3H).

** Chemical synthesis of Fmoc-L-Glu(TOTA-Biotin)-OH*

The chemical synthesis of Fmoc-L-Glu(TOTA-Biotin)-OH was achieved in several steps:

1) Chemical synthesis of Fmoc-L-Glu(TOTA-Biotin-O*t*Bu

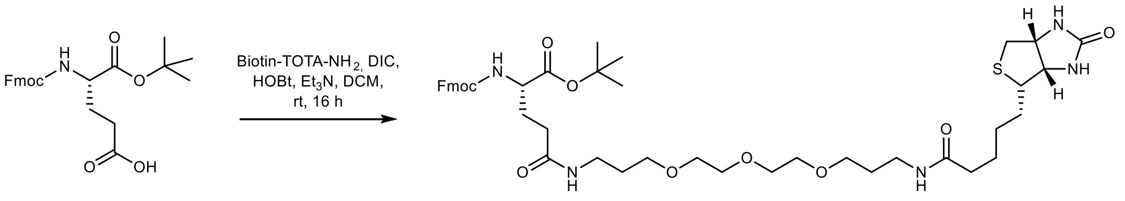

SI-6 **SI-7**

Fmoc-L-Glu-O*t*Bu (**SI-6**, 425 mg, 1 mmol) and Biotin-TOTA-NH_2 (670 mg, 1.5 mmol) were dissolved in DCM (10 mL). HOBt monohydrate (306 mg, 2 mmol), DIC (309 µL, 2 mmol) and triethylamine (277 µL, 2 mmmol) were added. The resulting solution was stirred at room temperature for 16 hours. Subsequently, the organic phase was washed with aq. 5% $NaHCO_3$ dried over $MgSO_4$ and reduced to dryness under reduced pressure. The resulting residue was purified by silica gel column chromatography, using 5 % to 25 % methanol in DCM as eluent the system. Product containing fractions were pooled and evaporated to dryness, thereby yielding 646 mg (0.76 mmol, 76%) of the desired product **SI-7** as a white solid.

TLC (DCM/MeOH = 9:1): R_f = 0.3.

LC-MS (ESI): t_R = 8.56 min, *m/z* 853.4 calcd. for $C_{44}H_{63}N_5O_{10}S$, found: 854.1 $[M+H]^+$.

^1H *NMR* (400 MHz, DMSO-d6): δ = 7.90 (d, *J* = 7.7 Hz, 2H), 7.81 (t, *J* = 5.5 Hz, 1H), 7.77-7.70 (m, 3H), 7.67 (d, *J* = 8.2 Hz, 1H), 7.43 (t, *J* = 7.4 Hz, 2H), 7.34 (t, *J* = 7.5 Hz, 2H), 6.24 (s, 1H), 6.35 (s, 1H), 4.34-4.19 (m, 4H), 4.15-4.11 (m, 1H), 3.91-3.85 (m, 1H), 3.53-3.44 (m, 8H), 3.38 (t, *J* = 6.0 Hz, 4H), 3.12-3.03 (m, 5H), 2.81 (dd, *J* = 5.2, 12.2 Hz, 1H), 2.58 (d, *J* = 12.4 Hz, 1H), 2.17 (t, *J* = 7.5 Hz, 2H), 2.05 (t, *J* = 7.3 Hz, 2H), 1.99-1.89 (m, 1H), 1.81-1.72 (m, 1H), 1.65-1.57 (m, 5H), 1.53-1.44 (m, 3H), 1.39 (s, 9H), 1.34-1.23 (m, 2H).

2) Chemical synthesis of Fmoc-L-Glu(TOTA-Biotin)-OH

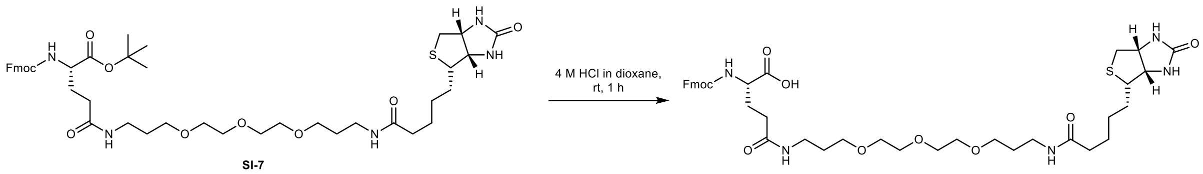

SI-7 **SI-8**

SI-7 (300 mg, 0.35 mmol) was dissolved in 4 M HCl in dioxane (4 mL) and the resulting mixture was stirred for one hour at room temperature. The solvent was removed under reduced pressure and the resulting residue was dried at high vacuum to obtain 291 mg (0.35 mmol, >98%) of the desired product **SI-8** as a white powder.

TLC (DCM/MeOH/formic acid = 3:1:0.02): R_f = 0.2.

LC-MS (ESI): t_R = 7.21 min, *m/z* 797.4 calcd. for $C_{44}H_{63}N_5O_{10}S$, found: 798.2 $[M+H]^+$.

^{1}H NMR (400 MHz, DMSO-d6): δ = 7.90 (d, *J* = 7.7 Hz, 2H), 7.86-7.77 (m, 2H), 7.73 (d, *J* = 7.3 Hz, 2H), 7.67 (d, *J* = 8.1 Hz, 1H), 7.43 (t, *J* = 7.3 Hz, 2H), 7.34 (d, *J* = 7.3 Hz, 2H), 4.34-4.18 (m, 4H), 4.15-4.10 (m, 1H), 3.98-3.91 (m, 1H), 3.75-3.64 (m, 3H), 3.52-3.43 (m, 8H), 3.39 (t, *J* = 6.0 Hz, 4H), 3.13-3.02 (m, 5H), 2.81 (dd, *J* = 5.1, 12.4 Hz, 1H), 2.59 (d, *J* = 12.4 Hz, 1H), 2.17 (t, *J* = 7.5 Hz, 2H), 2.06 (t, *J* = 7.1 Hz, 2H), 2.01-1.93 (m, 1H), 1.84-1.72 (m, 1H), 1.65-1.55 (m, 5H), 1.53-1.41 (m, 3H), 1.34-1.22 (m, 2H).

** Solid phase peptide synthesis of BacPROTAC-2*

BacPROTAC-2 was synthesized *via* a solid phase peptide synthesis approach. It entailed the following steps:

1) Loading of the resin

Fmoc-L-Phe(3*R*-MeO)-OH (**SI-5**, 105 mg, 0.25 mmol) and DIPEA (85 µL, 0.5 mmol) were dissolved in DMF (5 mL). The resulting solution was added to 2-chlorotrityl resin (200 mg, 1.6 mmol g^{-1} initial loading). The resulting suspension was shaken for 16 h at room temperature. After removal of the solution, the resin was washed 2× with DMF, 2× with DCM and 2× with DMF. For capping, a mixture of DCM:MeOH:DIPEA (3:1:0.1, 4 mL) was added to the resin and the resulting suspension was shaken for 30 minutes at room temperature. The solvent was removed and the remained resin was washed again 2× with DMF, 2× with DCM and 2× with DMF.

2) Solid phase amino acid assembly

The peptide was built up by application of generic solid phase peptide synthesis procedures:

Fmoc deprotection: A solution of DMF:piperidine (3:2, 5 mL) was added to the resin. The resulting suspension was shaken for 20 minutes at room temperature. The solution was removed and the cleavage step was repeated once more. The remained resin was washed 2× with DMF, 2× with DCM and 2× with DMF.

Coupling of Fmoc-protected amino acids: The corresponding Fmoc-protected amino acid (3 eq.), HOBt monohydrate (3 eq.), HBTU (3 eq.) and DIPEA (3 eq.) were dissolved in DMF (5 mL). This solution was added to the resin and the resulting suspension was shaken for 2 hours at room temperature. The solution was removed and the remained resin was washed 2× with DMF, 2× with DCM and 2× with DMF.

Modified coupling procedure for couplings on *N*-methyl amino acids: The corresponding Fmoc-protected amino acid (3 eq.), 2-Bromo-1-ethyl-pyridinium tetrafluoroborate (3 eq.) and DIPEA (6 eq.) were dissolved in DMF (5 mL). This solution was added to the resin and the resulting suspension was shaken at room temperature for 4 hours. The solution was removed and the remained resin was washed 2× with DMF, 2× with DCM and 2× with DMF.

3) Cleavage from the resin

A mixture of DCM:hexafluoroisopropanol (3:1, 5 mL) was added to the resin. The resulting suspension was shaken at room temperature for 20 min. The cleavage solution was removed from the resin and transferred into a flask. The resin was washed 3× with DCM (2 mL) and the washing solutions were combined with the cleavage solution. The resulting solution was evaporated under reduced pressure to dryness to yield 269 mg (0.167 mmol, 67 %) of the desired product that was used in the next step without further purification.

LC-MS (ESI): t_R = 8.26 min, *m/z* 1619.9 calcd. for $C_{81}H_{129}N_{13}O_{19}S$, found: 1620.6 $[M+H]^+$.

4) In solution cyclization

This peptide (269 mg, 0.167 mmol) was dissolved in DCM (183 mL, final concentration: 0.75 mM). Triethylamine (57 µL, 0.411 mmol), HOAt (56 mg, 0.411 mmol) and DIC (64 µL, 0.411 mmol) were added. The resulting solution was stirred at room temperature for 16 hours. Aq. 5% $NaHCO_3$ (100 mL) was added, the organic phase was separated, dried over $MgSO_4$ and the organic phase was removed under

reduced pressure to obtain 169 mg (0.105 mmol, 63 %) of the cyclized peptide which was used in the next step without further purification.

LC-MS (ESI): t_R = 11.52 min, *m/z* 1601.9 calcd. for $C_{81}H_{127}N_{13}O_{18}S$, found: 1602.3 $[M+H]^+$.

5) Protecting group cleavage and purification

This cyclized peptide (70 mg, 0.043 mmol) was dissolved in a TFA:DCM (3:1, 10 mL) mixture and the resulting solution was stirred at room temperature for 2 h. The solvent was removed under reduced pressure and the residue was re-dissolved in DCM (25 mL). The organic layer was washed with saturated aq. $NaHCO_3$ and brine and dried over $MgSO_4$. After removal of the solvent by evaporation under reduced pressure, the residue was dried in high vacuum and purified by RP-HPLC with 0.1% TFA in water and 0.1% TFA in acetonitrile as eluent systems. Product containing fractions were pooled and lyophilized, thereby yielding 8 mg (0.006 mmol, 14 %) of BacPROTAC-2 as a white powder.

LC-MS (ESI): t_R = 7.38 min, *m/z* 1401.8 calcd. for $C_{71}H_{111}N_{13}O_{14}S$, found: 1403.4 $[M+H]^+$.

HRMS (ESI): *m/z* 1402.8167 calcd. for $C_{71}H_{112}N_{13}O_{14}S^+$, found: 1402.8127 $[M+H]^+$.

^{1}H NMR (400 MHz, DMSO-d6): δ = 11.10 (s, 1H), 9.64 (s, 1H), 9.32 (d, J = 7.9 Hz, 1H), 8.84 (d, J = 10.2 Hz, 1H), 8.18 (d, J = 8.5 Hz, 1H), 7.93 (t, J = 5.4 Hz, 1H), 7.76-7.65 (m, 6H), 7.62 (d, J = 7.7 Hz, 1H), 7.58 (d, J = 8.1 Hz, 1H), 7.46-7.36 (m, 7H), 7.28 (t, J = 7.4 Hz, 1H), 7.18 (t, J = 7.6 Hz, 1H), 6.60 (s, 1H), 6.54 (s, 1H), 5.33 (d, J = 10.3 Hz, 1H), 5.07 (d, J = 7.6 HZ, 2H), 5.01-4.94 (m, 1H), 4.64-4.54 (m, 2H), 4.50 (t, J = 6.4 Hz, 1H), 4.45-4.30 (m, 1H), 4.34-4.23 (m, 3H), 3.32-3.22 (m, 8H), 3.18 (s, 3H), 3.01 (dd, J = 5.1/12.3 Hz, 1H), 2.89 (s, 3H), 2.60 (s, 3H), 2.53-2.39 (m, 2H), 2.23 (t, J = 7.4 Hz, 3H), 2.05-1.96 (m, 1H), 1.80 (t, J = 6.4 Hz, 6H), 1.74-1.57 (m, 10H), 1.53-1.41 (m, 6H), 1.37-1.26 (m, 3H), 1.22 (d, J = 6.6 Hz, 3H), 1.12 (d, J = 6.2 Hz, 7H), 1.08-0.96 (m, 16H).

Synthesis of BacPROTAC-3

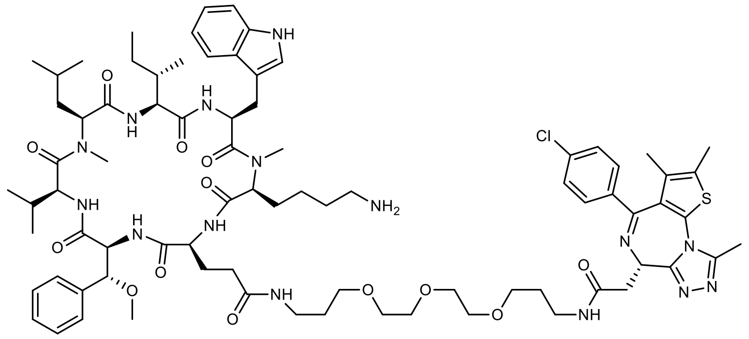

BacPROTAC-3 was synthesized *via* the previously described solid phase peptide synthesis approach. Prior to SPPS, one further non-commercial amino acid building block was synthesized.

** Chemical synthesis of Fmoc-L-Glu(TOTA-JQ1)-OH*

1) Chemical synthesis of Fmoc-L-Glu(TOTA-JQ1)-O*t*Bu

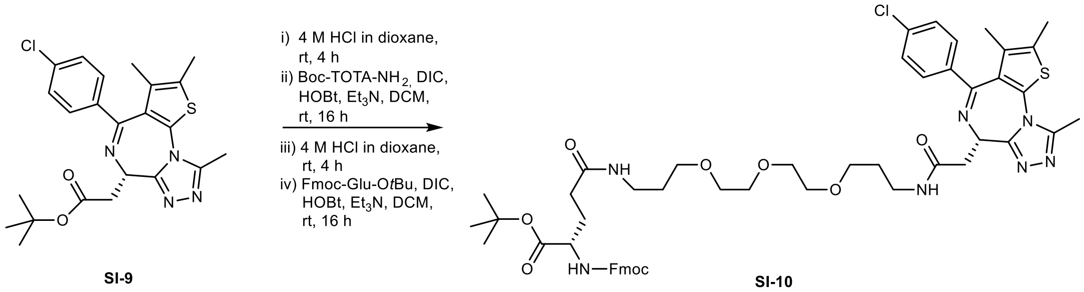

SI-9 **SI-10**

(+)-JQ1 (**SI-9**, 457 mg, 1 mmol) was dissolved in 4 M HCl in 1,4-dioxane (10 mL). The resulting solution was stirred for 4 hours at room temperature. The solvent was evaporated under reduced pressure and the resulting residue was used in the next step without further purification.

The obtained residue was dissolved in DCM (10 mL). Et_3N (693 µL, 5 mmol), HOBt monohydrate (306 mg, 2 mmol), DIC (310 µL, 2 mmol) and Boc-TOTA-NH_2 (481 mg, 1.5 mmol) were added. The resulting mixture was stirred at room temperature for 16 hours. The reaction mixture was diluted by addition saturated aq. $NaHCO_3$ (15 mL). The resulting phases were separated and the organic phase was washed with brine, followed by drying over $MgSO_4$. The solvent was removed under reduced pressure, yielding a residue that was used in the next step without further purification.

This residue was suspended in 4 M HCl in 1,4-dioxane (10 mL) and the resulting suspension was stirred for 1 hour. The solvent was removed by evaporation to dryness and the resulting residue was dissolved in DCM (10 mL), followed by addition of Et_3N (693 µL, 5 mmol), HOBt monohydrate (306 mg, 2 mmol), DIC (310 µL, 2 mmol) and Fmoc-L-Glu-O*t*Bu (638 mg, 1.5 mmol). The resulting mixture was stirred at room temperature for 16 hours. The organic phase was washed with brine, dried over $MgSO_4$ and evaporated under reduced pressure to dryness. The resulting residue was purified by silica gel column chromatography using 5-10% MeOH/DCM as the eluent system. Product containing fractions were pooled and evaporated to dryness, yielding 414 mg (0.41 mmol, 41%) of the desired product **SI-10**.

TLC (DCM/MeOH = 19:1): R_f = 0.2.

LC-MS (ESI): t_R = 10.87 min, *m/z* 1009.4 calcd. for $C_{53}H_{64}ClN_7O_9S$, found: 1010.3 $[M+H]^+$.

^1H NMR (400 MHz, $CDCl_3$): δ = 7.76 (d, *J* = 7.1 Hz, 2H), 7.64 (t, *J* = 8.2 Hz, 2H), 7.44-7.37 (m, 3H), 7.33 (t, *J* = 7.1 Hz, 3H), 4.92 (s, 1H), 4.35 (s, 1H), 4.24-4.10 (m, 2H), 3.71-3.54 (m, 12H), 3.52-3.44 (m, 1H), 3.37 (s, 3H), 2.94 (s, 2H), 2.42-2.24 (m, 4H), 2.18-2.07 (m, 1H), 2.05-1.95 (m, 1H), 1.84 (d, *J* = 5.6 Hz, 4H), 1.63 (d, *J* = 19.7 Hz, 3H), 1.46 (s, 9H).

2) Chemical synthesis of Fmoc-L-Glu(TOTA-JQ1)-OH

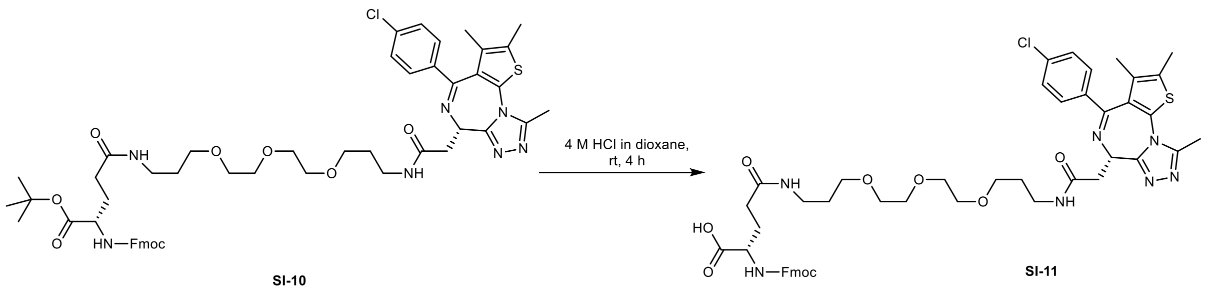

SI-10 SI-11

SI-10 (400 mg, 0.39 mmol) was dissolved in 4 M HCl in 1,4-dioxane (10 mL) and the resulting mixture was stirred for 2 hours at room temperature. The solution was evaporated under reduced pressure to dryness to yield 389 mg (0.41 mmol, >98%) of **SI-11**.

TLC (DCM/MeOH = 3:1): R_f = 0.3.

LC-MS (ESI): t_R = 9.24 min, *m/z* 953.3 calcd. for $C_{49}H_{56}ClN_7O_9S$, found: 954.5 $[M+H]^+$.

^{1}H NMR (400 MHz, $CDCl_3$): δ = 7.73 (d, *J* = 7.5 Hz, 2H), 7.66 (d, *J* = 8.2 Hz, 2H), 7.57-7.29 (m, 6H), 5.21 (s, 1H), 4.33-4.12 (m, 4H), 3.86 (s, 1H), 3.80-3.75 (m, 2H), 3.71 (s, 5H), 3.68-3.50 (m, 14H), 3.47-3.28 (m, 4H), 3.12 (s, 2H), 2.57 (s, 2H), 2.27 (s, 2H), 2.17-1.97 (m, 2H), 1.85 (s, 4H), 1.37 (s, 2H).

** Solid phase peptide synthesis of BacPROTAC-3*

1) Loading of the resin: **SI-5** (105 mg, 0.25 mmol) and DIPEA (85 µL, 0.5 mmol) were dissolved in DMF (5 mL) and this solution was added to 2-chlorotrityl resin (200 mg, 1.6 mmol g^{-1} initial loading). The resulting suspension was shaken for 16 h at room temperature. After removal of the solution, the resin was washed 2× with DMF, 2× with DCM and 2× with DMF. For capping, a mixture of DCM:MeOH:DIPEA (3:1:0.1, 4 mL) was added to the resin and the resulting suspension was shaken for 30 minutes at room temperature. The solvent was removed and the remained resin was washed again 2× with DMF, 2× with DCM and 2× with DMF.

2) Solid phase amino acid assembly and resin cleavage: The peptide was built up by application of generic solid phase peptide synthesis procedures:

Fmoc deprotection: A solution of DMF:piperidine (3:2, 5 mL) was added to the resin. The resulting suspension was shaken for 20 minutes at room temperature. The solution was removed and the cleavage step was repeated once more. The remained resin was washed 2× with DMF, 2× with DCM and 2× with DMF.

Coupling of Fmoc-protected amino acids: The corresponding Fmoc-protected amino acid (3 eq.), HOBt monohydrate (3 eq.), HBTU (3 eq.) and DIPEA (3 eq.) were dissolved in DMF (5 mL). This solution was added to the resin and the resulting suspension was shaken for 2 hours at room temperature. The solution was removed and the remained resin was washed 2× with DMF, 2× with DCM and 2× with DMF.

Modified coupling procedure for couplings on *N*-methyl amino acids: The corresponding Fmoc-protected amino acid (3 eq.), 2-Bromo-1-ethyl-pyridinium tetrafluoroborate (3 eq.) and DIPEA (6 eq.) were dissolved in DMF (5 mL). This solution was added to the resin and the resulting suspension was shaken at room temperature for 4 hours. The solution was removed and the remained resin was washed 2× with DMF, 2× with DCM and 2× with DMF.

Cleavage from resin: A mixture of DCM:hexafluoroisopropanol (3:1, 5 mL) was added to the resin. The resulting suspension was shaken at room temperature for 20 min. The cleavage solution was removed from the resin and transferred into a flask. The resin was washed 3× with DCM (2 mL) and the washing solutions were combined with the cleavage solution. The resulting solution was evaporated under reduced pressure to dryness. The residue was dried at high vacuum to obtain 229 mg (0.137 mmol, 55% based on initial resin loading) of a linear peptide that was used in the next step without further purification.

LC-MS (ESI): t_R = 8.35 min, *m/z* 1675.9 calcd. for $C_{85}H_{122}ClN_{15}O_{16}S$, found: 1677.2 $[M+H]^+$.

3) In solution cyclization

This linear peptide (229 mg, 0.137 mmol) was dissolved in DCM (183 mL, final concentration: 0.75 mM). Triethylamine (57 µL, 0.411 mmol), HOAt (56 mg, 0.411 mmol) and DIC (64 µL, 0.411 mmol) were added. The resulting solution was stirred at room temperature for 16 h. Aq. 5% $NaHCO_3$ (100 mL) was added and the organic layer was separated, dried over $MgSO_4$ and removed under reduced pressure to dryness to yield 146 mg (0.088 mmol, 64%) of a cyclized peptide that was used in the next step without further purification.

LC-MS (ESI): t_R = 11.36 min, *m/z* 1657.8 calcd. for $C_{85}H_{120}ClN_{15}O_{15}S$, found: 1658.3 $[M+H]^+$.

4) Final deprotection and purification

This cyclic peptide (146 mg, 0.088 mmol) was dissolved in TFA/DCM (3:1, 10 mL) and the resulting mixture was stirred for 2 hours at room temperature. The solvent was removed under reduced pressure and the residue was re-dissolved in DCM (25 mL). The organic layer was washed with saturated aq. $NaHCO_3$ and brine and dried over $MgSO_4$. After removal of the solvent by evaporation under reduced pressure, the residue was dried in high vacuum and purified by RP-HPLC with 0.1% TFA in water and 0.1% TFA in acetonitrile as eluent systems. Product containing fractions were pooled and lyophilized, thereby yielding 43 mg (0.027 mmol, 31%) of BacPROTAC-3 as a white powder.

LC-MS (ESI): t_R = 8.11 min, *m/z* 1557.8 calcd. for $C_{80}H_{112}ClN_{15}O_{13}S$, found: 1558.5 $[M+H]^+$.

HRMS (ESI): *m/z* 1558.8047 calcd. for $C_{80}H_{113}ClN_{15}O_{13}S^+$, found: 1558.7993 $[M+H]^+$.

^{1}H NMR (400 MHz, MeOD): δ = 9.39 (s, 1H), 9.22 (d, *J* = 8.4 Hz, 1H), 8.94 (d, *J* = 10.2 Hz, 1H), 7.93 (d, *J* = 9.3 Hz, 1H), 7.83 (d, *J* = 8.4 Hz, 1H), 7.61 (d, *J* = 7.4 Hz, 1H), 7.50-7.44 (m, 6H), 7.37-7.27 (m, 7H), 7.18 (t, *J* = 7.4 Hz, 1H), 7.09 (s, 3H), 5.38 (d, *J* = 10.1 Hz, 1H), 5.24-5.17 (m, 1H), 5.12 (d, *J* = 6.8 Hz, 1H), 4.69 (t, *J* = 7.3 Hz, 1H), 4.60 (t, *J* = 8.6 Hz, 1H), 4.49 (t, *J* = 10.9 Hz, 2H), 4.30 (q, *J* = 8.2 Hz, 1H), 3.67 (s, 6H), 3.62 (s, 8H), 3.54 (t, *J* = 5.9 Hz, 4H), 3.38 (s, 3H), 3.19 (s, 3H), 2.89 (s, 3H), 2.74 (s, 6H), 2.56 (s, 3H), 2.47 (s, 3H), 1.89-1.83 (m, 4H), 1.81-1.75 (m, 4H), 1.72 (s, 3H), 1.63-1.54 (m, 4H), 1.14 (d, *J* = 6.2 Hz, 7H), 1.08 (d, *J* = 6.8 Hz, 5H), 1.04-0.95 (m, 15H).

Synthesis of BacPROTAC-3a

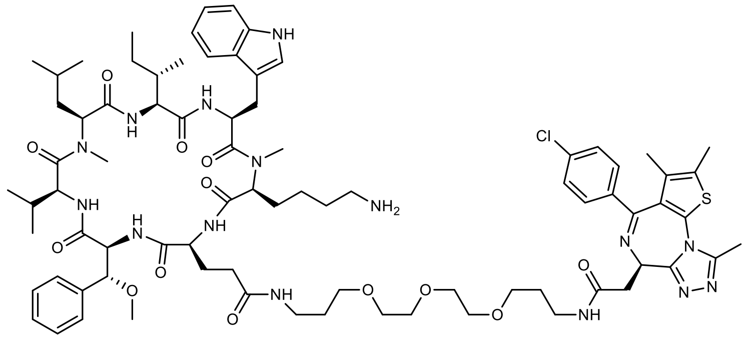

BacPROTAC-3a was synthesized analogously to the previously described solid phase peptide synthesis approach. Prior to SPPS, one further non-commercial amino acid building blocks was synthesized.

** Chemical synthesis of Fmoc-L-Glu(TOTA-(R)-JQ1)-OH*

1) Chemical synthesis of Fmoc-L-Glu(TOTA-(*R*)-JQ1)-O*t*Bu

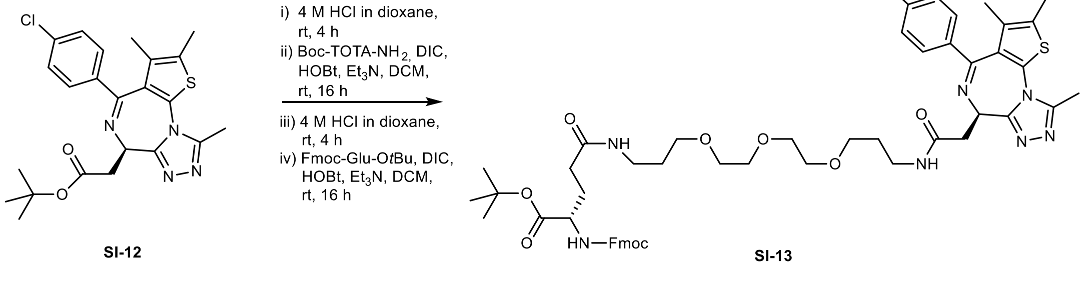

(-)-JQ1 (**SI-12**, 250 mg, 0.55 mmol) was dissolved in 4 M HCl in 1,4-dioxane (10 mL). The resulting solution was stirred for 16 hours at room temperature. The solvent was evaporated under reduced pressure and the resulting residue was used in the next step without further purification.

The obtained residue was dissolved in DCM (10 mL). Et_3N (174 µL, 2.75 mmol), HOBt monohydrate (148 mg, 1.1 mmol), DIC (78 µL, 1.1 mmol) and Boc-TOTA-NH_2 (122 mg, 0.82 mmol) were added. The resulting mixture was stirred at room temperature for 16 hours. The reaction mixture was diluted by addition saturated aq. $NaHCO_3$ (15 mL). The resulting phases were separated and the organic phase was washed with brine, followed by drying over $MgSO_4$. The solvent was removed under reduced pressure, yielding a residue that was used in the next step without further purification.

This residue was suspended in 4 M HCl in 1,4-dioxane (10 mL) and stirred for 1 hour. The solvent was removed by evaporation to dryness and the resulting residue was dissolved in DCM (10 mL), followed by addition of Et_3N (2.75 µL, 2.75 mmol), HOBt monohydrate (148 mg, 1.1 mmol), DIC (78 µL, 1.1 mmol) and Fmoc-L-Glu-O*t*Bu (350 mg, 0.82 mmol). The resulting mixture was stirred at room temperature for 16 hours. The organic phase was washed with brine, dried over $MgSO_4$ and evaporated under reduced pressure to dryness. The resulting residue was purified by silica gel column chromatography using 5-10% MeOH/DCM as the eluent system. Product containing fractions were pooled and evaporated to dryness, yielding 273 mg (0.27 mmol, 49%) of the desired product **SI-13**.

TLC (DCM/MeOH = 5:1): R_f = 0.7.

LC-MS (ESI): t_R = 10.88 min, *m/z* 1009.4 calcd. for $C_{53}H_{64}ClN_7O_9S$, found: 1010.2 $[M+H]^+$.

^{1}H NMR (400 MHz, DMSO-d6): δ = 8.19 (t, *J* = 5.3 Hz, 1H), 7.90 (d, *J* = 7.6 Hz, 2H), 7.79 (t, *J* = 5.5 Hz, 1H), 7.73 (d, *J* = 7.0 Hz, 2H), 7.67 (d, *J* = 8.1 Hz, 1H), 7.49 (d, *J* = 8.6 Hz, 2H), 7.44-7.39 (m, 4H), 7.33 (t, *J* = 7.3 Hz, 2H), 4.51 (t, *J* = 7.0 Hz, 1H), 4.33-4.19 (m, 3H), 3.97-3.84 (m, 1H), 3.54-3.41 (m, 9H), 3.38 (t, *J* = 6.4 Hz, 2H), 3.28-3.12 (m, 5H), 3.07 (q, *J* = 6.4 Hz, 2H), 2.59 (s, 2H), 2.40 (s, 2H), 2.16 (t, *J* = 7.6 Hz, 2H), 1.98-1.89 (m, 1H), 1.81-1.73 (m, 1H), 1.68 (t, *J* = 6.5 Hz, 2H), 1.64-1.58 (m, 4H), 1.39 (s, 9H).

2) Chemical synthesis of Fmoc-L-Glu(TOTA-(*R*)-JQ1)-OH

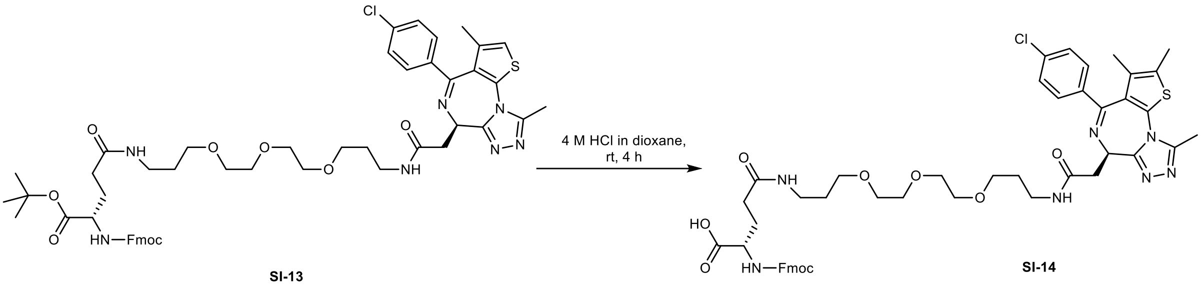

SI-13 **SI-14**

SI-13 (260 mg, 0.27 mmol) was dissolved in 4 M HCl in 1,4-dioxane (10 mL) and the resulting mixture was stirred for 2 hours at room temperature. The solution was evaporated under reduced pressure to dryness to yield 260 mg (0.41 mmol, >98%) of **SI-14**.

TLC (DCM/MeOH = 3:1): R_f = 0.4.

LC-MS (ESI): t_R = 9.21 min, *m/z* 953.4 calcd. for $C_{49}H_{56}ClN_7O_9S$, found: 954.3 $[M+H]^+$.

^1H NMR (400 MHz, DMSO-d6): δ = 8.30 (t, *J* = 5.3 Hz, 1H), 7.90 (d, *J* = 7.0 Hz, 2H), 7.86-7.79 (m, 1H), 7.75-7.70 (m, 2H), 7.68-7.60 (m, 1H), 7.53 (d, *J* = 8.2 Hz, 2H), 7.43 (q, *J* = 7.7 Hz, 4H), 7.34 (t, *J* = 7.0 Hz, 2H), 4.65 (t, *J* = 7.1 Hz, 1H), 4.32-4.17 (m, 3H), 4.06-3.90 (m, 1H), 3.74-3.59 (m, 3H), 3.53-3.41 (m, 9H), 3.38 (t, *J* = 6.2 Hz, 2H), 3.32-3.21 (m, 2H), 3.19-3.12 (m, 3H), 3.10-3.02 (m, 2H), 2.71 (s, 2H), 2.42 (s, 2H), 2.26-2.11 (m, 2H), 2.03-1.93 (m, 1H), 1.85-1.75 (m, 1H), 1.68 (t, *J* = 6.5 Hz, 2H), 1.63-1.57 (m, 4H).

* *Solid phase peptide synthesis of BacPROTAC-3a*

1) Loading of the resin

SI-5 (53 mg, 0.13 mmol) and DIPEA (43 µL, 0.25 mmol) were dissolved in DMF (5 mL) and added to 2-chlorotrityl resin (100 mg, 1.6 mmol g^{-1} initial loading). The resulting suspension was shaken for 16 h at room temperature. After removal of the solution, the resin was washed 2× with DMF, 2× with DCM and 2× with DMF. For capping, a mixture of DCM:MeOH:DIPEA (3:1:0.1, 4 mL) was added to the resin and the resulting suspension was shaken for 30 minutes at room temperature. The solvent was removed and the remained resin was washed again 2× with DMF, 2× with DCM and 2× with DMF.

2) Solid phase amino acid assembly and resin cleavage

The solid phase peptide synthesis and resin cleavage was performed as described for BacPROTAC-3, yielding 89 mg (0.050 mmol, 38% based on initial resin loading) that was used in the next step without further purification.

LC-MS (ESI): t_R = 9.57 min, *m/z* 1775.9 calcd. for $C_{90}H_{130}ClN_{15}O_{18}S$, found: 1778.1 $[M+H]^+$.

3) In solution cyclization

This linear peptide (89 mg, 0.050 mmol) was dissolved in DCM (67 mL, final concentration: 0.75 mM). Triethylamine (21 µL, 0.151 mmol), HOAt (20 mg, 0.147 mmol) and DIC (23 µL, 0.149 mmol) were added. The resulting solution was stirred at room temperature for 16 h. Aq. 5% $NaHCO_3$ (75 mL) was added and the organic layer was separated, dried over $MgSO_4$ and removed under reduced pressure to dryness to yield 61 mg (0.035 mmol, 70%) of a cyclized peptide that was used in the next step without further purification.

LC-MS (ESI): t_R = 12.88 min, *m/z* 1757.9 calcd. for $C_{90}H_{128}ClN_{15}O_{17}S$, found: 1659.1 $[M+H-Boc]^+$.

4) Final deprotection and purification

This cyclic peptide (61 mg, 0.035 mmol) was dissolved in DCM/TFA (1:3, 10 mL) and the resulting mixture was stirred for 16 hours at room temperature. The solvent was removed under reduced pressure and the residue was re-dissolved in DCM (25 mL). The organic layer was washed with saturated aq. $NaHCO_3$ and brine and dried over $MgSO_4$. After removal of the solvent by evaporation under reduced pressure, the residue was dried in high vacuum and purified by RP-HPLC with 0.1% TFA in water and 0.1% TFA in acetonitrile as eluent systems. Product containing fractions were pooled and lyophilized, thereby yielding 3.3 mg (0.002 mmol, 6%) of BacPROTAC-3a as a white powder.

LC-MS (ESI): t_R = 8.34 min, *m/z* 1557.8 calcd. for $C_{80}H_{112}ClN_{15}O_{13}S$, found: 1558.3 $[M+H]^+$.

HRMS (ESI): *m/z* 1558.8047 calcd. for $C_{80}H_{113}ClN_{15}O_{13}S^+$, found: 1558.7992 $[M+H]^+$.

^{1}H NMR (400 MHz, MeOD): δ = 7.96-7.78 (m, 1H), 7.60 (t, *J* = 7.7 Hz, 2H), 7.51-7.25 (m, 16H), 7.22-7.15 (m, 2H), 7.12-7.03 (m, 2H), 5.39 (d, *J* = 11.0 Hz, 1H), 5.24-5.18 (m, 1H), 5.13 (d, *J* = 3.6 Hz, 1H), 4.71-4.66 (m, 1H), 4.60 (d, *J* = 10.1 Hz, 1H), 4.54-

4.42 (m, 2H), 4.31 (t, J = 7.5 Hz, 1H), 3.71-3.65 (m, 6H), 3.64-3.58 (m, 8H), 3.57-3.48 (m, 4H), 3.39 (s, 3H), 3.19 (s, 3H), 2.90 (s, 2H), 2.76-2.69 (m, 6H), 2.57 (s, 3H), 2.48 (s, 4H), 1.90-1.76 (m, 8H), 1.73 (s, 3H), 1.67-1.54 (m, 4H), 1.11-0.87 (m, 26H).

Synthesis of sCym-1

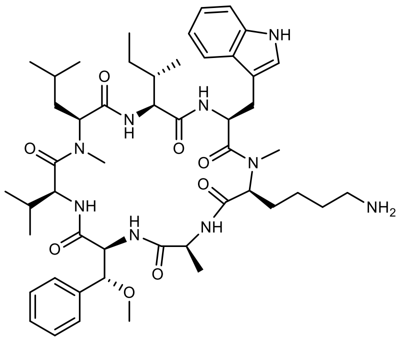

sCym-1 was synthesized *via* the previously described solid phase peptide synthesis approach used for BacPROTAC-2 and BacPROTAC-3. Therefore, only reagent quantities and product characterizations are provided.

Resin loading: Fmoc-L-Phe(3*R*-MeO)-OH (**SI-5**, 209 mg, 0.5 mmol) and DIPEA (170 µL, 1 mmol) were dissolved in DMF (5 mL). This solution was added to 2-chlorotrityl resin (150 mg, 1.6 mmol g^{-1} initial loading). The resulting suspension was shaken at room temperature for 4 hours. Washing of the resin and the capping step were performed as described for BacPROTAC-2. The subsequent solid phase peptide synthesis was performed as described before.

Cleavage from the resin: A mixture of DCM:hexafluoroisopropanol (3:1, 5 mL) was added to the resin. The resulting suspension was shaken at room temperature for 20 min. The cleavage solution was removed from the resin and transferred into a flask. The resin was washed 3× with DCM (2 mL) and the washing solutions were combined with the cleavage solution. The resulting solution was evaporated under reduced pressure to dryness to yield 156 mg (0.138 mmol, 58 %) of the desired product.

In solution cyclization: This peptide (156 mg, 0.138 mmol) was dissolved in DCM (184 mL, final concentration: 0.75 mM). DIPEA (184 µL, 1.10 mmol) and 50% propanephosphonic acid anhydride in ethyl acetate (T3P, 351 µL, 0.55 mmol) were added. The resulting solution was stirred at room temperature for one hour. Brine (100 mL) was added, the organic phase was separated, dried over $MgSO_4$ and the organic

phase was removed under reduced pressure to obtain 135 mg (0.121 mmol, 88 %) of the cyclized peptide which was used in the next step without further purification.

Protecting group cleavage and purification: This cyclized peptide (52 mg, 0.046 mmol) was dissolved in a TFA:DCM (3:1, 5 mL) mixture and the resulting solution was stirred at room temperature for 16 h. The solvent was removed by evaporation and the resulting residue was purified by RP-HPLC with 0.1% TFA in water and 0.1% TFA in acetonitrile as eluent systems. Product containing fractions were pooled and lyophilized, thereby yielding 7 mg (0.008 mmol, 17%) of sCym-1 as a white powder.

LC-MS (ESI): t_R = 7.73 min, *m/z* 915.6 calcd. for $C_{49}H_{73}N_9O_8$, found: 916.4 $[M+H]^+$.

HRMS (ESI): *m/z* 916.5660 calcd. for $C_{49}H_{74}N_9O_8^+$, found: 916.5669 $[M+H]^+$.

^{1}H NMR (400 MHz, DMSO-d6): δ = 10.92 (s, 1H), 9.45 (d, *J* = 3.0 Hz, 1H), 9.15 (d, *J* = 7.8 Hz, 1H), 8.61 (d, *J* = 10.3 Hz, 1H), 8.10 (d, *J* = 9.5 Hz, 1H), 7.56 (s, 3H), 7.47 (q, *J* = 7.2 Hz, 2H), 7.38 (d, *J* = 8.6 Hz, 1H), 7.29 (t, *J* = 7.8 Hz, 2H), 7.24-7.18 (m, 4H), 7.09 (t, *J* = 7.3 Hz, 1H), 6.99 (t, *J* = 6.9 Hz, 1H), 5.16 (d, *J* = 12.1 Hz, 1H), 4.84-4.80 (m, 1H), 4.48-4.37 (m, 2H), 4.25 (d, *J* = 9.2 Hz, 2H), 4.17-4.12 (m, 3H), 3.20-3.06 (m, 3H), 3.00 (s, 3H), 2.71 (s, 3H), 2.59-2.53 (m, 1H), 2.36 (s, 3H), 2.31-2.22 (m, 2H), 1.88-1.80 (m, 1H), 1.55-1.36 (m, 3H), 1.18-1.07 (m, 3H), 1.04 (d, *J* = 6.4 Hz, 3H), 0.96-0.82 (m, 17H), 0.77 (d, *J* = 7.2 Hz, 3H).

Chemical Synthesis of BacPROTAC-4, -4a, -5, -5a

Reactions were monitored by LC/MS (*Shimadzu Prominence-i LC-2030*, column: *Phenomenex Onyx C18*, 50 x 4.6 mm, *Shimadzu LCMS-2020*, (ESI)). Flash chromatography (reversed phase) was conducted with a *Büchi Reveleris PREP* on *Büchi Flashpure Select C18* cartridges, H_2O/MeCN gradient). The compounds were dried by lyophilization from MeCN/H_2O over night. ^{1}H and ^{13}C spectra were recorded with Bruker AV 500 or Bruker Avance Neo500 [500 MHz (^{1}H), 126 MHz (^{13}C)] spectrometers in $CDCl_3$ at 298 K.Chemical shifts are reported in ppm relative to $Si(CH_3)_4$. The signals of residual $CHCl_3$ in $CDCl_3$ (δ(^{1}H, $CHCl_3$) = 7.26 ppm, δ(^{13}C, $CDCl_3$) = 77.16 ppm) were used as the internal standard. Multiplicities are reported as (singlet), d (doublet), t (triplet), q (quartet) and m (multiplet). High resolution mass spectra were recorded on a *Bruker MAXIS 4G* UHR-TOF (ESI). Optical rotations were measured on a *Jasco P-2000* polarimeter in a thermostated (20 °C ± 1 °C) cuvette

(path length: 50 mm, λ = 589 nm). The concentrations are given in g/100 ml. HPLC analyses were performed with a Shimadzu HPLC system and a Phenomenex Luna column (3 µM C18(2), 50 x 4.6 mm) at 40 °C using a gradient of 10 mM phosphate buffer (pH = 2.6) and acetonitrile (90% − 0% (12 min), 0% (6 min), 1 mL min^{-1}).

Chemical Synthesis of BacPROTAC-4

Synthesis of (S)-N-(14-Azido-3,6,9,12-tetraoxatetradecyl)-2-(4-(4-chlorophenyl)-2,3,9-trimethyl-6H-thieno[3,2-f][1,2,4]triazolo[4,3-a][1,4]diazepin-6-yl)acetamide (**SI-15**)

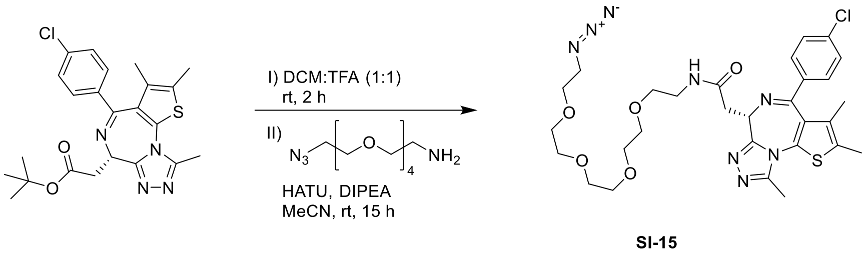

(+)-JQ1 (**SI-9**, 150 mg, 330 µmol) was dissolved in DCM (160 µL) and TFA (160 µL), was added. After stirring for 1 h at rt, the solvent was evaporated in vacuo. The resiude was dissolved in $CHCl_3$ and evaporated in vacuo (3 times). The crude product was then dissolved in MeCN (3.3 mL), 14-azido-3,6,9,12-tetraoxatetradecan-1-amine (129 mg, 490 µmol), HATU (137 mg, 360 µmol) and DIPEA (85.0 µL) were added successively. After 15 h, the solvent was removed *in vacuo*, the residue was redissolved in EtOAc, washed with 1 M $KHSO_4$, 1 M LiCl, sat. $NaHCO_3$ and sat. NaCl solutions, dried (Na_2SO_4) and evaporated. Flash chromatography (H_2O/MeCN 90:10 - 5:95) followed by lyophilizaiton afforded **SI-15** (88.2 mg, 137 µmol, 42%) as an off-white, amorphous solid.

$[\alpha]_{20}^{D}$ = +32.9 (c 1.0, $CHCl_3$).

HRMS (ESI): calcd for $C_{29}H_{38}ClN_8O_5S^+$ $(M+H)^+$: 645.2374; found: 645.2371.

^1H NMR (500 MHz, $CDCl_3$) δ 1.65 (s, 3H), 2.38 (s, 3H), 2.65 (s, 3H), 3.33 – 3.41 (m, 3H), 3.46 – 3.54 (m, 3H), 3.55 – 3.63 (m, 2H), 3.63 – 3.71 (m, 14H), 4.64 (t, *J* = 7.2 Hz, 1H), 6.89 – 6.99 (m, 1H), 7.29 – 7.34 (m, 2H), 7.39 (d, *J* = 8.0 Hz, 2H). *^{13}C NMR* (126 MHz, $CDCl_3$) δ 11.9, 13.2, 14.5, 39.2, 39.5, 50.8, 54.4, 70.1, 70.5, 70.7, 70.7,

70.7, 70.8, 70.8, 128.8, 130.0, 130.6, 130.8, 131.0, 132.2, 136.8, 136.8, 149.9, 155.8, 163.9, 170.6.

Synthesis of BacPROTAC-4

Cyclic peptide **SI-16** was prepared by solution phase synthesis in analogy to a previously published procedure using Fmoc-L-propargylglycine as the 7th amino acid (Kiefer et al., 2019).

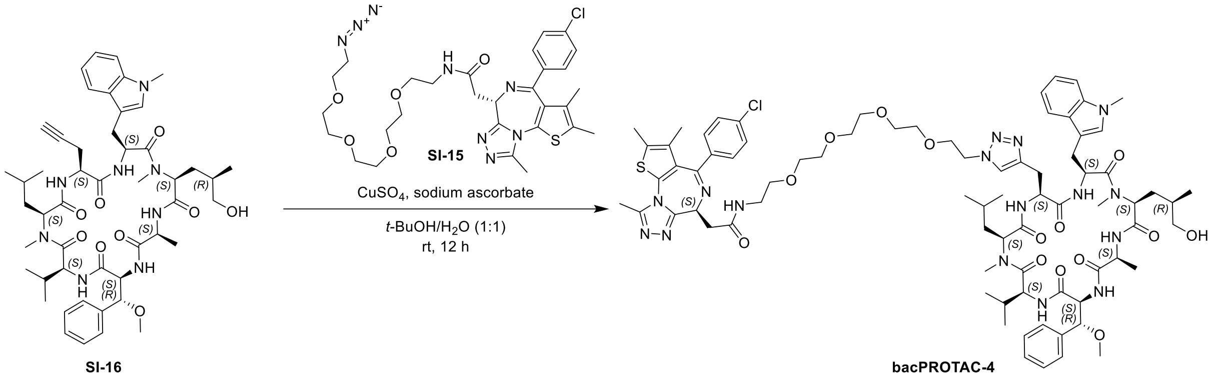

In a 1.5 ml vial, **SI-16** (18.0 mg, 20.0 µmol) and **SI-15** (15.3 mg, 24.0 µmol) were dissolved in *t*-BuOH (190 µL)/H_2O (190 µL). 1 M $CuSO_4$ (9.86 µL, 9.86 µmol) and 1 M sodium ascorbate (12.0 µL, 12.0 µmol) were added, the vial was flushed with Argon and stirred at rt for 12 h. Then, the reaction mixture was concentrated in vacuo and the residue was purified by flash chromatography (H_2O/MeCN 70:30 – 5:95). After lyophilization, **BacPROTAC-4** (27.0 mg, 17.0 µmol, 88%) was obtained as a white amorphous solid.

$[\alpha]_{20}^{D}$ = –39.8 (c 0.5, $CHCl_3$).

HRMS (ESI): calcd for $C_{78}H_{105}ClN_{16}O_{14}S^+$ $(M+H)^+$: 1557.7478; found: 1557.7449.

^1H NMR (500 MHz, $CDCl_3$) δ -0.39 – -0.23 (m, 1H), 0.24 (d, *J* = 6.8 Hz, 3H), 0.83 – 0.99 (m, 11H), 1.05 (d, *J* = 6.7 Hz, 3H), 1.43 (d, *J* = 7.4 Hz, 3H), 1.47 – 1.54 (m, 2H), 1.65 (s, 3H), 1.85 – 1.94 (m, 1H), 1.96 – 2.04 (m, 1H), 2.13 – 2.29 (m, 3H), 2.38 (s, 3H), 2.54 – 2.74 (m, 8H), 2.86 (s, 3H), 2.89 – 3.02 (m, 2H), 3.04 – 3.09 (m, 1H), 3.18 – 3.34 (m, 2H), 3.35 (s, 3H), 3.61 – 3.72 (m, 17H), 3.86 – 4.13 (m, 2H), 4.45 – 4.55 (m, 1H), 4.59 (t, *J* = 5.3 Hz, 2H), 4.63 – 4.73 (m, 3H), 4.75 – 4.80 (m, 2H), 4.82 – 4.87 (m, 1H), 4.94 (t, *J* = 4.9 Hz, 1H), 5.13 (d, *J* = 5.2 Hz, 1H), 6.77 (s, 1H), 6.96 – 7.11 (m, 7H), 7.17 (t, *J* = 7.6 Hz, 1H), 7.22 (d, *J* = 8.2 Hz, 1H), 7.29 – 7.35 (m, 2H), 7.39 – 7.44 (m, 4H), 7.45 – 7.51 (m, 2H), 7.93 (d, *J* = 6.1 Hz, 1H), 8.16 (d, *J* = 8.9 Hz, 1H), 8.26

(d, J = 9.2 Hz, 1H), 8.61 (d, J = 9.8 Hz, 1H). *^{13}C NMR* (126 MHz, $CDCl_3$) δ 11.8, 13.2, 14.5, 17.5, 19.3, 19.9, 20.5, 22.8, 23.6, 25.1, 27.5, 29.0, 29.8, 30.9, 32.7, 32.8, 33.2, 38.8, 39.0, 39.5, 50.1, 50.2, 51.2, 52.1, 54.3, 54.7, 56.4, 57.9, 66.1, 69.4, 70.0, 70.4, 70.5, 70.6, 70.6, 70.7, 70.7, 80.0, 108.7, 109.5, 118.8, 119.4, 122.1, 123.4, 127.7, 127.7, 128.1, 128.3, 128.7, 128.8, 130.1, 130.7, 130.9, 131.0, 132.1, 135.4, 136.8, 136.8, 136.9, 141.6, 155.8, 163.9, 167.4, 169.7, 170.6, 170.7, 170.8, 170.9, 171.2, 172.6.

Chemical Synthesis of bacPROTAC-4a

SI-17 was prepared by solution phase peptide synthesis in analogy to a previously published procedure using the enantiomeric starting materials and Fmoc-D-propargylglycine as the 7th amino acid (Kiefer et al., 2019).

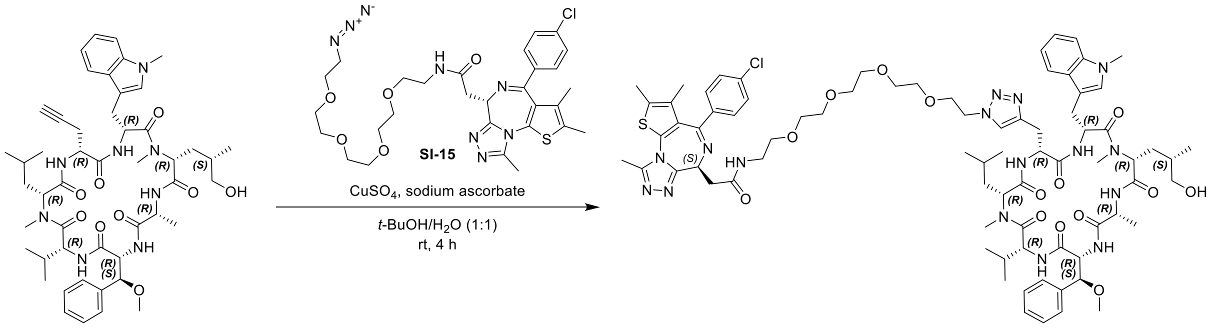

SI-17 **bacPROTAC-4 distomer**

In a 1.5 mL vial, **SI-17** (5.5 mg, 6.02 µmol) and **SI-15** (5.05 mg, 7.83 µmol) were dissolved in t-BuOH (60 µL)/H_2O (60 µL). 1 M $CuSO_4$ (4.82 µL, 4.82 µmol) and 1 M sodium ascorbate (3.01 µL, 3.01 µmol) were added, the vial was flushed with Argon and stirred at rt for 4 h. The reaction mixture was concentrated in vacuo and the residue was purified by RP flash chromatography (H_2O/MeCN 90:10 – 5:95). After lyophilization, **BacPROTAC-4a** (9.5 mg, 90% purity (HPLC), 5.49 µmol, 91% yield) was obtained as a white amorphous solid.

$[\alpha]^{D}_{20}$ = +54.5 (c 0.2, $CHCl_3$).

HRMS (ESI): calcd for $C_{78}H_{105}ClN_{16}O_{14}S^+$ $[M+H]^+$: 1557.7478; found: 1557.7402

^1H NMR (500 MHz, $CDCl_3$): δ -0.38 – -0.24 (m, 1H), 0.24 (d, J = 6.7 Hz, 3H), 0.84 – 0.86 (m, 1H), 0.92 – 0.99 (m, 9H), 1.06 (d, J = 6.6 Hz, 3H), 1.13 – 1.23 (m, 2H), 1.42 (d, J = 7.4 Hz, 3H), 1.49 – 1.59 (m, 2H), 1.66 (s, 3H), 1.84 – 1.95 (m, 1H), 2.05 – 2.11 (m, 1H), 2.22 – 2.29 (m, 2H), 2.38 (s, 3H), 2.61 (d, J = 11.2 Hz, 6H), 2.91 – 2.95 (m,

2H), 2.95 – 2.99 (m, 1H), 3.06 (dd, *J* = 11.2, 4.0 Hz, 1H), 3.15 – 3.23 (m, 1H), 3.36 (s, 3H), 3.41 – 3.53 (m, 4H), 3.58 – 3.77 (m, 20H), 3.93 – 3.99 (m, 1H), 4.05 – 4.12 (m, 1H), 4.43 – 4.62 (m, 3H), 4.63 – 4.77 (m, 4H), 4.80 – 4.88 (m, 1H), 4.95 (t, *J* = 4.9 Hz, 1H), 5.14 (d, *J* = 5.3 Hz, 1H), 6.76 (s, 1H), 6.99 – 7.14 (m, 6H), 7.14 – 7.19 (m, 1H), 7.22 (d, *J* = 8.3 Hz, 1H), 7.28 – 7.33 (m, 3H), 7.36 – 7.43 (m, 3H), 7.45 – 7.56 (m, 2H), 8.15 (d, *J* = 8.9 Hz, 1H), 8.27 (d, *J* = 8.6 Hz, 1H), 8.57 (d, *J* = 9.8 Hz, 1H). *^{13}C NMR* (126 MHz, CDCl3) δ 11.8, 13.2, 14.6, 17.4, 19.4, 19.9, 20.7, 22.9, 23.7, 25.2, 27.7, 29.1, 29.8, 29.8, 31.0, 32.8, 33.3, 38.7, 39.0, 39.6, 50.2, 51.2, 52.4, 54.3, 54.7, 56.4, 58.0, 58.9, 59.5, 66.2, 69.3, 70.0, 70.4, 70.5, 70.6, 70.6, 70.7, 70.7, 80.0, 108.6, 109.6, 118.8, 119.0, 119.4, 122.2, 123.3, 127.7, 127.8, 128.1, 128.4, 128.4, 128.5, 128.8, 128.8, 128.9, 130.1, 131.2, 135.4, 136.7, 136.9, 150.1, 155.8, 163.9, 167.6, 169.7, 170.5, 170.7, 170.9, 171.2, 172.5.

Chemical Synthesis of BacPROTAC-5

(S)-N-(5-azidopentyl)-2-(4-(4-chlorophenyl)-2,3,9-trimethyl-6H-thieno[3,2-f][1,2,4]triazolo[4,3-a][1,4]diazepin-6-yl)acetamide (**SI-18**)

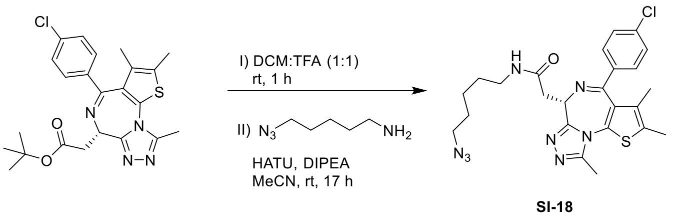

(+)-JQ1 (**SI-9**, 100 mg, 219 µmol) was dissolved in DCM (160 µL) and TFA (160 µL), was added. After stirring for 1 h at rt, the solvent was evaporated in vacuo. The residue was dissolved in $CHCl_3$ and evaporated in vacuo (3 times). The crude product was then dissolved in MeCN (2.2 mL), 5-azidopentan-1-amine (64.7 mg, 472 µmol), HATU (100 mg, 263 µmol) and DIPEA (119 µL, 920 µmol) were added successively. After 17 h, the solvent was removed *in vacuo*, the residue was redissolved in EtOAc, washed with 1 M $KHSO_4$, sat. $NaHCO_3$ and sat. NaCl solutions, dried (Na_2SO_4) and evaporated. Flash chromatography (H_2O/MeCN 90:10 - 5:95) followed by lyophilizaiton afforded **SI-18** (101 mg, 198 µmol, 90%) as an off-white, amorphous solid.

$[\alpha]_{20}^{D}$= +26.2 (c 0.5, $CHCl_3$).

HRMS (ESI) calcd for $C_{24}H_{27}ClN_8OS$: 511.1790; found: 511.1796.

^{1}H NMR (500 MHz, $CDCl_3$) δ 1.35 – 1.43 (m, 2H), 1.51 – 1.63 (m, 4H), 1.67 (s, 3H), 2.40 (s, 3H), 2.67 (s, 3H), 3.23 (t, *J* = 6.9 Hz, 2H), 3.25 – 3.37 (m, 3H), 3.57 (dd, *J* = 14.2, 8.0 Hz, 1H), 4.62 (dd, *J* = 8.0, 6.0 Hz, 1H), 6.71 (t, *J* = 5.8 Hz, 1H), 7.32 (d, *J* = 8.7 Hz, 2H), 7.40 (d, *J* = 8.5 Hz, 2H). *^{13}C NMR* (126 MHz, $CDCl_3$) δ 11.9, 13.2, 14.5, 24.1, 28.6, 29.2, 39.5, 39.6, 51.4, 54.6, 128.9, 130.0, 130.6, 131.1, 131.1, 132.2, 136.6, 137.0, 150.0, 155.7, 164.1, 170.5.

Synthesis of BacPROTAC-5

Cyclic peptide **SI-19** was prepared by solution phase synthesis in analogy to a previously published procedure using N$^{\alpha}$-Alloc-N^{1}-propargyl-L-tryptophan as the 6th amino acid (Kiefer et al., 2019).

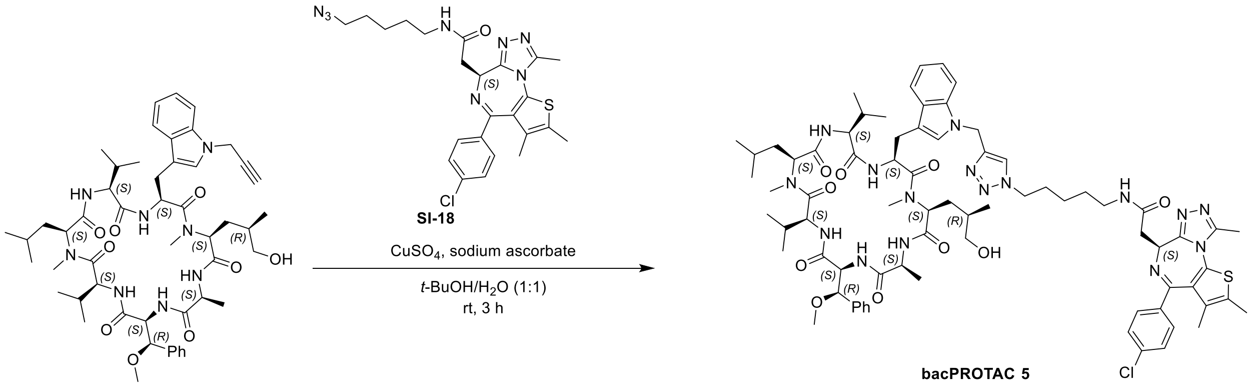

In a 1.5 mL vial, **SI-19** (15.9 mg, 16.9 µmol) and **SI-18** (11.4 mg, 22.3 µmol) were dissolved in t-BuOH (169 µL)/H_2O (169 µL). 1 M $CuSO_4$ (8.45 µL, 8.45 µmol) and 1 M sodium ascorbate (13.5 µL, 13.5 µmol) were added, the vial was flushed with Argon and stirred at rt for 3 h. The reaction mixture was concentrated in vacuo and the residue was purified by RP flash chromatography (H_2O/MeCN 90:10 – 5:95). After lyophilization, **BacPROTAC-5** (23.8 mg, 95% purity (HPLC), 15.6 µmol, 92% yield) was obtained as a white amorphous solid.

$[\alpha]_{20}^{D}$ = –92.6 (c 0.5, $CHCl_3$).

HRMS calcd for $C_{75}H_{99}ClN_{16}O_{10}S^+$ $[M+H]^+$: 1451.7212; found: 1451.7168.

¹H NMR (500 MHz, $CDCl_3$) δ -0.75 – -0.66 (m, 1H), 0.11 (d, *J* = 6.7 Hz, 3H), 0.38 (d, *J* = 6.5 Hz, 3H), 0.52 (d, *J* = 6.6 Hz, 3H), 0.60 – 0.70 (m, 1H), 0.95 (d, *J* = 6.7 Hz, 10H), 1.03 – 1.09 (m, 4H), 1.15 (d, *J* = 7.1 Hz, 3H), 1.28 – 1.38 (m, 3H), 1.49 – 1.59 (m, 3H), 1.67 (s, 3H), 1.74 – 1.82 (m, 1H), 1.85 – 1.93 (m, 2H), 2.19 – 2.26 (m, 1H), 2.26 – 2.34 (m, 1H), 2.37 – 2.42 (m, 4H), 2.48 (s, 3H), 2.63 – 2.71 (m, 2H), 2.76 – 2.82 (m, 4H), 3.01 – 3.08 (m, 1H), 3.13 – 3.20 (m, 2H), 3.27 – 3.39 (m, 5H), 3.43 – 3.51 (m, 1H), 3.52 – 3.61 (m, 1H), 3.91 (t, *J* = 9.4 Hz, 1H), 4.26 – 4.42 (m, 3H), 4.47 (t, *J* = 8.7 Hz, 1H), 4.64 – 4.70 (m, 1H), 4.71 – 4.78 (m, 2H), 4.82 (dd, *J* = 10.6, 3.4 Hz, 1H), 4.89 (t, *J* = 5.1 Hz, 1H), 5.07 (d, *J* = 5.4 Hz, 1H), 5.20 – 5.31 (m, 1H), 5.31 – 5.43 (m, 1H), 6.84 (s, 1H), 7.09 (t, *J* = 7.5 Hz, 1H), 7.13 – 7.24 (m, 8H), 7.33 (d, *J* = 8.2 Hz, 2H), 7.37 – 7.43 (m, 3H), 7.48 (d, *J* = 7.9 Hz, 1H), 7.53 – 7.57 (m, 1H), 7.60 – 7.71 (m, 1H), 8.09 (d, *J* = 8.0 Hz, 1H), 8.21 (d, *J* = 9.3 Hz, 1H), 8.39 (d, *J* = 10.3 Hz, 1H). *¹³C NMR* (126 MHz, $CDCl_3$) δ 11.9, 13.3, 14.6, 16.5, 18.6, 19.5, 19.9, 20.1, 21.0, 22.7, 23.8, 24.1, 25.3, 28.1, 28.9, 29.2, 29.6, 29.9, 30.9, 31.9, 32.1, 32.6, 39.0, 39.2, 39.3, 41.3, 50.5, 51.1, 55.4, 56.1, 57.9, 58.8, 59.0, 59.0, 66.5, 70.7, 80.0, 108.8, 109.9, 118.8, 120.0, 122.6, 126.3, 127.8, 128.2, 128.2, 128.8, 128.8, 130.2, 131.2, 131.4, 135.1, 136.3, 137.2, 164.3, 168.2, 168.8, 169.9, 170.6, 170.7, 171.5, 171.7, 172.3.

Chemical Synthesis of BacPROTAC-5a

(R)-N-(5-azidopentyl)-2-(4-(4-chlorophenyl)-2,3,9-trimethyl-6H-thieno[3,2-f][1,2,4]triazolo[4,3-a][1,4]diazepin-6-yl)acetamide (**SI-20**)

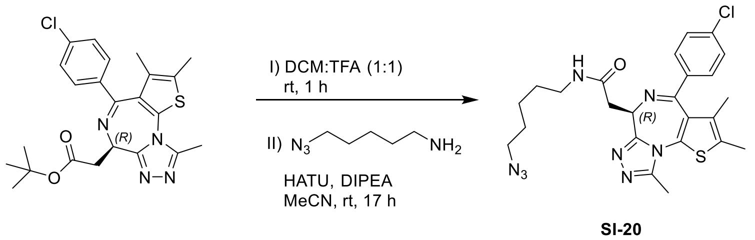

SI-12 **SI-20**

(-)-JQ1 (**SI-12**, 46.8 mg, 102 µmol) was dissolved in DCM (500 µL) and TFA (500 µL) was added. After stirring for 1 h at rt, the solvent was evaporated in vacuo. The residue was dissolved in $CHCl_3$ and evaporated in vacuo (3 times). The crude product was then dissolved in MeCN (1.1 mL), 5-azidopentan-1-amine (32.7 mg, 255 µmol), HATU (97.0 mg, 255 µmol) and DIPEA (71.0 µL, 408 µmol) were added successively. After 17 h, the solvent was removed *in vacuo*, the residue was redissolved in EtOAc,

washed with 1 M $KHSO_4$, sat. $NaHCO_3$ and sat. NaCl solutions, dried (Na_2SO_4) and evaporated. Flash chromatography (H_2O/MeCN 90:10 - 5:95) followed by lyophilizaiton afforded **SI-20** (47.0 mg, 92.0 µmol, 90%) as an off-white, amorphous solid.

$[\alpha]_{20}^{D} =$ –20.4 (c 0.5, $CHCl_3$).

HRMS (ESI) calcd for $C_{24}H_{27}ClN_8OS$: 511.1790; found: 511.1787.

^1H NMR (500 MHz, $CDCl_3$) δ 1.33 – 1.41 (m, 2H), 1.49 – 1.62 (m, 4H), 1.65 (s, 3H), 2.38 (s, 3H), 2.65 (s, 3H), 3.22 (t, *J* = 6.9 Hz, 2H), 3.24 – 3.39 (m, 3H), 3.56 (dd, *J* = 14.2, 7.9 Hz, 1H), 4.62 (dd, *J* = 7.9, 6.1 Hz, 1H), 6.87 (t, *J* = 5.8 Hz, 1H), 7.30 (d, *J* = 8.7 Hz, 2H), 7.38 (d, *J* = 8.1 Hz, 2H). *^{13}C NMR* (126 MHz, $CDCl_3$) δ 11.9, 13.2, 14.5, 24.1, 28.6, 29.2, 39.4, 39.5, 51.4, 54.6, 128.8, 129.9, 130.5, 131.0, 131.0, 132.2, 136.6, 136.9, 150.0, 155.8, 164.0, 170.5.

Synthesis of BacPROTAC-5a

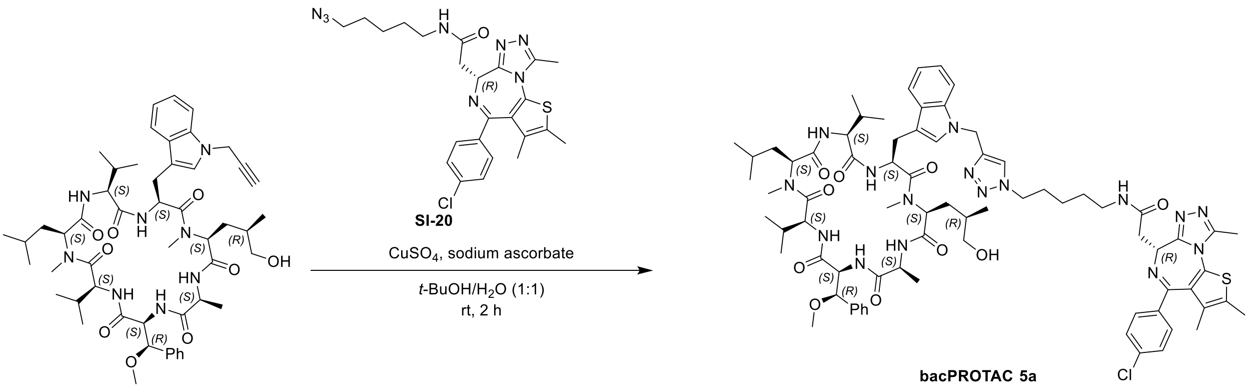

SI-19

In a 1.5 mL vial, **SI-19** (15.4 mg, 16.4 µmol) and **SI-20** (11.0 mg, 21.5 µmol) were dissolved in t-BuOH (164 µL)/H_2O (164 µL). 1 M $CuSO_4$ (8.18 µL, 8.18 µmol) and 1 M sodium ascorbate (13.1 µL, 13.1 µmol) were added, the vial was flushed with Argon and stirred at rt for 2 h. The reaction mixture was concentrated in vacuo and the residue was purified by RP flash chromatography (H_2O/MeCN 90:10 – 5:95). After lyophilization, **BacPROTAC-5a** (20.8 mg, 97% purity (HPLC), 13.9 µmol, 85% yield) was obtained as a white amorphous solid.

$[\alpha]_{20}^{D}$= –84.3 (c 0.5, $CHCl_3$).

HRMS calcd for $C_{75}H_{99}ClN_{16}O_{10}S^+$ $[M+H]^+$: 1451.7212; found: 1451.7102.

^{1}H NMR (500 MHz, $CDCl_3$) δ -0.75 (t, *J* = 9.3 Hz, 1H), 0.15 (d, *J* = 6.7 Hz, 3H), 0.58 (d, *J* = 6.5 Hz, 3H), 0.61 (d, *J* = 6.6 Hz, 3H), 0.74 (t, *J* = 7.2 Hz, 1H), 0.96 (dd, *J* = 6.6, 4.6 Hz, 10H), 1.08 (d, *J* = 6.7 Hz, 4H), 1.16 (d, *J* = 7.2 Hz, 3H), 1.29 – 1.38 (m, 2H), 1.48 – 1.61 (m, 4H), 1.68 (s, 3H), 1.76 – 1.83 (m, 1H), 1.85 – 1.95 (m, 2H), 2.22 – 2.28 (m, 1H), 2.29 – 2.36 (m, 1H), 2.41 (s, 3H), 2.47 (s, 3H), 2.64 – 2.69 (m, 2H), 2.84 (s, 3H), 3.05 (dd, *J* = 10.9, 4.1 Hz, 1H), 3.11 (dd, *J* = 13.5, 4.9 Hz, 1H), 3.15 – 3.21 (m, 1H), 3.25 – 3.35 (m, 2H), 3.35 – 3.43 (m, 5H), 3.56 – 3.62 (m, 1H), 4.08 (t, *J* = 9.4 Hz, 1H), 4.29 – 4.41 (m, 3H), 4.47 (t, *J* = 8.6 Hz, 1H), 4.67 – 4.78 (m, 3H), 4.86 (dd, *J* = 10.7, 3.4 Hz, 1H), 4.91 (t, *J* = 5.1 Hz, 1H), 5.09 (d, *J* = 5.4 Hz, 1H), 5.23 – 5.30 (m, 1H), 5.34 – 5.42 (m, 1H), 6.92 (s, 1H), 7.06 – 7.12 (m, 2H), 7.16 – 7.24 (m, 8H), 7.32 – 7.35 (m, 2H), 7.37 – 7.43 (m, 3H), 7.45 – 7.48 (m, 1H), 8.11 (d, *J* = 7.9 Hz, 1H), 8.23 (d, *J* = 9.2 Hz, 2H), 8.35 (d, *J* = 10.3 Hz, 1H). *^{13}C NMR* (126 MHz, $CDCl_3$) δ 11.9, 13.3, 14.6, 16.5, 18.8, 19.5, 20.1, 20.1, 21.0, 22.7, 23.8, 25.4, 28.2, 28.5, 29.1, 29.7, 29.8, 31.0, 31.9, 32.0, 32.6, 38.9, 39.1, 41.6, 50.5, 51.1, 55.4, 56.1, 57.9, 58.9, 59.0, 59.0, 66.6, 80.1, 108.7, 110.0, 118.7, 120.0, 122.7, 126.7, 127.8, 128.2, 128.3, 128.8, 128.9, 130.2, 131.3, 131.7, 135.2, 136.1, 136.3, 137.4, 164.5, 168.6, 168.8, 169.9, 170.4, 170.8, 171.4, 171.7, 172.3.

BacPROTAC-1

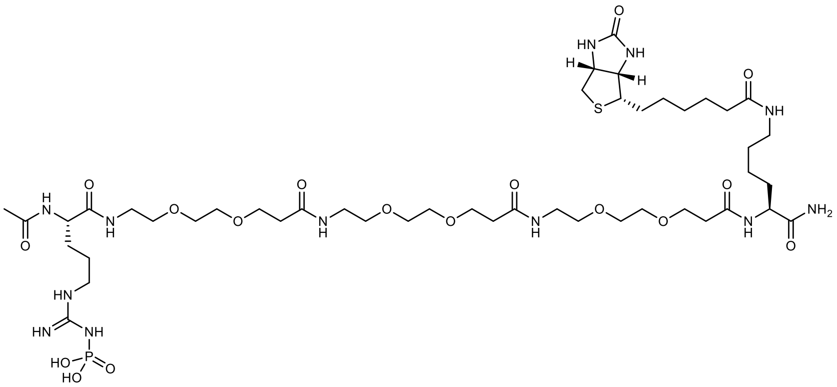
HN
NH
O
H
H
S
O
NH
O
H
N
O
O
N
H
O
O
O
N
H
O
O
O
N
H
O
O
O
N
H
NH2
O
HN
HN
NH
HO
P
O
HO

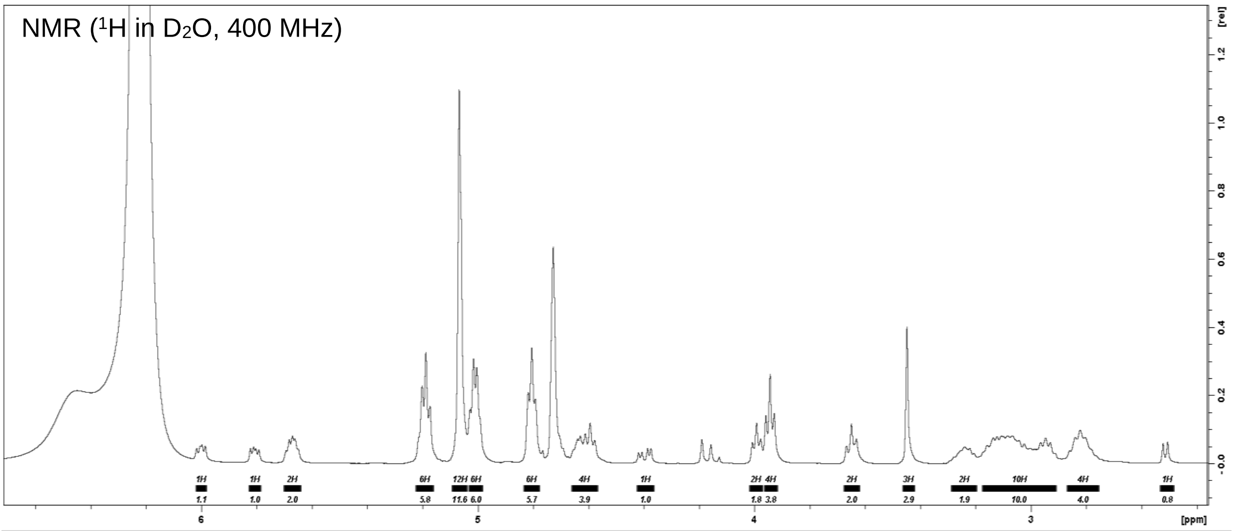
NMR (¹H in D₂O, 400 MHz)
[rel]
[ppm]

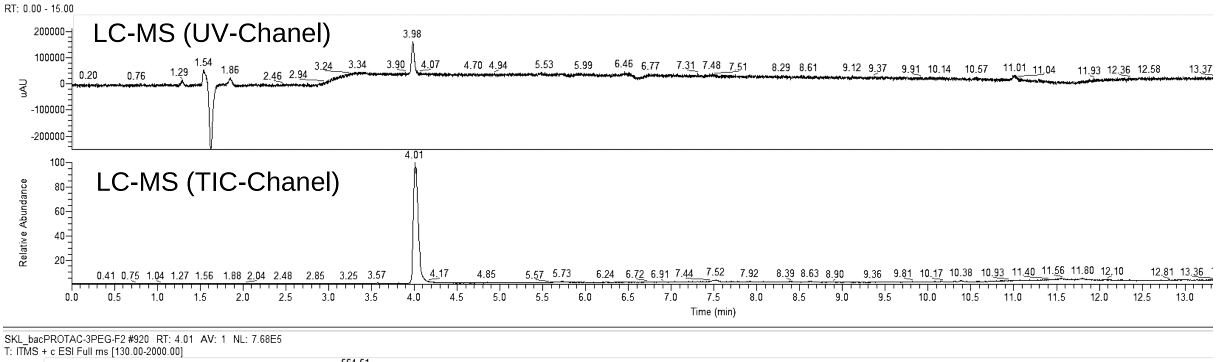
RT: 0.00 - 15.00
LC-MS (UV-Chanel)
uAU
LC-MS (TIC-Chanel)
Relative Abundance
Time (min)

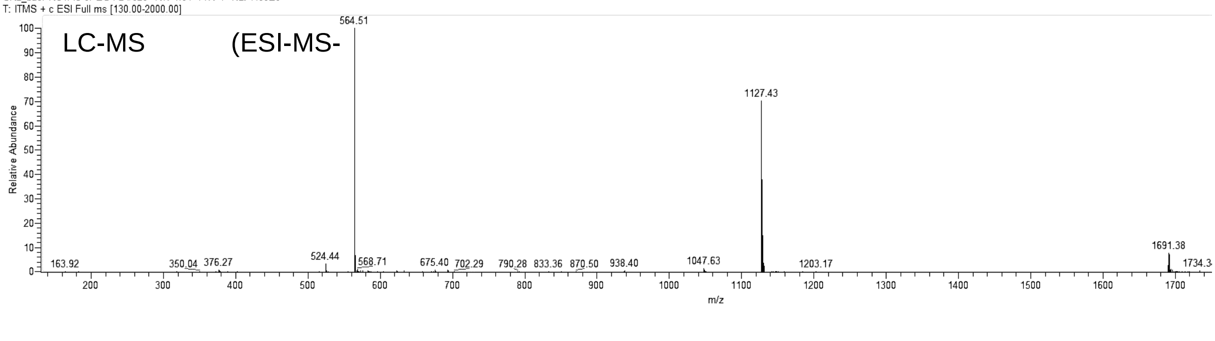
SKL_bacPROTAC-3PEG-F2 #920 RT: 4.01 AV: 1 NL: 7.68E5
T: ITMS + c ESI Full ms [130.00-2000.00]
LC-MS (ESI-MS-
Relative Abundance
564.51
1127.43
1691.38
m/z

BacPROTAC-1a

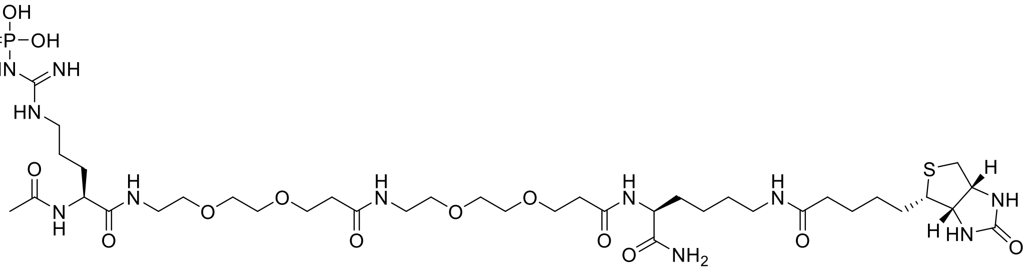

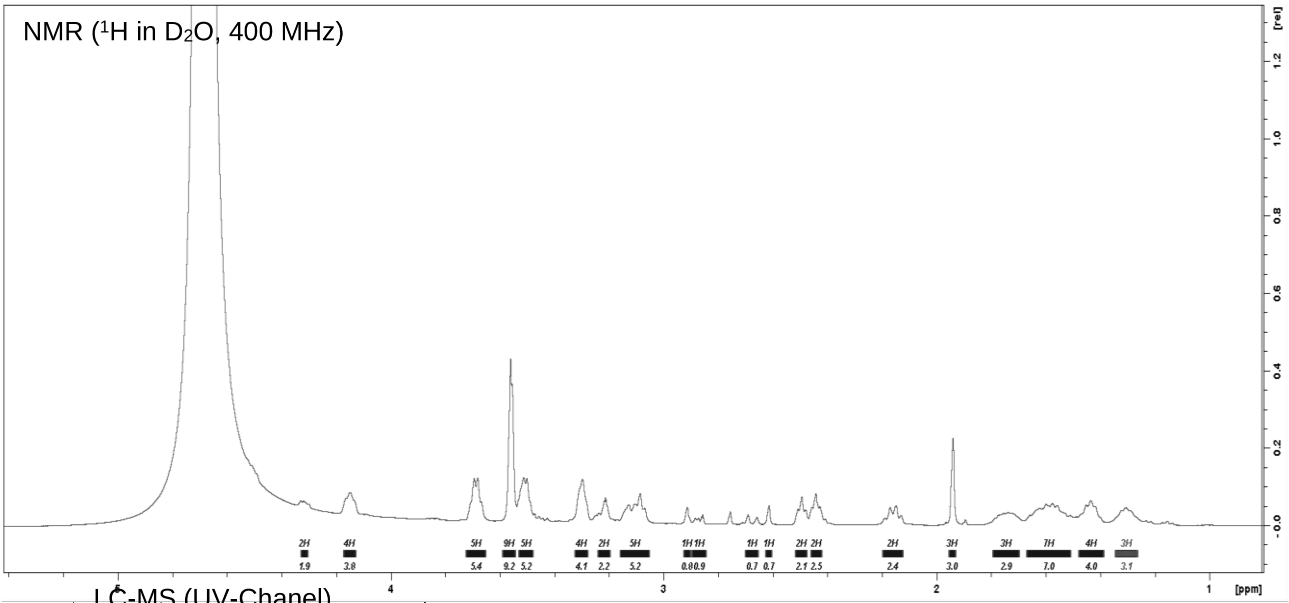

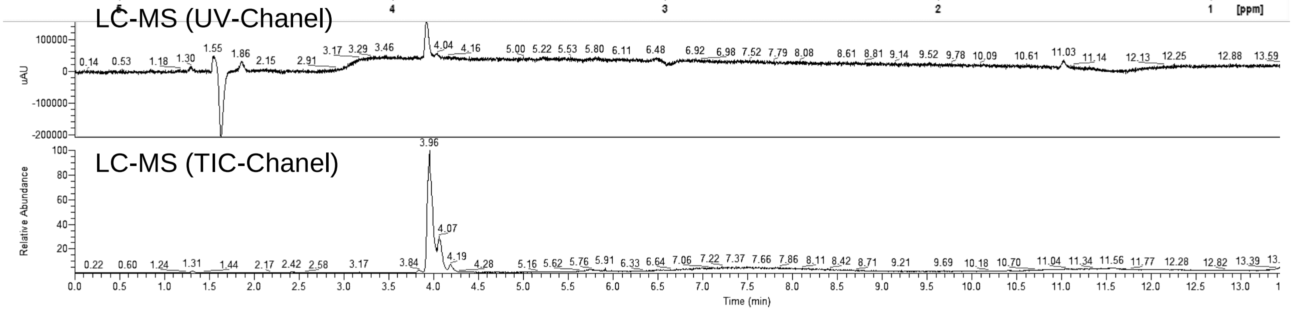

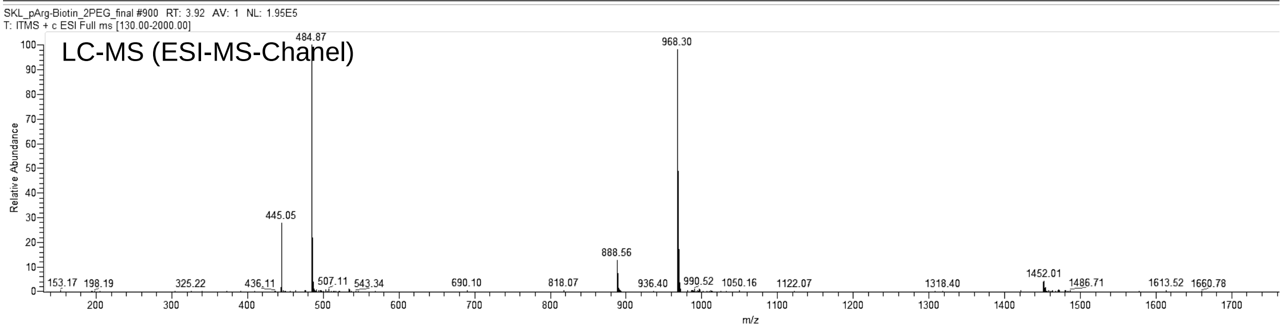

BacPROTAC-1b

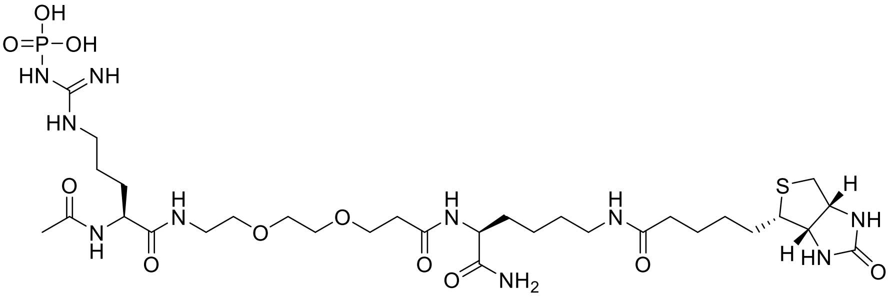

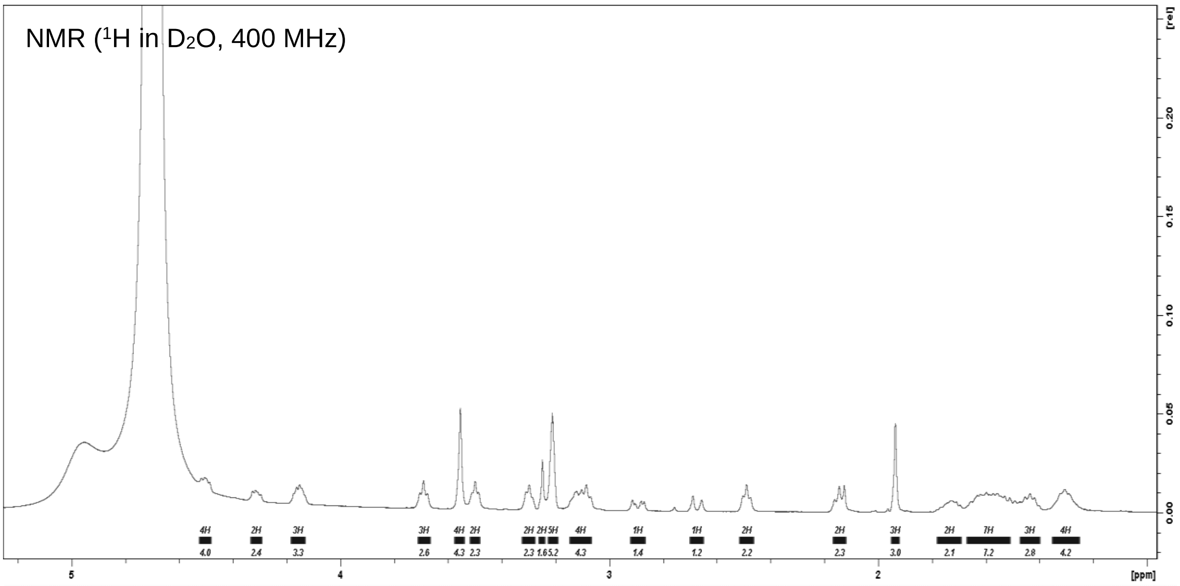

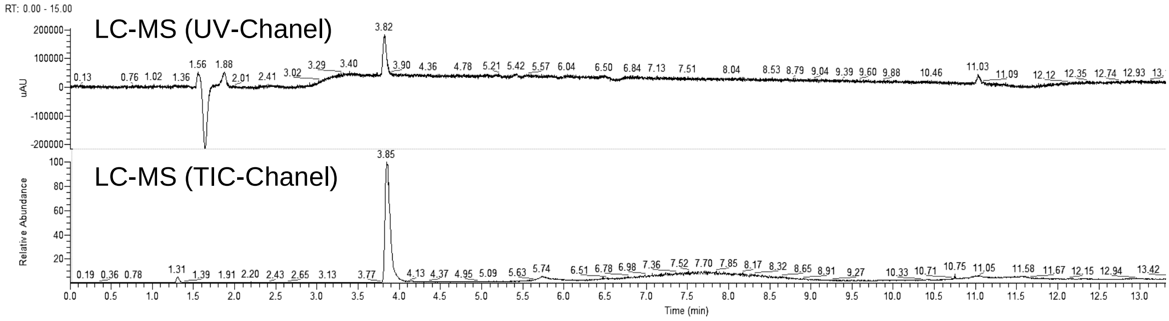

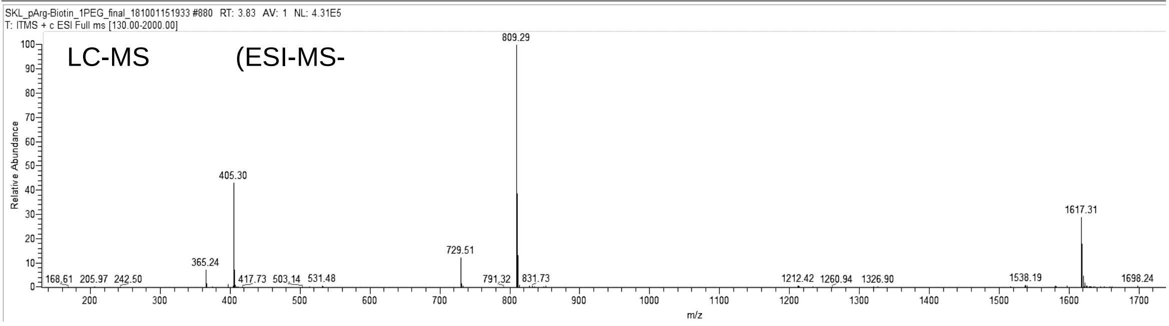

BacPROTAC-1c

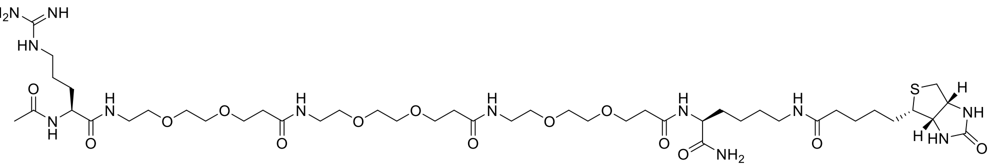

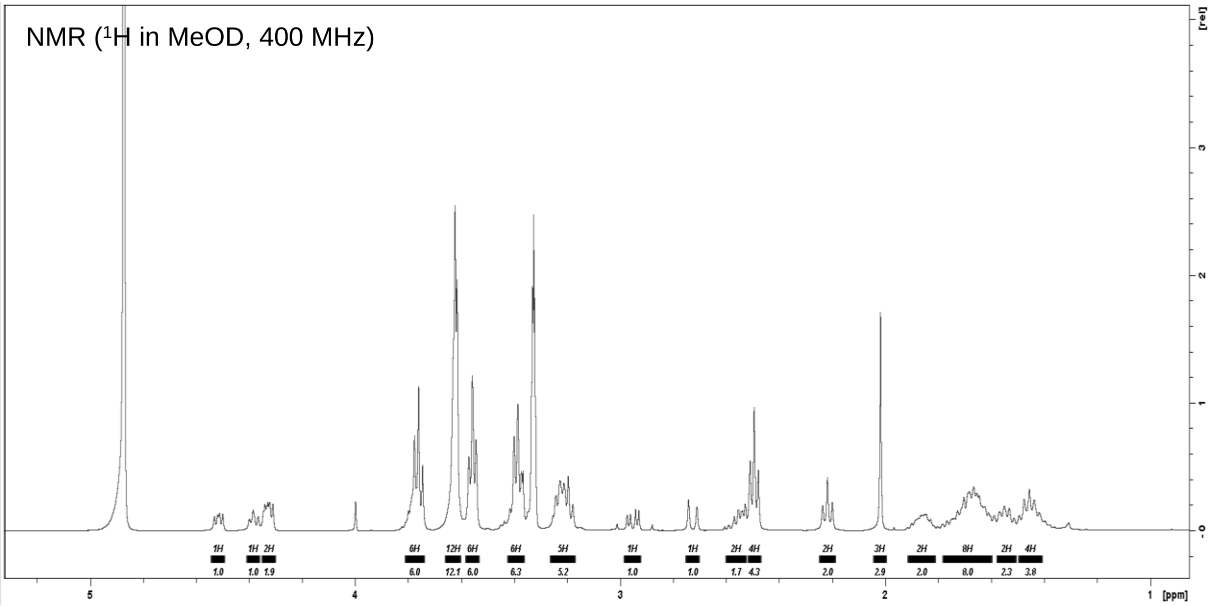

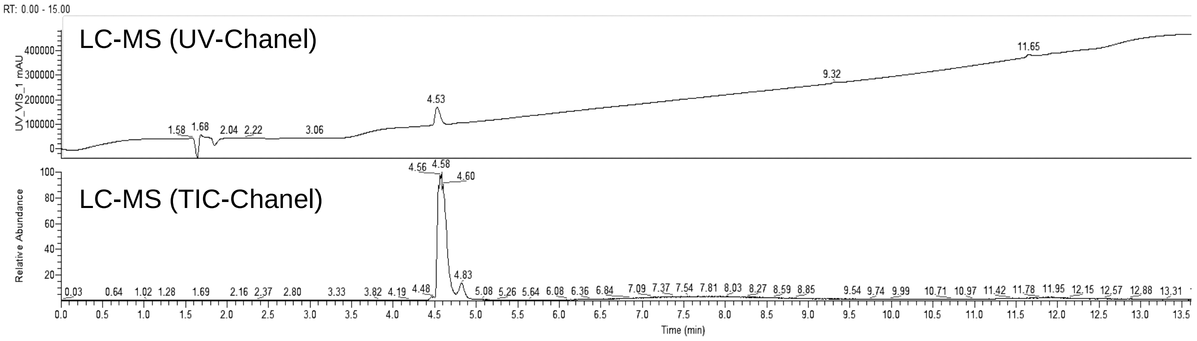

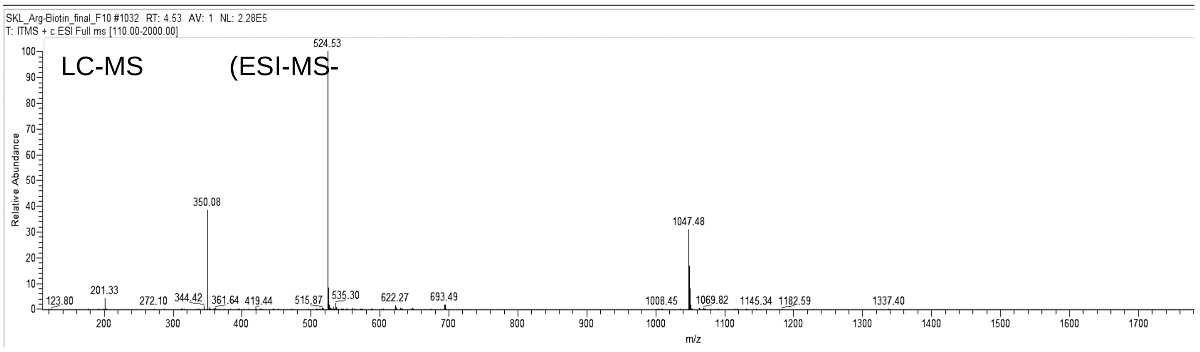

BacPROTAC-2

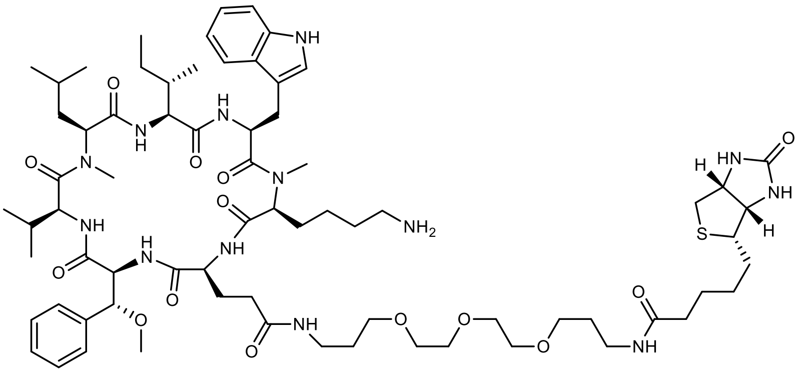

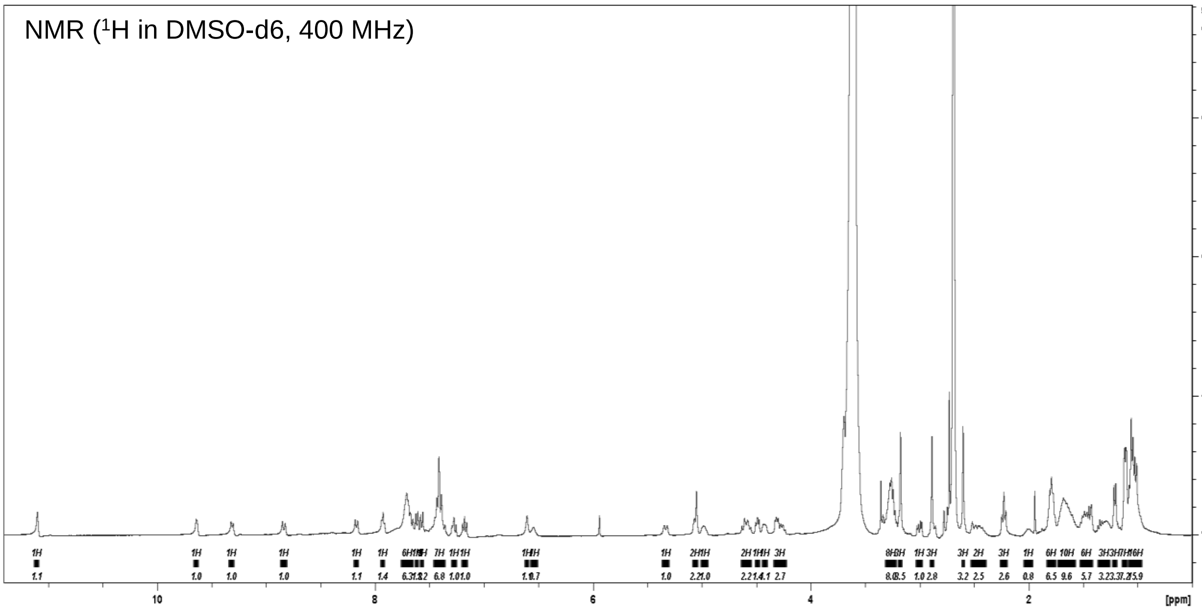
NMR (1H in DMSO-d6, 400 MHz)

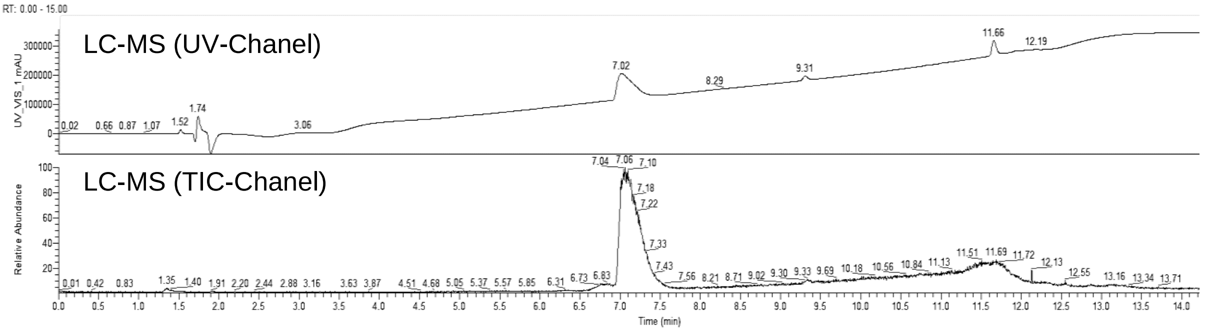
LC-MS (UV-Chanel)
LC-MS (TIC-Chanel)

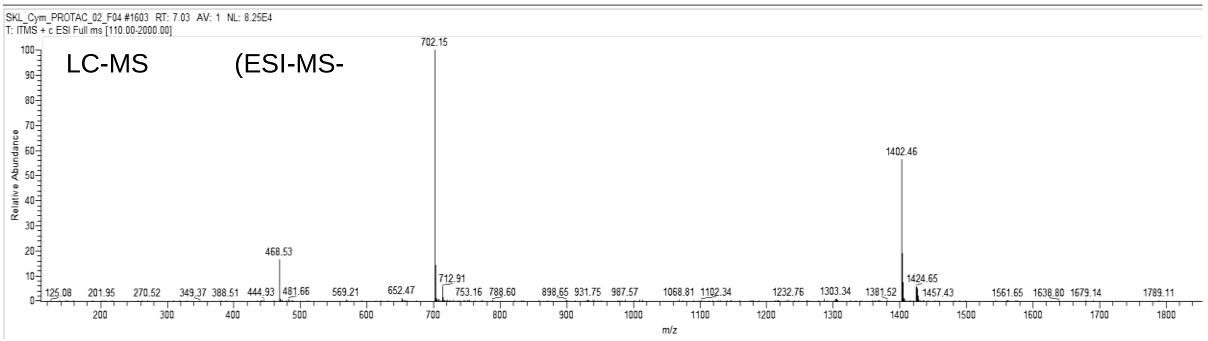
LC-MS (ESI-MS-

BacPROTAC-3

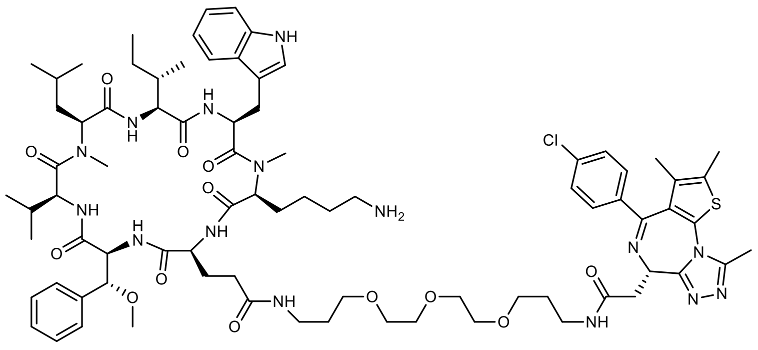

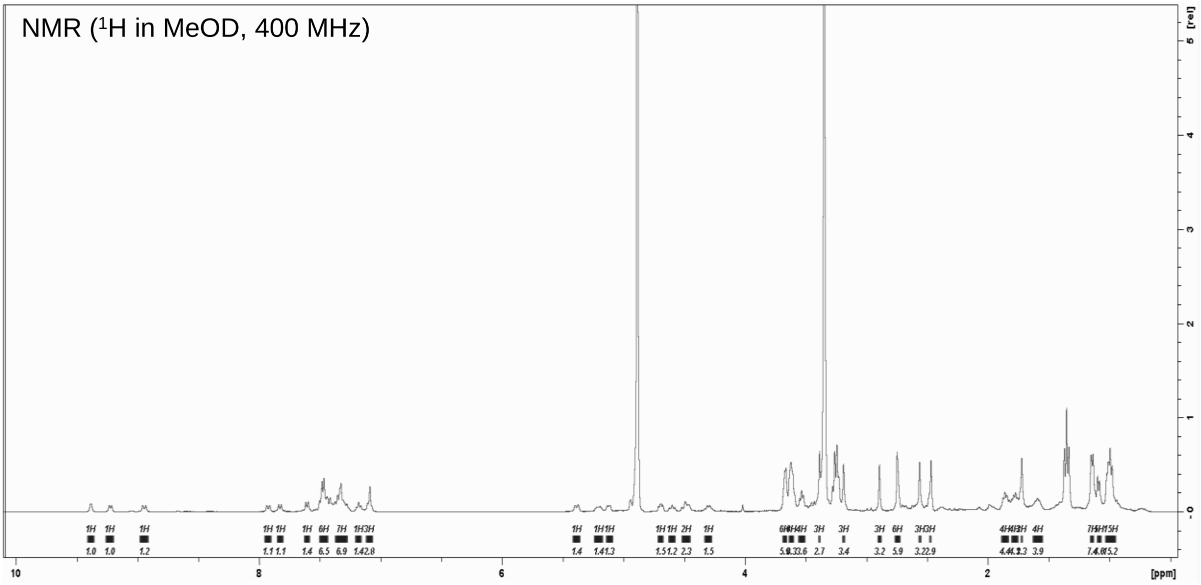
NMR (¹H in MeOD, 400 MHz)
[ppm]
[rel]

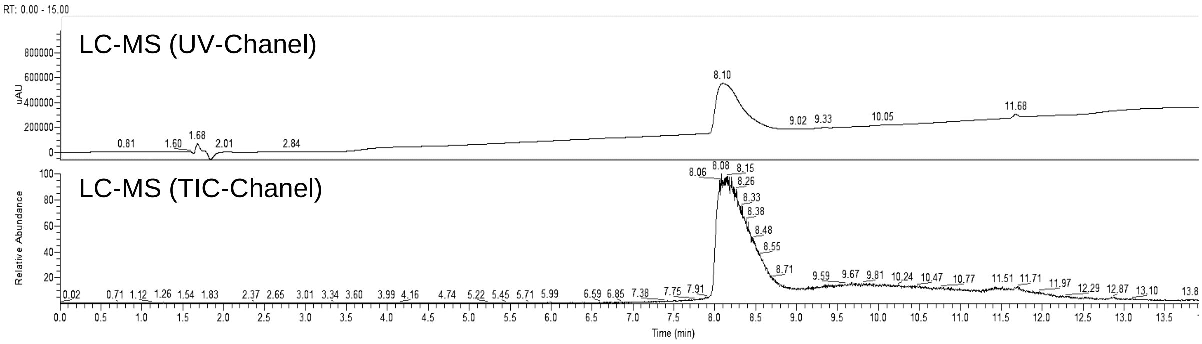
RT: 0.00 - 15.00
LC-MS (UV-Chanel)
uAU
8.10
11.68
LC-MS (TIC-Chanel)
Relative Abundance
Time (min)

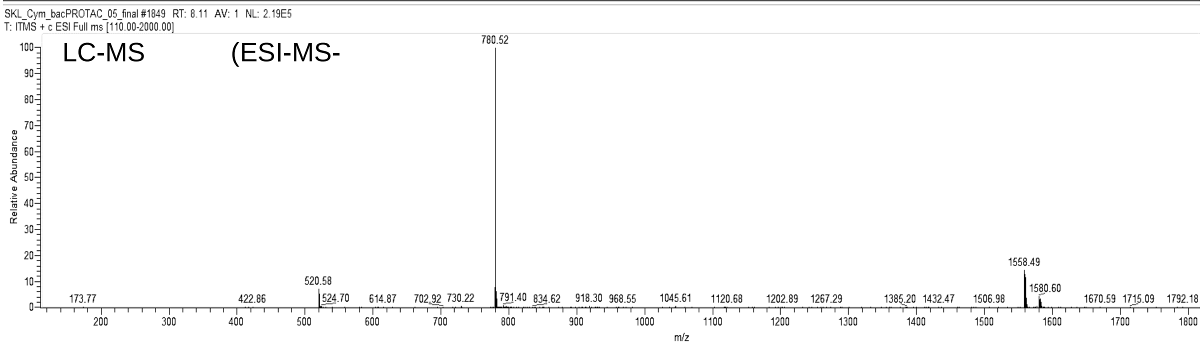
SKL_Cym_bacPROTAC_05_final #1849 RT: 8.11 AV: 1 NL: 2.19E5
T: ITMS + c ESI Full ms [110.00-2000.00]
LC-MS (ESI-MS-
780.52
1558.49
520.58
Relative Abundance
m/z

BacPROTAC-3a

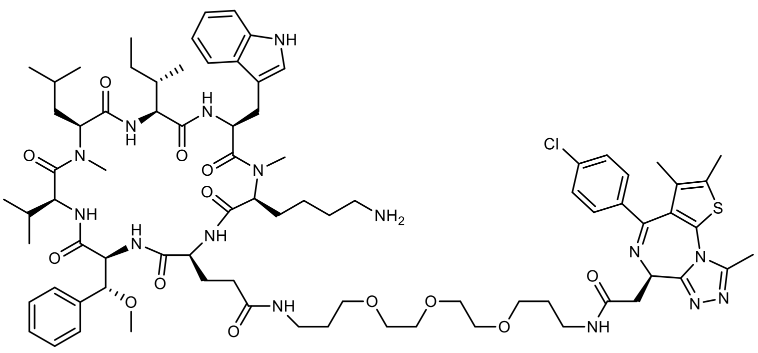

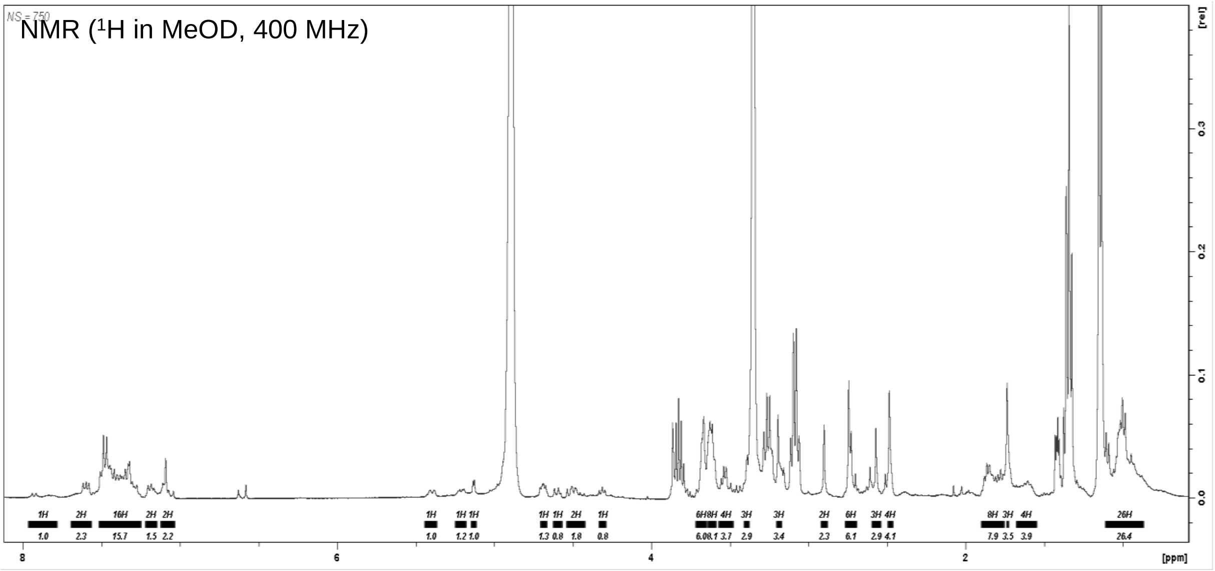

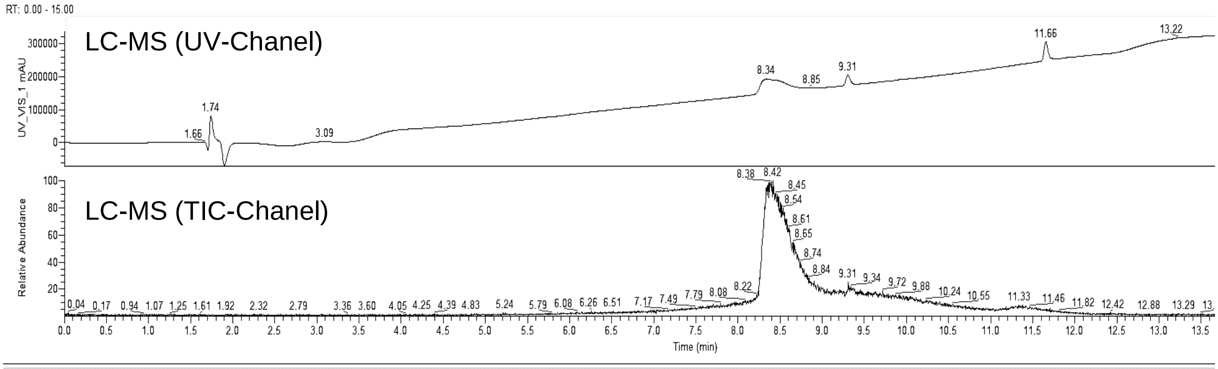

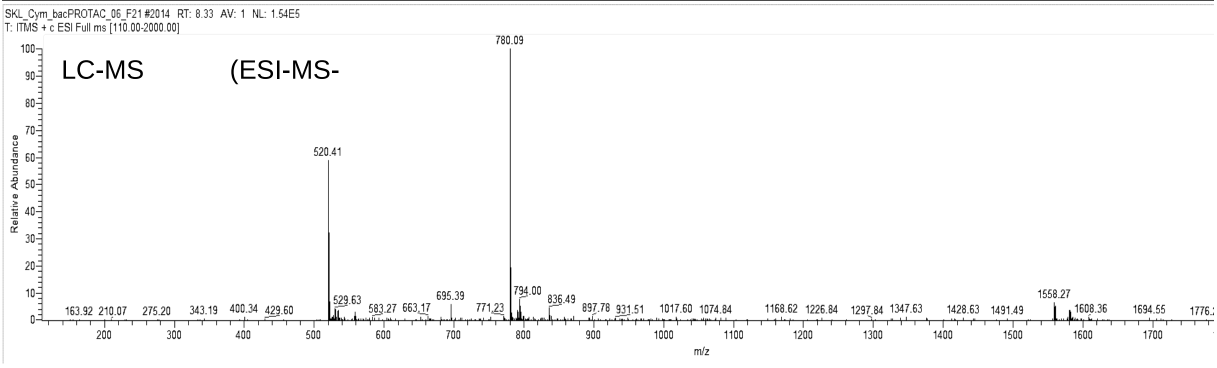

SI-15

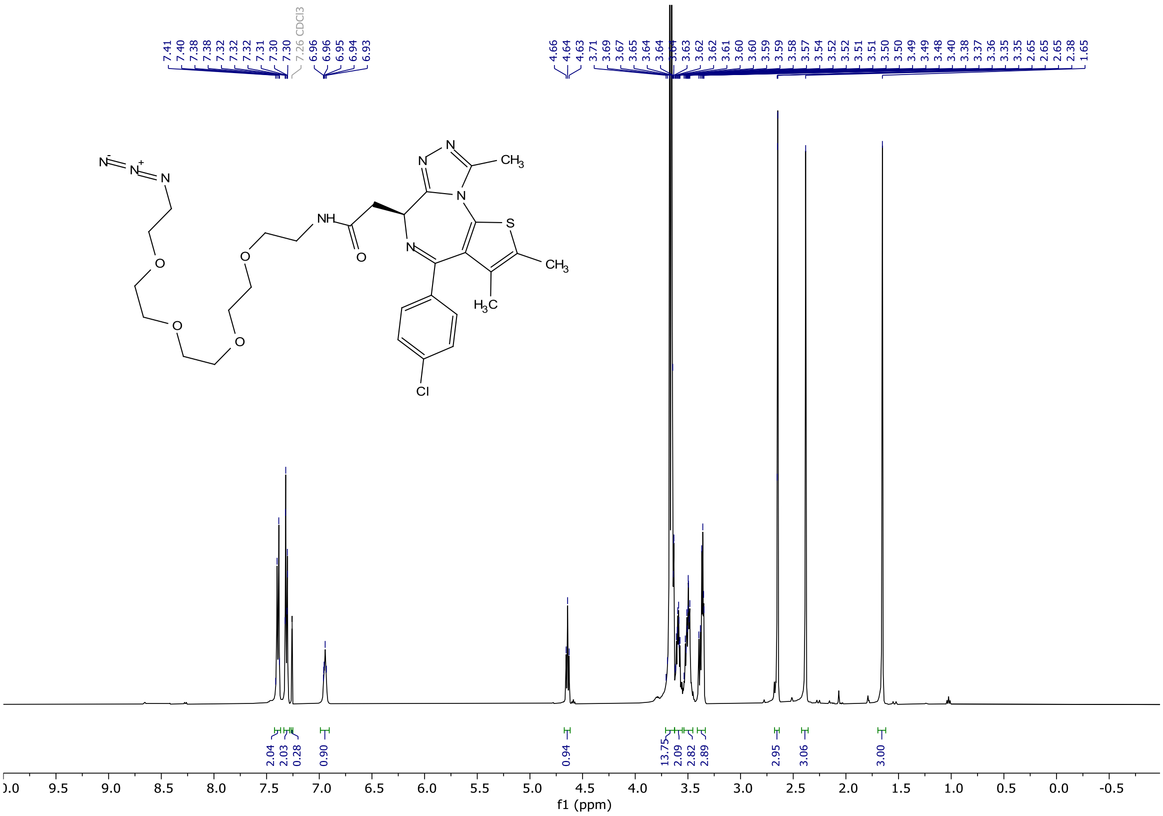

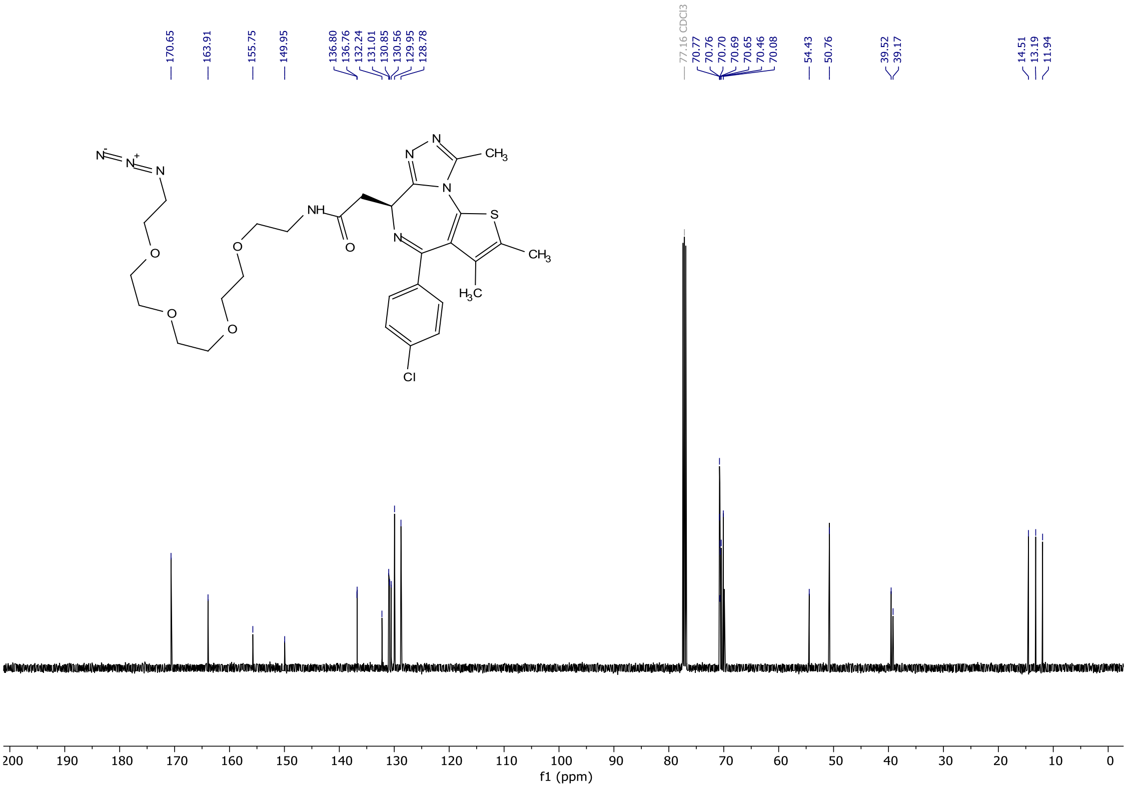

BacPROTAC-4

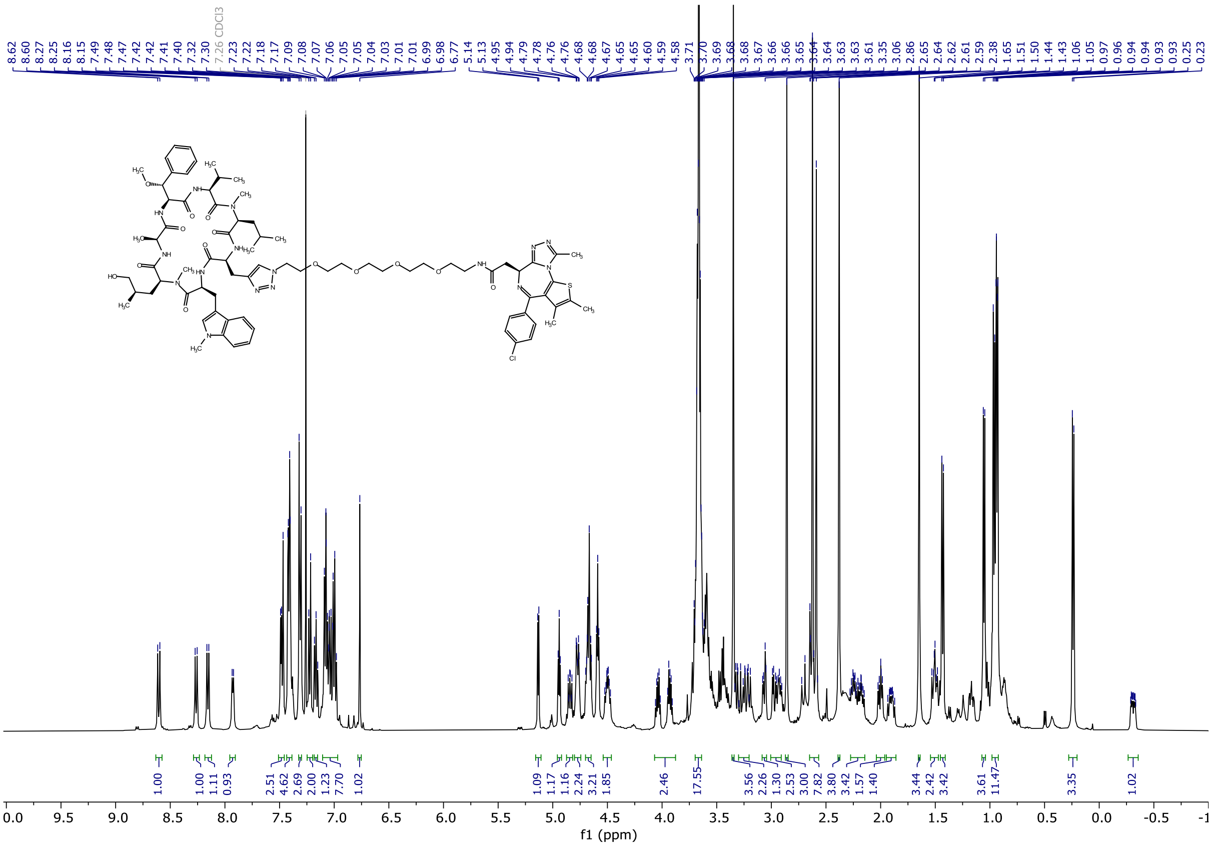

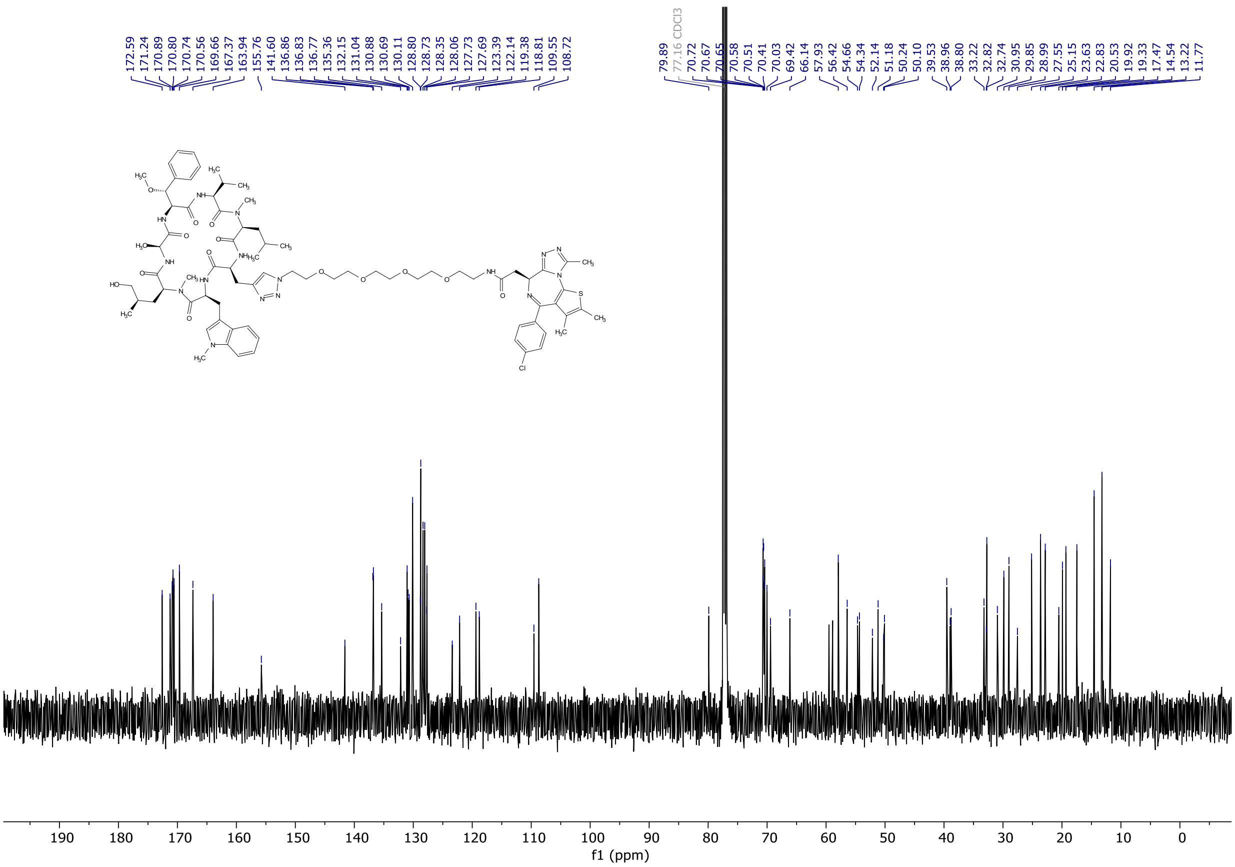

<Chromatogram>

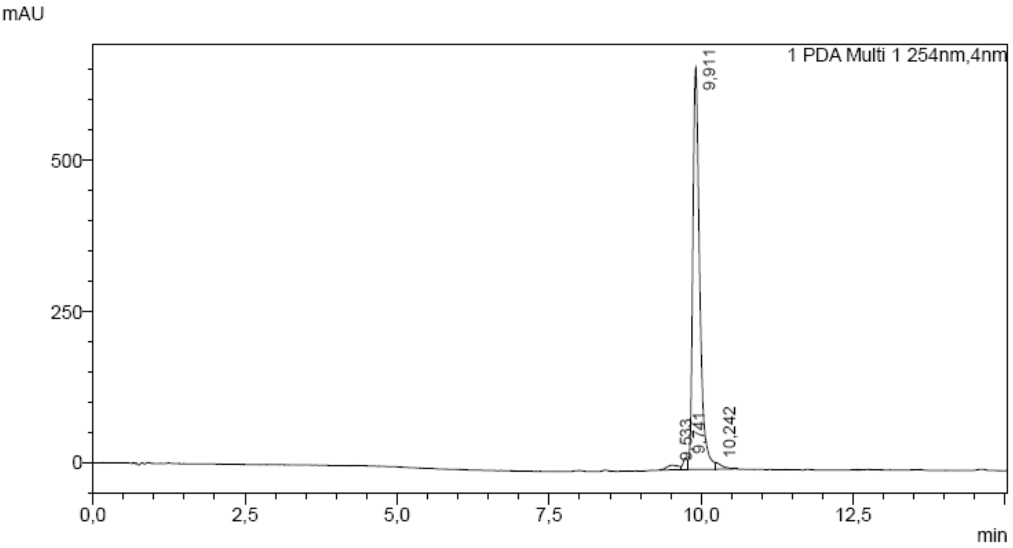

<Peak Table>

PDA Ch1 254nm

Peak#	Ret. Time	Area%
1	9,533	1,849
2	9,741	1,780
3	9,911	94,850
4	10,242	1,522
Total		100,000

BacPROTAC-4a

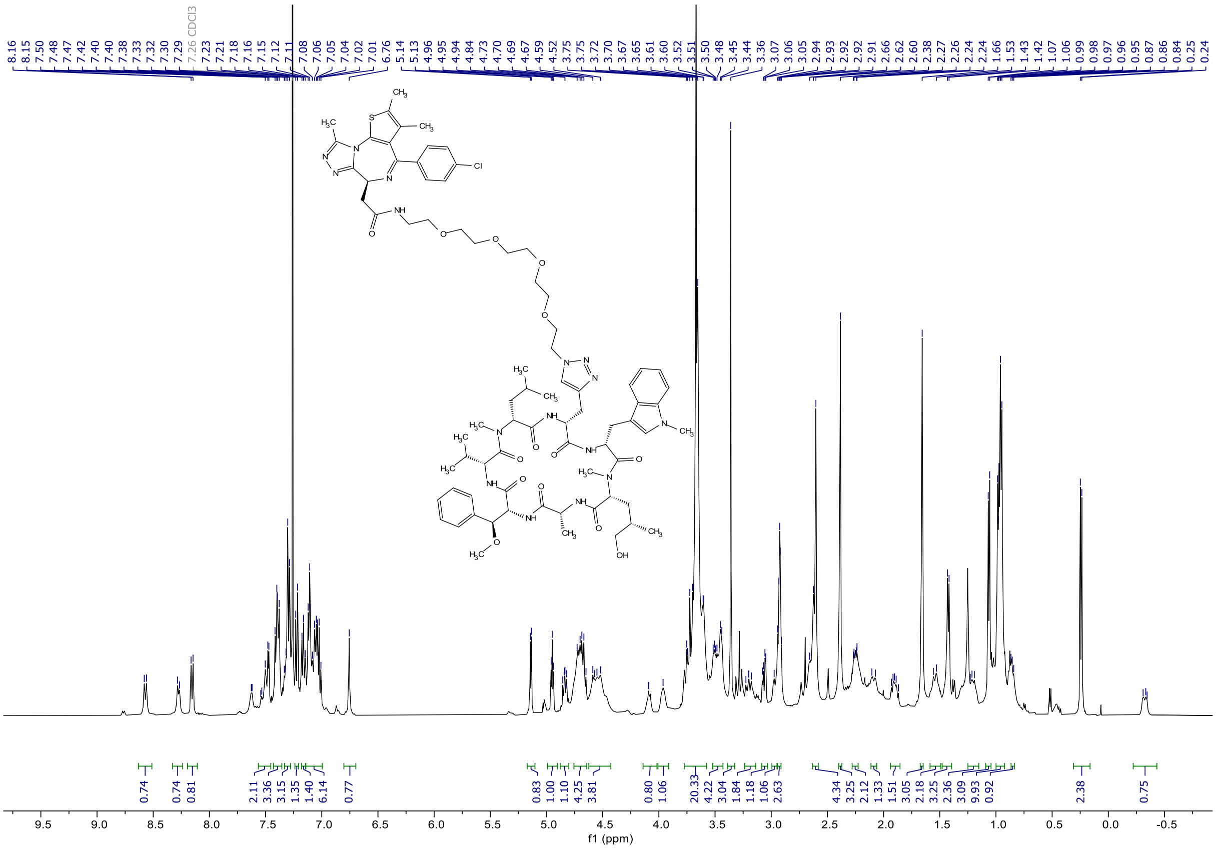

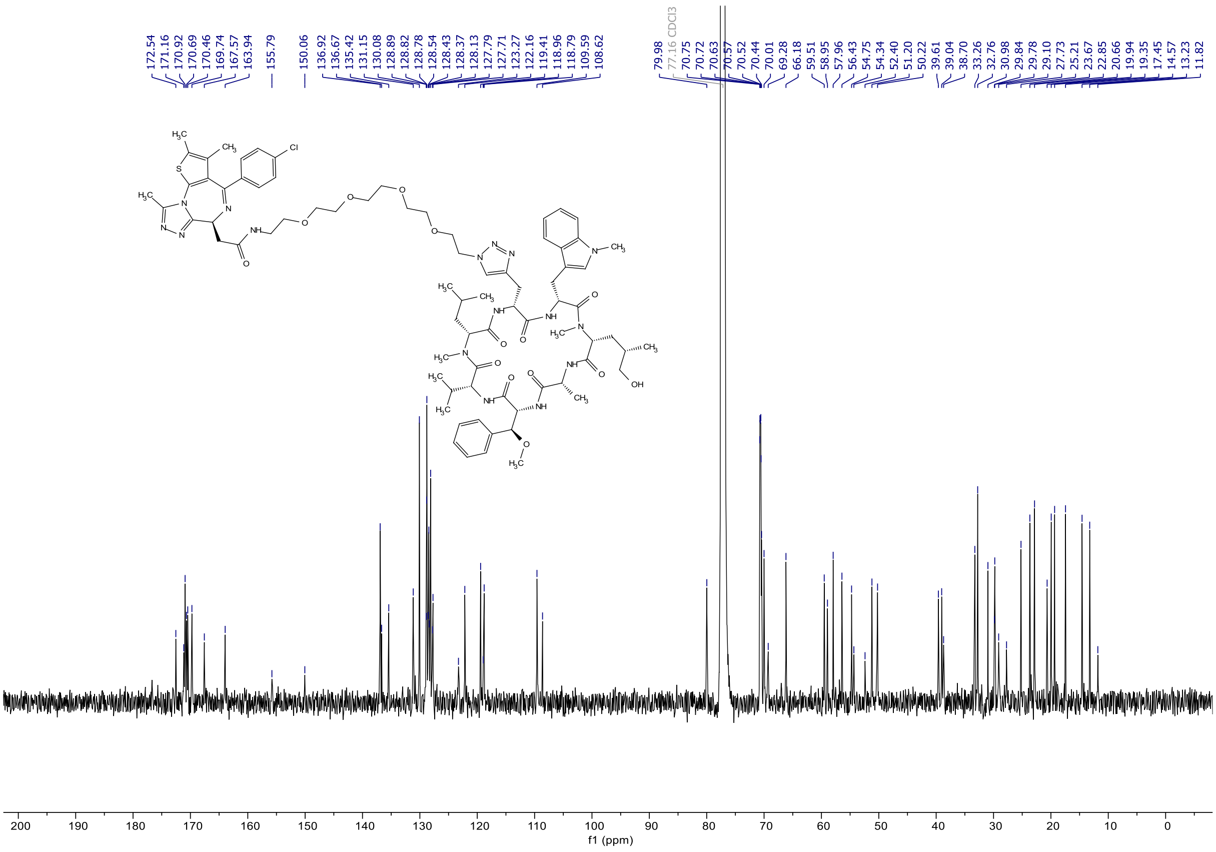

<Chromatogram>

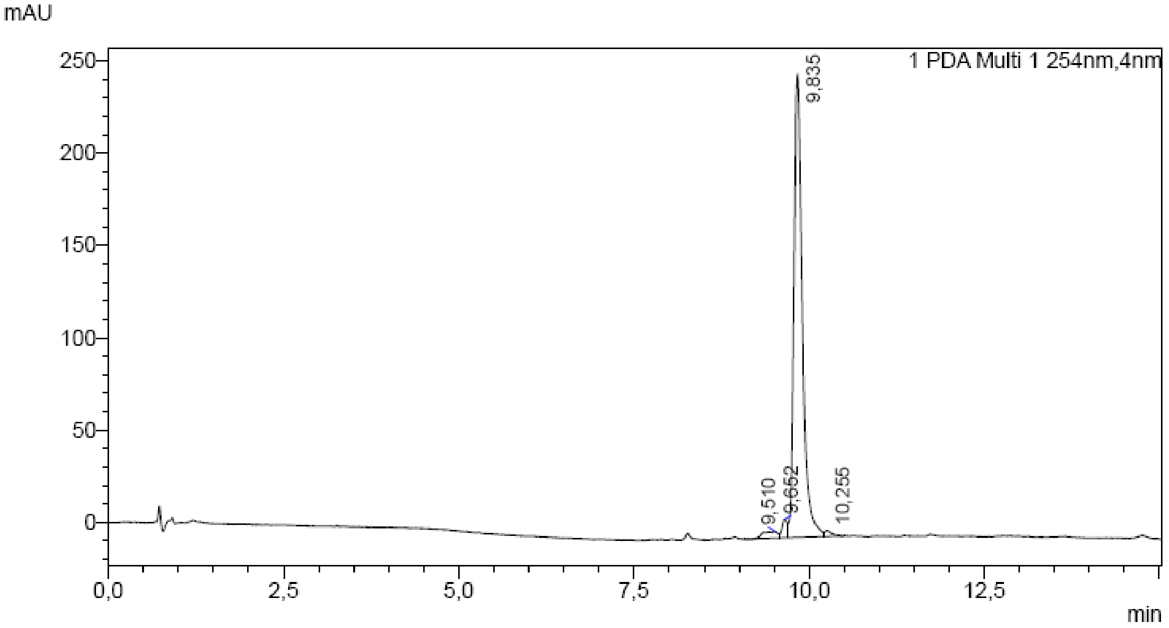

<Peak Table>

PDA Ch1 254nm

Peak#	Ret. Time	Area%
1	9,510	2,398
2	9,652	2,412
3	9,835	94,169
4	10,255	1,021
Total		100,000

SI-18

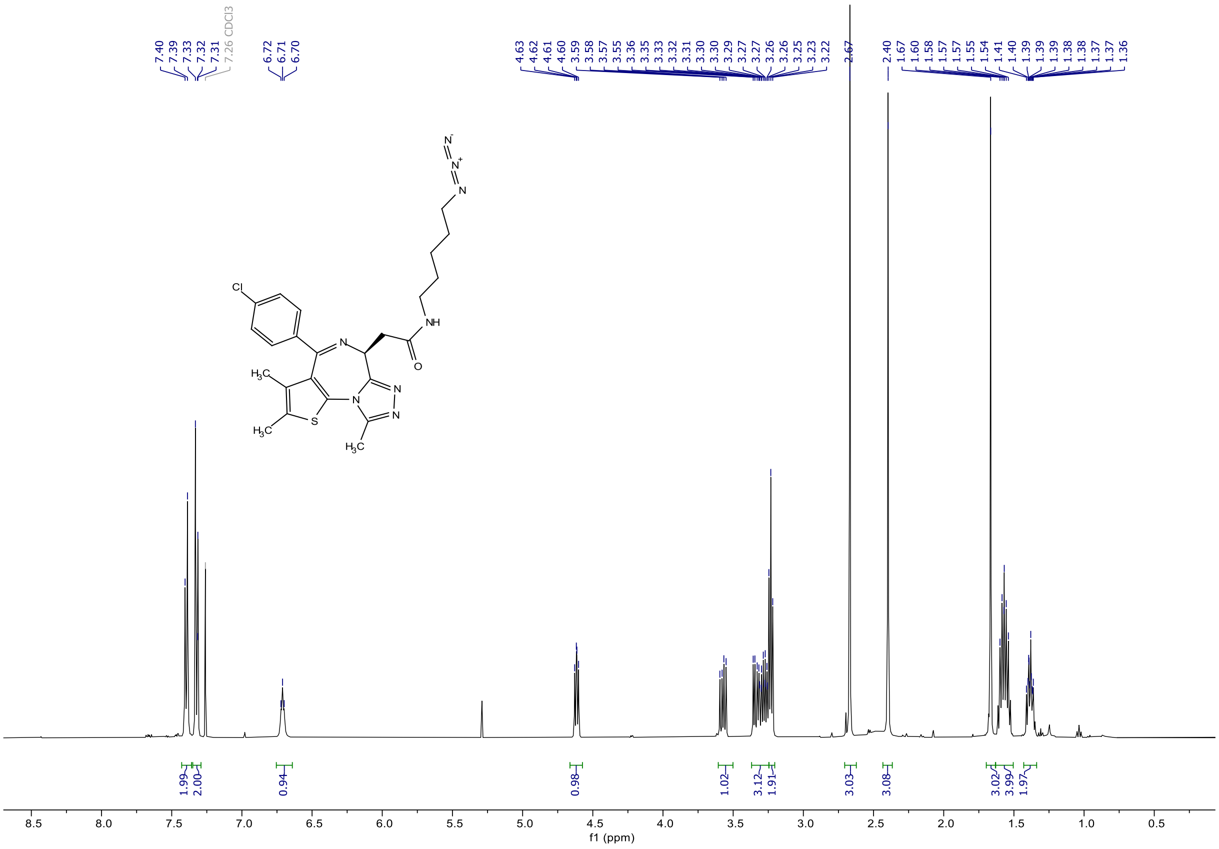

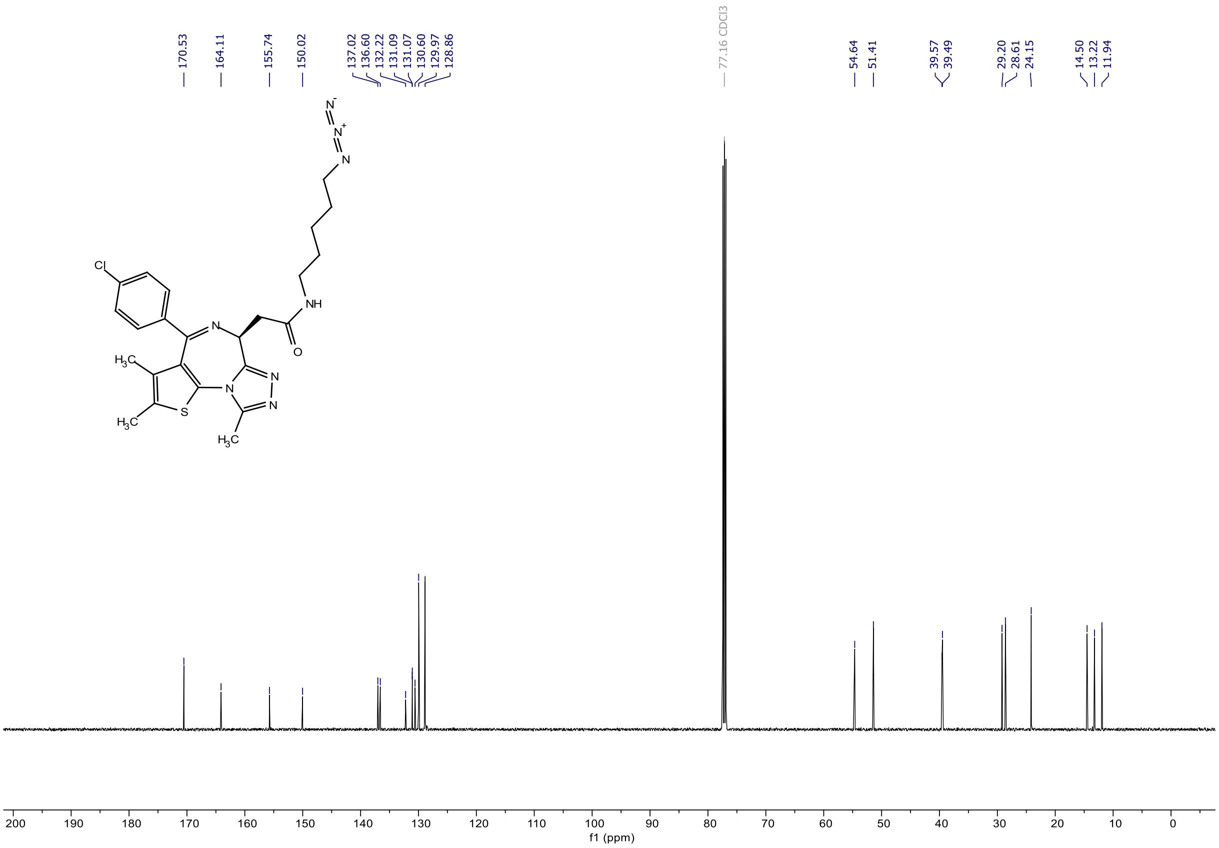

BacPROTAC-5

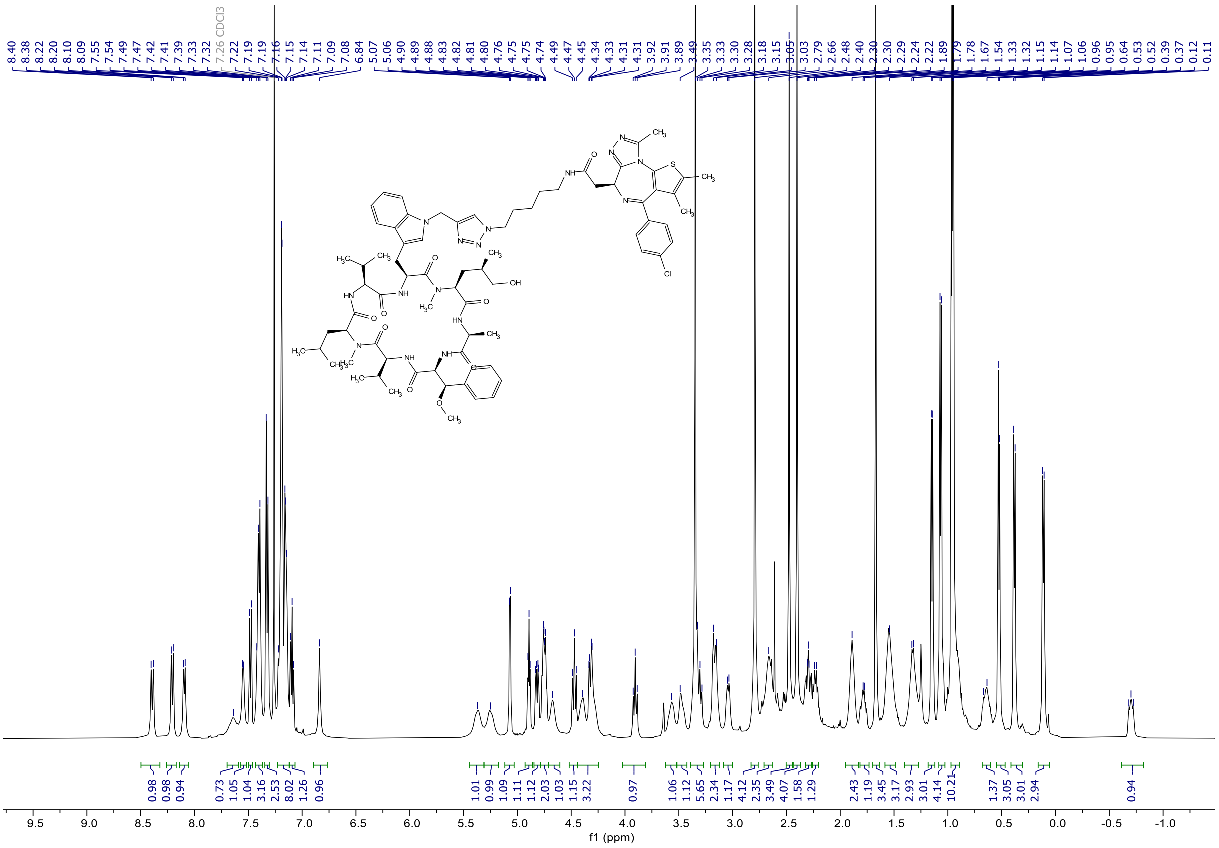

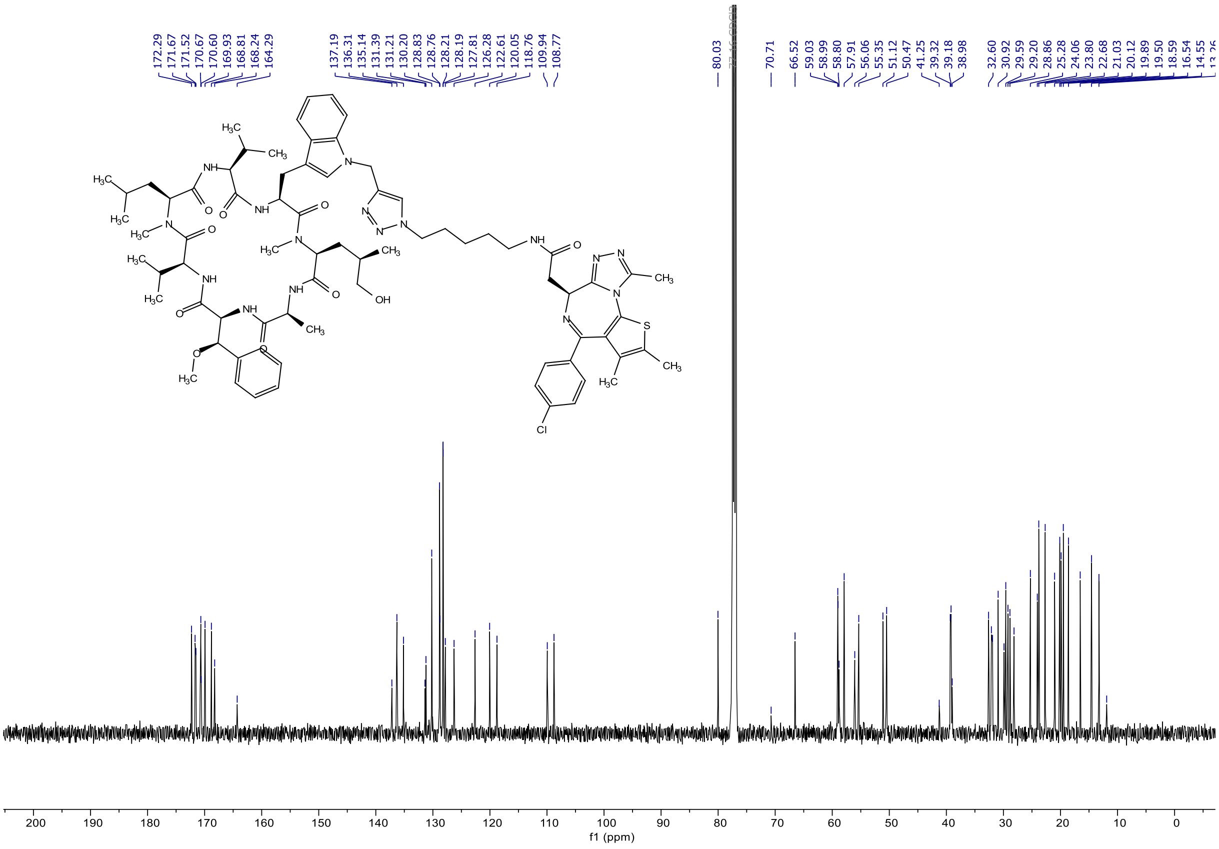

<Chromatogram>

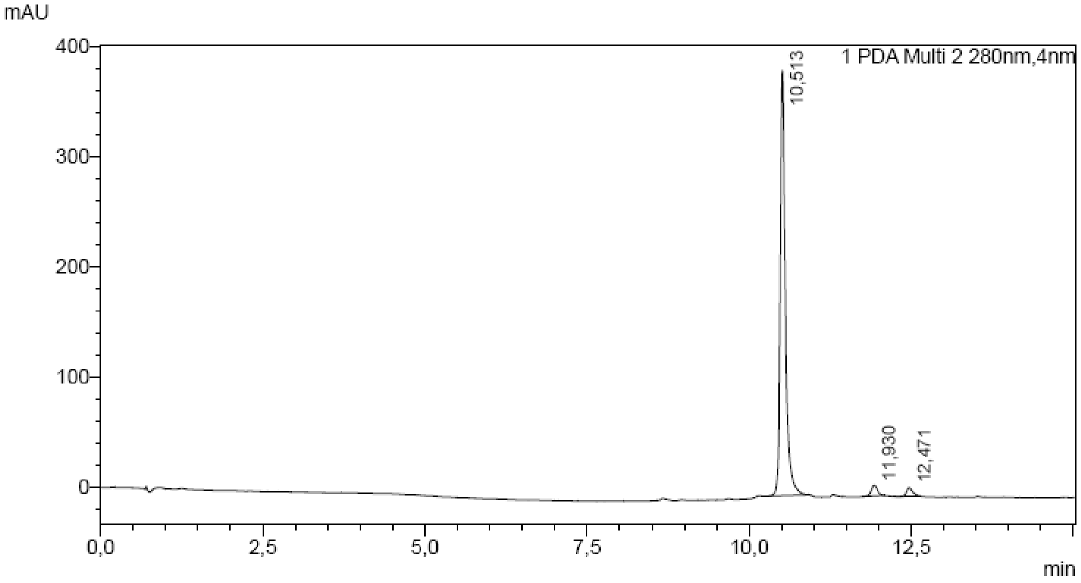

<Peak Table>

PDA Ch2 280nm

Peak#	Ret. Time	Area%
1	10,513	95,140
2	11,930	2,742
3	12,471	2,118
Total		100,000

SI-20

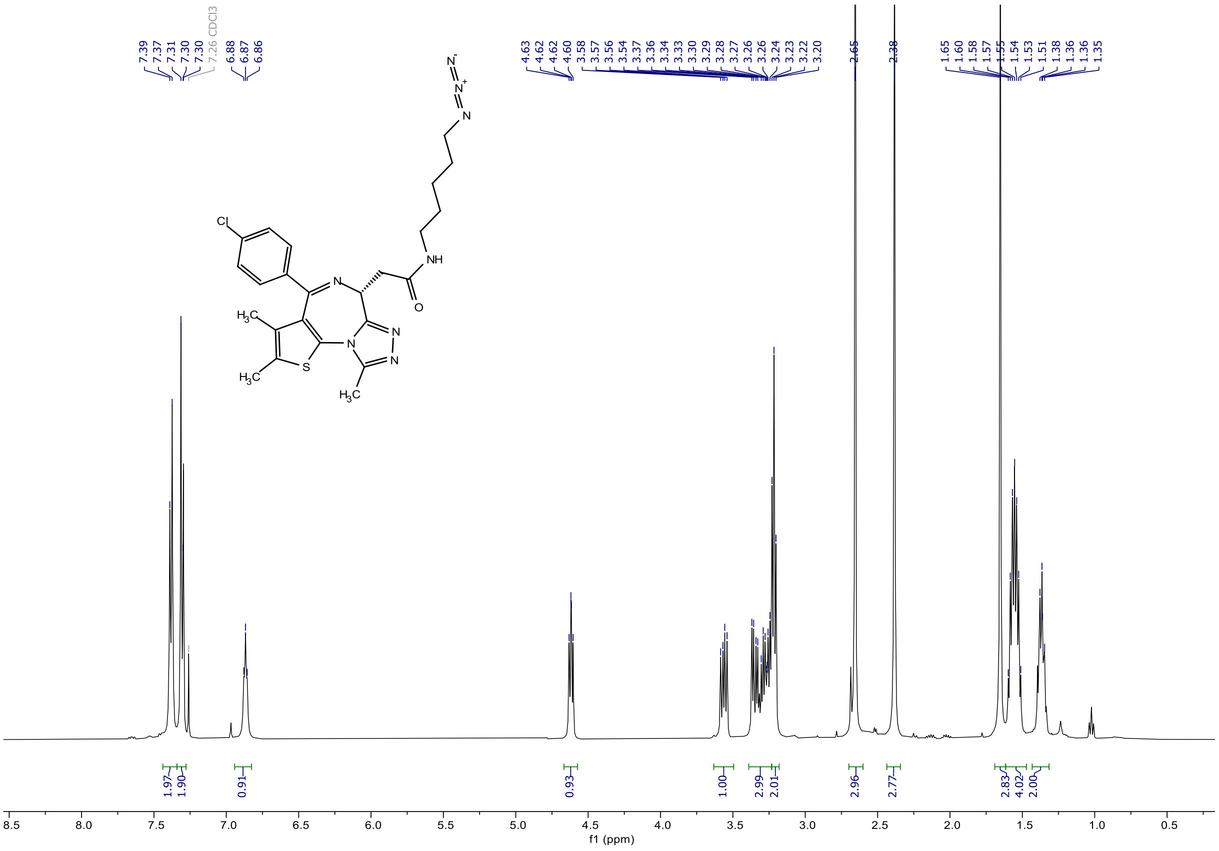

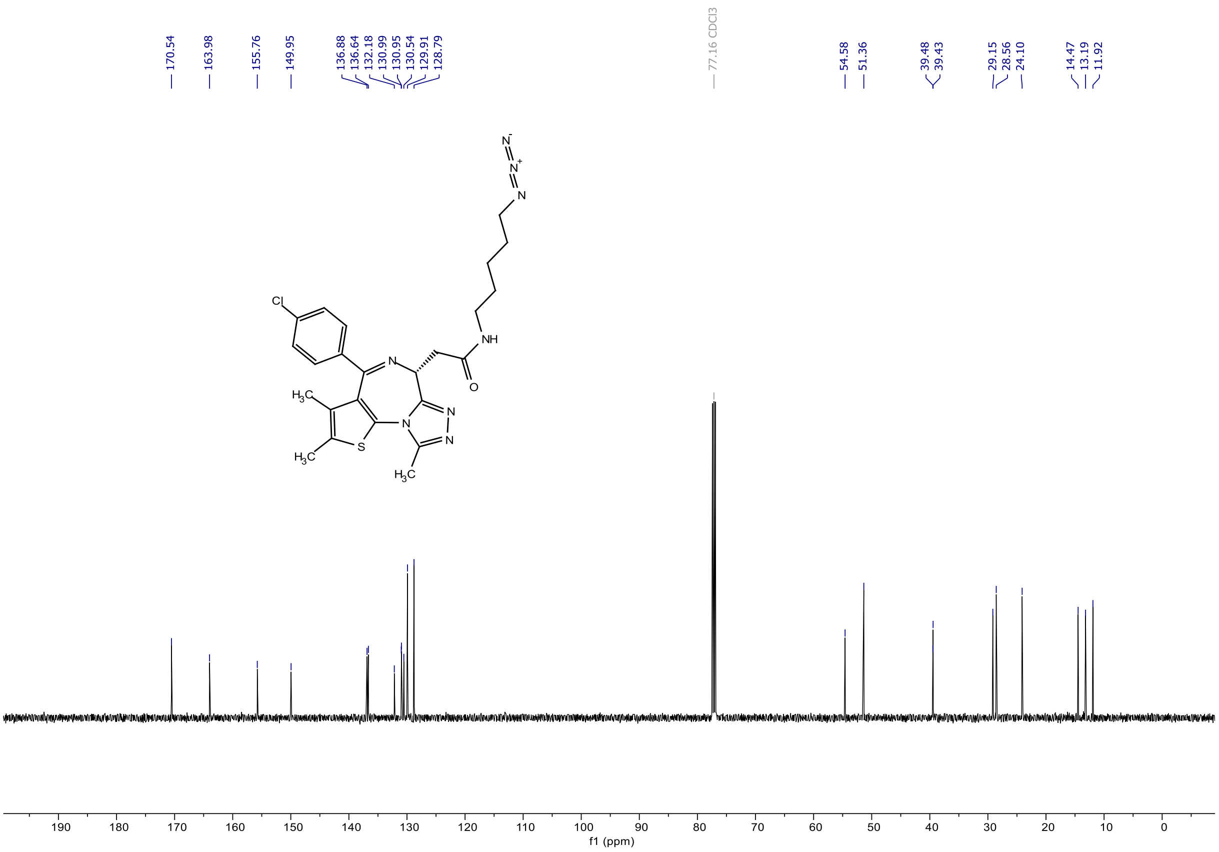

BacPROTAC-5a

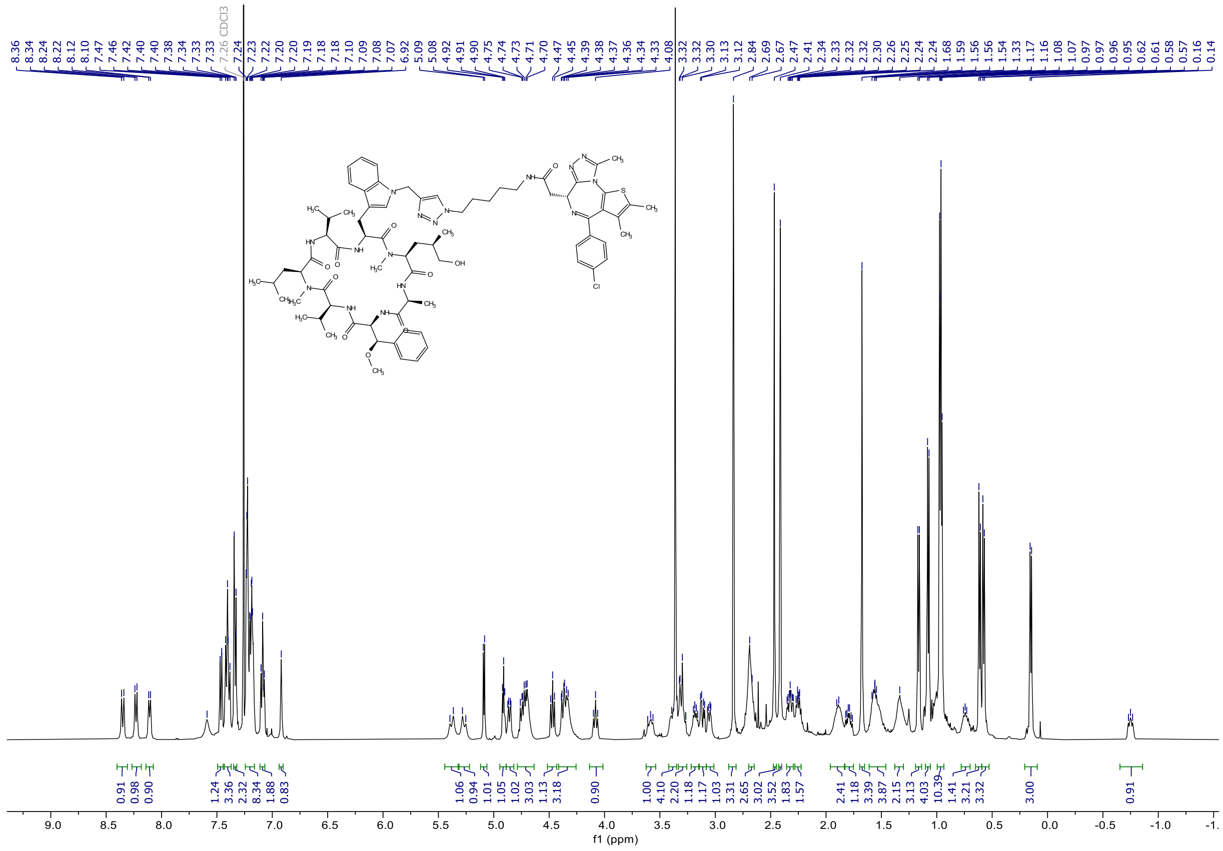

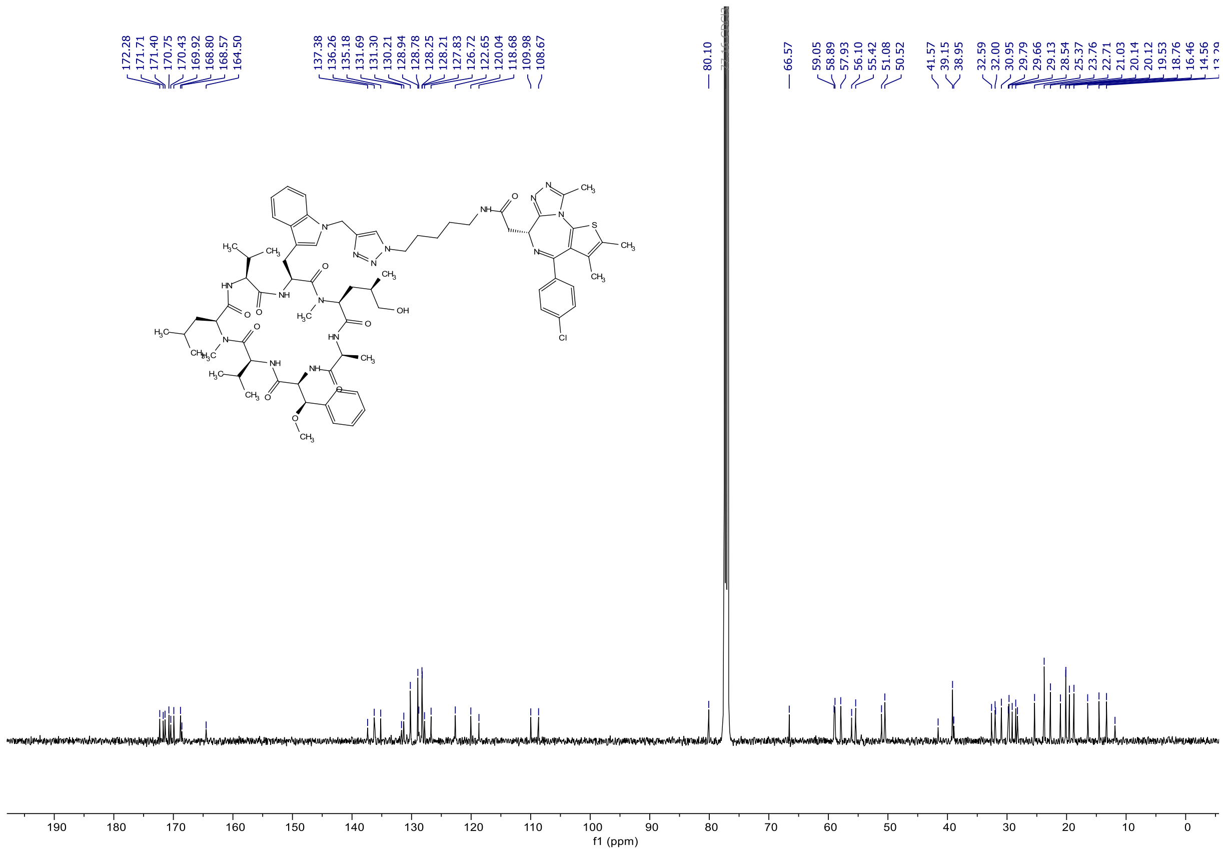

<Chromatogram>

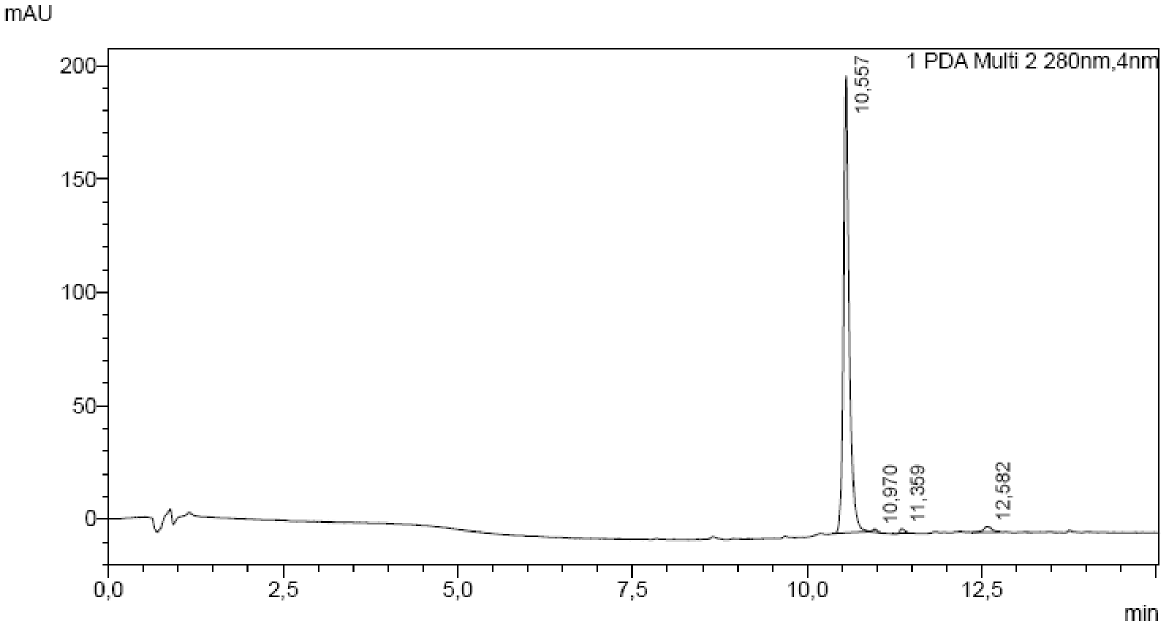

<Peak Table>

PDA Ch2 280nm

Peak#	Ret. Time	Area%
1	10,557	97,088
2	10,970	0,502
3	11,359	0,705
4	12,582	1,705
Total		100,000
